# Nickel and Copper in C–H Activation and Carbenoid Chemistry: A Descriptor-Based Comparative Analysis of Transition Metals

**DOI:** 10.1021/acs.jpca.5c07321

**Published:** 2026-01-27

**Authors:** Sasha Gazzari-Jara, Olivier Aroule, Guillaume Hoffmann, Henry Chermette, Christophe Morell, Barbara Herrera

**Affiliations:** † QTC, Departamento de Química Física, 28033 Pontificia Universidad Católica de Chile, Avenida Vicuña Mackenna, Santiago 4860, Chile; ‡ Université de Lyon, Université Lyon 1, Institut des Sciences Analytiques, UMR 5280, CNRS, 5 Rue de la Doua, Villeurbanne F-69100, France

## Abstract

A series of Fischer-type carbenoids of groups 10 and 11, bearing diverse electron-donating and electron-withdrawing substituents, were systematically analyzed using a descriptor-based framework grounded in Conceptual Density Functional Theory (CDFT), assessing whether shared electronic descriptors can rationalize the reaction profiles and reactivity in Ni­(II) and Cu­(I) complexes in a series of C–H activation reactions. The activation barriers for carbenoid insertion reactions were computed and correlated with reactivity indexes, including the Dual Descriptor and its Grand Canonical extensions based on softness and hardness (SGCDD and PGCDD). Although Ni carbenoids display slightly higher activation barriers than their Cu analogues, both metals exhibit parallel qualitative trends. The PGCDD descriptor showed the strongest predictive capability, yielding high correlations with computed barriers, particularly when refined through excited-state corrections using the Specific State Dual Descriptor (SSDD). Substituent effects especially mesomeric contributions from halogen groups were critically evaluated to establish a coherent electronic interpretation of reactivity. This unified descriptor-based framework provides a transferable methodology for rationalizing C–H activation mechanisms across transition metals, offering valuable insights for the predictive design of metal-carbenoid catalysts.

## Graphical abstract

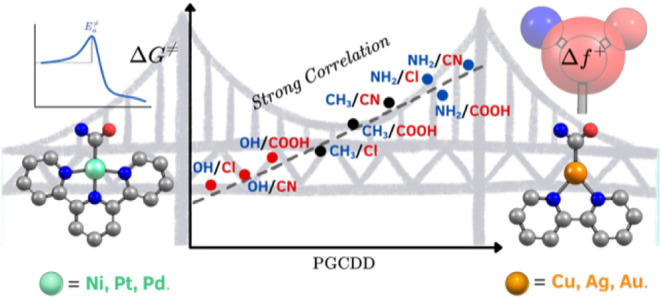

## 1 Introduction

The activation of C–H bonds remains one of the central challenges and opportunities in modern catalysis. The inertness of the C–H bond, arising from its high bond dissociation energy (100 kcal/mol) and low polarity, makes selective transformations particularly difficult.^1^ Among the various activation pathways, concerted insertion mechanisms are especially appealing due to their ability to achieve direct C–H functionalization with high atom economy.

Transition metal (TM) carbenoid intermediates have emerged as particularly powerful species for mediating C–H bond activation.^2^ These intermediates can be generated *in situ* either through decomposition of diazo precursors or through abstraction of leaving groups from α-functionalized organometallic substrates.^3,4^ Owing to their electrophilic nature, carbenoids participate in diverse transformations, including cyclopropanation,^5^ migratory insertion,^6^ and concerted insertion into C–H bonds.^7^ The tunability of carbenoid electrophilicity through substituent effects provides a systematic means to control reactivity and selectivity. In these processes, electrophilic intermediates interact with the filled σ­(C–H) orbital, lowering the activation barrier and enabling selective bond cleavage.^8^ A key factor governing reactivity in such pathways is the balance between electron-withdrawing and electron-donating substituents, which modulate the electrophilicity of the reactive intermediate.^9,10^

Noble metals such as Pd, Pt, Au, and Ag have historically played a central role in stabilizing and exploiting metal–carbenoid intermediates. Palladium carbenoids, for example, undergo C–H activation and direct arylation under mild conditions.^11–13^ Gold carbenoids have been structurally characterized, revealing their highly electrophilic nature and reactivity patterns.^14^ Silver carbenoids derived from diazo precursors also display strong electrophilic character, with substituent effects playing a critical role in their reactivity.^15^ Platinum complexes have likewise been reported to form stable Fischer-type carbenoid intermediates that engage in C–H bond transformations with high selectivity.^16^ Collectively, these studies provide a robust foundation for understanding carbenoid chemistry, though the scarcity and high cost of noble metals impose significant limitations for sustainable catalysis.

In contrast, first-row transition metals offer an attractive alternative owing to their greater abundance and lower cost. Copper-based carbenoids in particular are well-characterized and provide a valuable framework for rationalizing reactivity trends.^17–19^ Cu­(I) species form well-defined Fischer-type carbenoids,^20–22^ and conceptual DFT descriptors have been successfully applied to rationalize their electrophilicity and selectivity.^23,24^

Nickel, another first-row transition metal, offers unique opportunities in this context. Its abundance, affordability, and adaptable electronic structure position it as an attractive alternative to Pd and Pt.^25–28^ Both Cu­(I) (d^10^) and Ni­(II) (d^8^) species can stabilize Fischer-type carbenoids through closed-shell electronic configurations,^29^ suggesting mechanistic parallels that motivate comparative study. Despite these similarities, the reactivity of Ni carbenoids remains underexplored relative to their Cu and noble-metal counterparts. Establishing whether conceptual frameworks developed for Cu can be transferred to Ni is essential both for elucidating fundamental differences between first-row and late transition metals and for expanding the predictive design of Ni-based catalysts.

In the present study, we computationally investigate the C–H activation via Fischer-type carbenoids bearing electronically distinct substituents. Using Density Functional Theory (DFT) at the M06-2X/cc-pVDZ/LANL2DZ level, we examine the concerted insertion pathway via a σ-complex transition state. Our aim is to assess whether conceptual DFT descriptors can serve as predictive markers for Ni-carbenoid reactivity, and to determine whether structure–reactivity trends established for Cu carbenoids and previously demonstrated for noble metals such as Pd, Pt, Au, and Ag extend to Ni.

## 2 Theoretical Background

### 2.1 Conceptual DFT (CDFT)

Reactivity analysis was carried out using conceptual DFT (CDFT), a branch of density functional theory that describes response functions to perturbations in the number of particles (*N*) or the external potential υ­(**r**).^30,31^ Within this framework, a wide variety of local and global electronic properties describe the ability of molecular systems to undergo chemical changes or modifications in their electron density.

The chemical potential (μ) is the first energy response to a perturbation in the number of electrons at a constant external potential. It indicates the escaping tendency of electrons and is associated with the negative value of the electronegativity (χ). The molecular hardness (η) is defined as the second perturbation of the energy to the number of electrons and indicates the tendency of an electronic cloud to modify its distribution:^32^

1 $$\mu ={\left(\frac{\partial E}{\partial N}\right)}_{v\left(r\right)},,\eta ={\left(\frac{\partial^{2}E}{\partial N^{2}}\right)}_{v\left(r\right)}={\left(\frac{\partial \mu }{\partial N}\right)}_{v\left(r\right)}$$

As the energy is discontinuous with *N*, the operational definitions of these indexes are given by the finite difference approximation method, where μ and η can be approximated to ionization potentials (IP) and electron affinities (EA), and the Koopmans theorem to the energies of the HOMO (ε_H_) and LUMO (ε_L_):^33,34^

2 $$\mu \approx -\frac{\mathrm{IP}+\mathrm{EA}}{2}\approx \frac{\varepsilon_{H}+\varepsilon_{L}}{2},,\eta \approx \mathrm{IP}-\mathrm{EA}\approx \varepsilon_{L}-\varepsilon_{H}$$

On the other hand, the Fukui function provides a practical way to analyze the reactivity at specific sites of the molecule to study its selectivity^33^ and is defined as the change in density due to a perturbation in the external potential:

3 $$f\left(r\right)={\left(\frac{\partial \rho \left(r\right)}{\partial N}\right)}_{v\left(r\right)}=\left(\frac{\delta^{2}E}{\delta v\left(r\right)\partial N}\right)$$

Within the finite-difference approximation, the nucleophilic Fukui function *f* ^–^(**r**) and the electrophilic Fukui function *f* ^+^(**r**) can be defined by the left and right derivatives, which predict nucleophilic and electrophilic sites in a molecular system.^35^ It is possible to associate *f* ^–^(**r**) and *f* ^+^(**r**) of an *N*-electron species to the density of the HOMO [ρ_*H*_(**r**)] and LUMO [ρ_*L*_(**r**)], by using the frozen core approximation:^33^

4 $$f^{+}\left(r\right)\approx \rho_{L}\left(r\right),,f^{-}\left(r\right)\approx \rho_{H}\left(r\right)$$

Furthermore, the Dual Descriptor Δ*f*(**r**) allows for the simultaneous identification of nucleophilic and electrophilic regions based on the difference of ρ_L_(**r**) and ρ_H_(**r**).^36^ The operational definition of this index is given by

5 $$\Delta f\left(r\right)=f^{+}\left(r\right)-f^{-}\left(r\right)\approx \rho_{L}\left(r\right)-\rho_{H}\left(r\right)$$

This Δ*f*(**r**) helps in identifying simultaneously both nucleophilic and electrophilic regions in the molecule, providing valuable information about the molecular sites that are more susceptible to nucleophilic or electrophilic attacks. Positive values of Δ*f*(**r**) indicate electrophilic regions (susceptible to nucleophilic attack), whereas negative values highlight nucleophilic regions (prone to electrophilic attack).

To obtain site-specific quantitative information, the Δ*f*(**r**) can be spatially integrated over defined regions *D*_*i*_ of the molecule, leading to a condensed descriptor:^37,38^

6 $$\Delta f\left(D_{i}\right)=\int_{D_{i}}\Delta f\left(r\right)d^{3}r$$

This domain-based integration allows for the evaluation of local reactivity indices while retaining the interpretability of the full-space function using the OnGrid method.^38,39^

The Grand Canonical Dual Descriptor (GCDD)^40,41^ extends this framework by incorporating global softness *S* (inverse of chemical hardness, *S* = 1/η) as

7 $$\mathrm{SGCDD}=S\Delta f=S\Delta f\left(D_{i}\right)$$

The Grand Canonical ensemble is particularly useful for examining systems where the number of electrons can change, as it allows for the exchange of particles with an external reservoir. By regulating the electronic chemical potential, rather than the total number of electrons, it ensures comparability in reactivity among various systems, despite their differing electron numbers. This method maintains uniformity in assessing reactivity descriptors regardless of variations in electron counts.

Furthermore, a new ensemble based on chemical hardness as a natural global variable using hypersoftness *P* (defined as the inverse of hyperhardness: *P* = 1/γ), where γ represents the hyperhardness, calculated using

8 $$\gamma =\varepsilon_{\mathrm{LUMO}}-2\varepsilon_{\mathrm{HOMO}}+\varepsilon_{\mathrm{HOMO}-1}$$

where *ε*_HOMO–1_ is the energy of the next-to-highest occupied molecular orbital, is used similarly to the SGCDD approach to obtain

9 $$\mathrm{PGCDD}=P\Delta f=P\Delta f\left(D_{i}\right)$$

In this new set, *P*Δ*f* is treated as a basic descriptor instead of a composite, making it comparable to the Grand Canonical ensemble.^42^

An orbital-specific extension of the Δ*f*(**r**) was adopted to include contributions from additional virtual orbitals beyond the LUMOs approach considers differences in electron density between the HOMO and a selected higher unoccupied orbital (LUMO + *n*):^43^

10 $${\Delta f\left(r\right)}_{\left(\mathrm{LUMO}+n\right)}=\rho_{\mathrm{LUMO}+n}\left(r\right)-\rho_{\mathrm{HOMO}}\left(r\right)$$

This generalization captures reactivity features associated with higher-energy electronic transitions, especially in cases where the LUMO does not sufficiently describe the relevant electrophilic behavior when the frontier molecular orbital theory preset tricky situations.

Finally, a more in-depth approach was employed using the Specific State Dual Descriptor (SSDD) approach to incorporate many-body and configuration-interaction effects. This descriptor is defined as the density difference between a selected excited state and the ground state:

11 $$\mathrm{SSDD}=\Delta f_{i}\left(r\right)=\rho_{i}\left(r\right)-\rho_{0}\left(r\right)$$

where ρ_*i*_(**r**) is the total electron density of the *i*-th excited state obtained via time-dependent DFT (TD-DFT), and ρ_0_(**r**) is the ground-state density. This formulation enables the analysis of reactivity patterns arising from specific electronic transitions, thereby extending the Δ*f*(**r**) concept into the excited-state regime.^44^

## 3 Computational Details

The systems analyzed in this study are based on TM carbenoids derived from the groups 10 (Ni, Pd, Pt) and 11 (Cu, Ag, Au). These complexes are coordinated with ligands such as 2,2′:6′,2″-terpyridine (tpy) and 2,2′-bipyridine (bpy), which have been selected to provide metal stability through a chelating effect.^45,46^ These carbenoids are functionalized with a representative selection of electron-donating (EDG) and electron-withdrawing groups (EWG), the selected EDGs such as OH, CH_3_, and NH_2_ donate electron density to the carbenoid carbon, reducing its electrophilicity, whereas EWGs such as Cl, COOH, and CN withdraw electron density, selected to represent a wide spectrum of electronic effects, including weak (CH_3_, Cl), moderate (OH, COOH), and strong (NH_2_, CN) donors and withdrawers as summarized in Scheme 1. These substituents were selected to modulate the electronic environment of the carbenoid and to assess their influence on the C–H activation process, including the moderately strong EWG (COCl) which will be discussed further into the manuscript, all the systems are presented in the Supporting Information as the Cartesian coordinates as presented in Table S10.

**Scheme 1:**
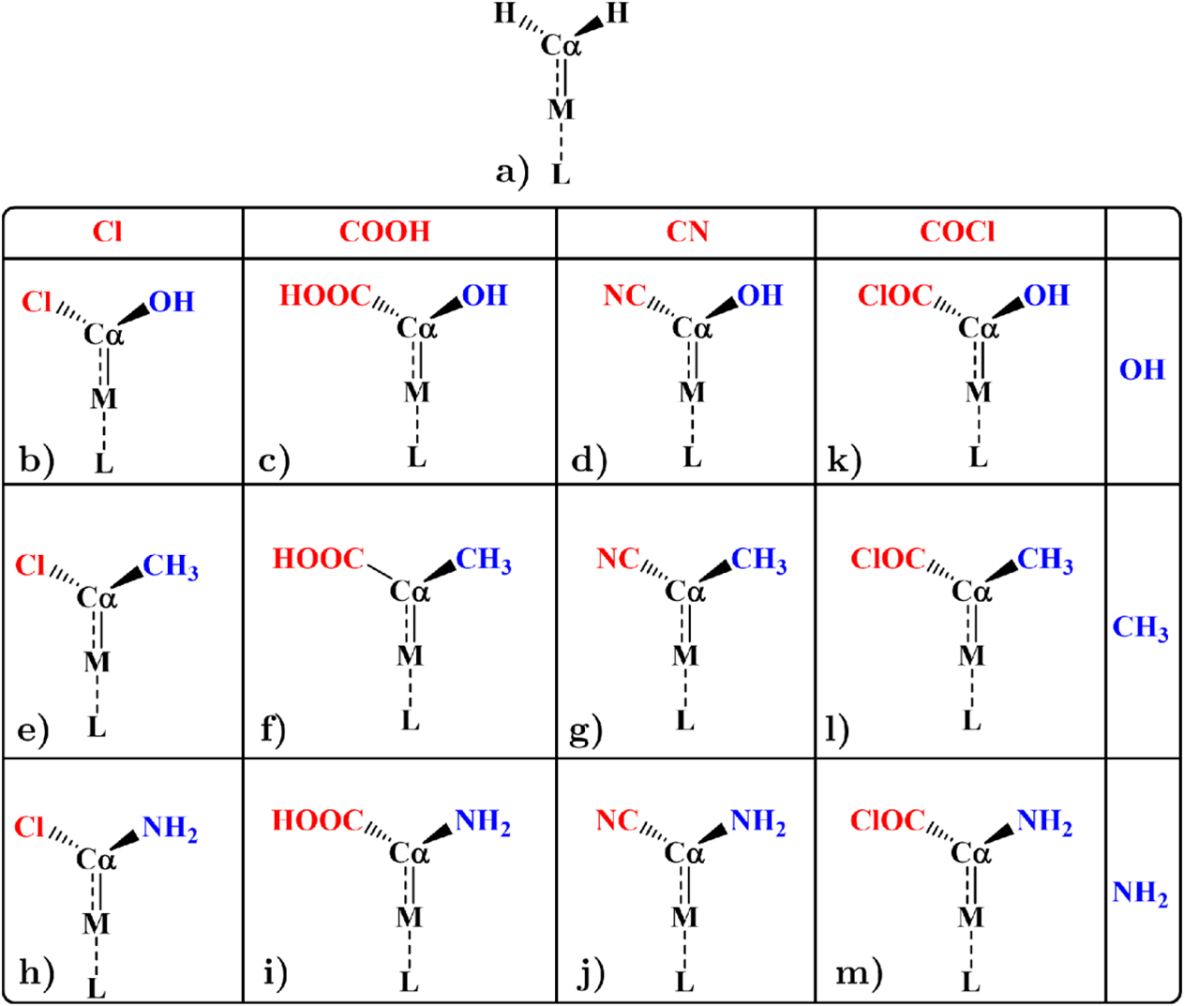
All Studied Carbenoid Conformations (**a–m**) Are Shown with the Transition Metal Represented as M (Cu, Ag, Au, Ni, Pd, or Pt)^a^

a The ligands (L) are 2,2′bipyridine for Cu, Ag, and Au, and 2,2′:6′,2″-terpyridine for Ni, Pd, and Pt. Electron-withdrawing groups (EWGs) are indicated in red, whereas electron-donating groups (EDGs) are shown in blue. The carbenoid carbon is denoted as *C*_*α*_. The COCl substituent was employed only for the Cu and Ni systems.

Methane was selected as the prototypical substrate for C–H bond activation due to its chemical simplicity, high symmetry, and inertness. As the smallest saturated hydrocarbon, methane provides a minimal and unbiased model system that isolates the intrinsic reactivity trends induced by carbenoid substitution, without introducing additional steric or electronic perturbations from more complex alkanes. Moreover, its strong and nonpolar C–H bonds make it well suited for assessing activation barriers and for evaluating substituent effects at the carbenoid center. The concerted C–H insertion pathway considered for all systems investigated in this work is schematically illustrated in Scheme 2.

**Scheme 2:**
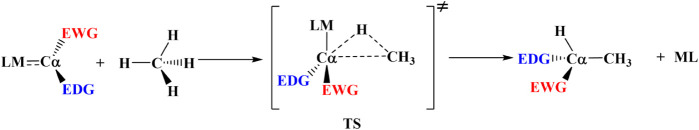
General Schematic Representation of the Concerted C–H Insertion Mechanism Mediated by Transition-Metal Carbenoids Examined in this Work

A systematic study was conducted using Density Functional Theory (DFT) at the M06-2X^47^ level, in combination with the cc-pVDZ basis set^48,49^ and the LANL2DZ quasi-relativistic pseudopotential for Cu, Ag, Au, Ni, Pd, and Pt atoms.^50–52^ The GD3 dispersion correction method was incorporated into the self-consistent field energies and gradients to account for short-range atomic repulsion interactions.^53^

To ensure the validity of all stationary points, frequency calculations were carried out using analytical second derivatives, including zero-point energy (ZPE) corrections and thermal contributions at 298 K and 1 atm. The reactants (R) exhibited no imaginary frequencies, whereas each transition state (TS) displayed a single imaginary frequency corresponding to the reaction coordinate, confirming their proper characterization. Time-dependent density functional theory (TD-DFT) calculations were performed within the singlet manifold, considering the lowest 20 excited states to sample relevant electronic transitions for each system comprehensively.^54,55^

All calculations, including energies, reaction coordinates, and structural and energetic properties of the molecular systems, were carried out using the Gaussian 16 B.01 package.^56^ Wave function-based analyses, such as those involving the Δ*f*(**r**), were performed using the GaussView 6 program,^57^ meanwhile the local electrophilicity was quantified by integrating the Dual Descriptor Δ*f*(**r**) over its sign-defined domains using the algorithm described by Tognetti et al.^38^

## 4 Results and Discussion

Describing the suggested systems before examining the reaction process is necessary to guarantee the effective activation of the C–H bond during interaction with the TM carbenoids. To create a reactivity order for substitution with EDG and EWG, ten carbenoids (**a** to **j**) considered using group 10 (Ni, Pt, and Pd) and 11 (Cu, Ag, and Au). They will be examined in terms of their geometries, inherent reactivity from CDFT, and electrical characteristics. We have included an unsubstituted system (**a**) for reference in the following analysis and **j, k,** and **l** carbenoids will not be used here.

### 4.1 Carbenoid Characterization

#### 4.1.1 Geometrical Parameters

Figure 1a presents the geometrical parameters for the group 11 metal carbenoids (TM = Cu, Ag, Au). This includes the bond lengths of TM–Cα (*d*_1_), TM–N1 (*d*_2_), and TM–N2 (*d*_3_), as well as the angles Cα–TM–N1 (∠α), N1–TM–N2 (∠β), and the dihedral angle C–N1–N2–TM (θ), which is indicative of the planarity.

**Figure 1:**
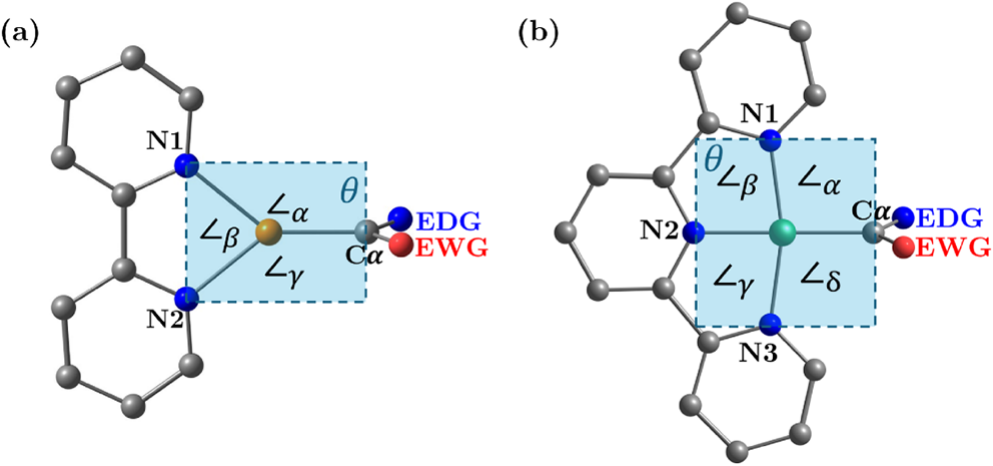
(a) Geometrical parameters for group 11 (TM = Cu, Ag, Au) and (b) group 10 (TM = Ni, Pd, Pt) carbenoids. Bond lengths, bond angles, and dihedral angle θ are indicated. Cα is the carbenoid carbon; EDG = blue, EWG = red. All values are presented in Table S1.

The bond lengths exhibit expected trends down the group. The *d*_1_ bond shows values in the ranges of 1.91–2.02 Å for Cu, 2.10–2.23 Å for Ag, and 1.87–1.97 Å for Au, reflecting the interplay between increasing metallic radius and relativistic contraction for gold. The TM–N bonds (*d*_2_ and *d*_3_) follow a similar pattern, lengthening significantly from Cu (2.07–2.11 Å) to Ag (2.33–2.38 Å), with Au exhibiting a range of 2.29–2.47 Å.

The angular parameters are definitive for the trigonal-planar structure. The ∠α is consistently the largest, with values of 124.08–141.26° for Cu, 126.19–145.12° for Ag, and 143.89–154.95° for Au. The angle ∠β is within ranges of 139.66–156.04° for Cu, 143.95–162.63° for Ag, and 136.55–146.04° for Au. The angle ∠γ consequently compressed to a range of 78.72–80.22° for Cu, 70.30–71.54° for Ag, and 68.40–70.85° for Au. The consistently small dihedral angles θ (Cu: 0.00–3.72°; Ag: 0.00–3.11°; Au: 0.00–2.19°) confirm that the carbenoid carbon and the transition metal lie in the same plane, preserving the overall trigonal-planar geometry.

In addition, the degree of angular distortion from the ideal trigonal planar geometry was quantified using the geometric descriptor τ_3_, which represents an extension of the τ_4_ index to three-coordinate systems.^58^ τ_3_ is calculated as $\tau_{3}=\frac{360-\left(\alpha +\beta \right)}{120}$ , where α and β represent the two largest valence angles. In this scale, a value of 1.00 represents a perfect trigonal planar geometry, while 0.00 indicates a trigonal pyramidal environment. The calculated τ_3_ values (Cu: 0.66–0.67; Ag: 0.59–0.60; Au: 0.57–0.59) confirm a significant and systematic distortion across the series, with the Ag and Au complexes exhibiting the greatest deviations from planarity. In this sense, the *d* ^10^ metals of group 11 present a trigonal-planar coordination sphere, characterized by two wide angles and one acute angle at the transition metal center, alongside the minimal dihedral angles confirming the planarity of these structures.

Figure 1b presents the geometrical parameters for the group 10 carbenoids (TM = Ni, Pd, Pt). This includes the bond lengths of TM–Cα (d_1_), TM–N1 (d_2_), TM–N2 (d_3_), and TM–N3 (d_4_), as well as the angles Cα–TM–N1 (∠α), N1–TM–N2 (∠β), N2–TM–N3 (∠γ), Cα–TM–N3 (∠δ), and the dihedral angle Cα–N1–N2–N3 (θ), all of which are located in the plane of the figure.

These results indicate that all carbenoids present a square-planar geometry, arising from the *d* ^8^ electronic configuration of the transition metal centers. The Ni carbenoid exhibits bond distances within the ranges of *d*_1_ = 1.93–1.99 Å, *d*_2_ = 1.97–1.98 Å, *d*_3_ = 1.88–1.89 Å, and *d*_4_ = 1.97–1.98 Å. Its angular parameters, with ∠α = 96.70–97.94°, ∠β = 82.12–82.51°, ∠γ = 82.12–82.51°, and ∠δ = 97.48–98.77°, along with a minimal dihedral angle θ ranging from 0.01° to 4.57°, confirm a geometry closest to the ideal square-plane.

In contrast, the Pd and Pt carbenoids exhibit a systematic distortion. Their TM–N2 and TM–N3 bonds (*d*_3_ and *d*_4_) are longer, with Pd ranging from 2.11–2.12 Å and Pt from 2.08–2.09 Å, and their ∠α and ∠δ expand to ranges of 99.74–101.66° and 100.03–102.00° for Pd, and 99.91–102.12° and 100.25–102.09° for Pt, while the angles ∠β and ∠γ are further compressed to ranges of 78.34–79.36° for Pd and 78.63–79.52° for Pt. Despite this distortion, the small dihedral angles θ (Pd: 0.00–4.51°; Pt: 0.00–3.78°) for all carbenoids maintain the essential planar geometry. The deviation from ideal square-planar geometry was quantified by the geometry index τ_4_, calculated using the formula $\tau_{4}=\frac{360-\left(\alpha +\beta \right)}{360-2\left(109.5\right)}$ .^58^ In this scale, τ_4_ = 0 represents a perfect square-planar geometry, whereas τ_4_ = 0 corresponds to a perfect tetrahedral environment, while α and β are the largest angles. The computed average τ_4_ values (Ni: 0.11; Pd: 0.16; Pt: 0.16) are consistently low, quantitatively confirming that all Group 10 complexes adopt a square-planar coordination with only minor distortions.

These results confirmed that the planarity of the carbenoids and provides a well-defined, sterically unhindered face for the approach of the C–H bond substrate. Consequently, the reaction is predisposed to proceed via a selective, direct insertion pathway at the electrophilic *C*_α_, facilitating regioselective C–H bond activation.

#### 4.1.2 Activation Barriers

After exploring the structural properties of the carbenoids, we evaluated their potential for C–H activation and insertion reactions for metals of groups 11 and 10. In this study, C–H activation was modeled as a concerted pathway that proceeds through the formation of a σ complex, where *Cα* interacts directly with the C–H bond. To this end, we calculated the activation Gibbs free energies (Δ*G* ^‡^) using methane (me) as a substrate to represent the activation mechanism of the C–H bond. The activation energy was defined as

$$\Delta G^{‡}=E_{\mathrm{TS}}-E_{R}$$

$$E_{R}=E_{\mathrm{me}}+E_{\mathrm{Carbenoid}}$$

$$E_{\mathrm{TS}}=E_{\mathrm{Carbenoid‐me}}$$

where Δ*G* is the activation energy, defined as the energy difference between the transition state (TS) and the reactant state (R). The reactant energy, *E*_R_, corresponds to the sum of the isolated energies of methane (*E*_me_) and the carbenoid fragment (*E*_carbenoid_); this reference was chosen to mitigate the geometric dependence of Basis Set Superposition Error (BSSE) that would occur if bound reactant complexes were used.^59^ At the same time, *E*_TS_ refers to the energy of the carbenoid–methane system interacting in the optimized geometry of the TS.

The Δ*G* ^‡^ values were computed for a representative set of carbenoids using Ag, Cu, Au, Pd, Ni, and Pt, providing a broader view across the periodic table. The resulting activation barriers display consistent trends, as shown in Figure 2a, b, with the following order: Ag > Cu > Au and Pd > Ni > Pt.

**Figure 2:**
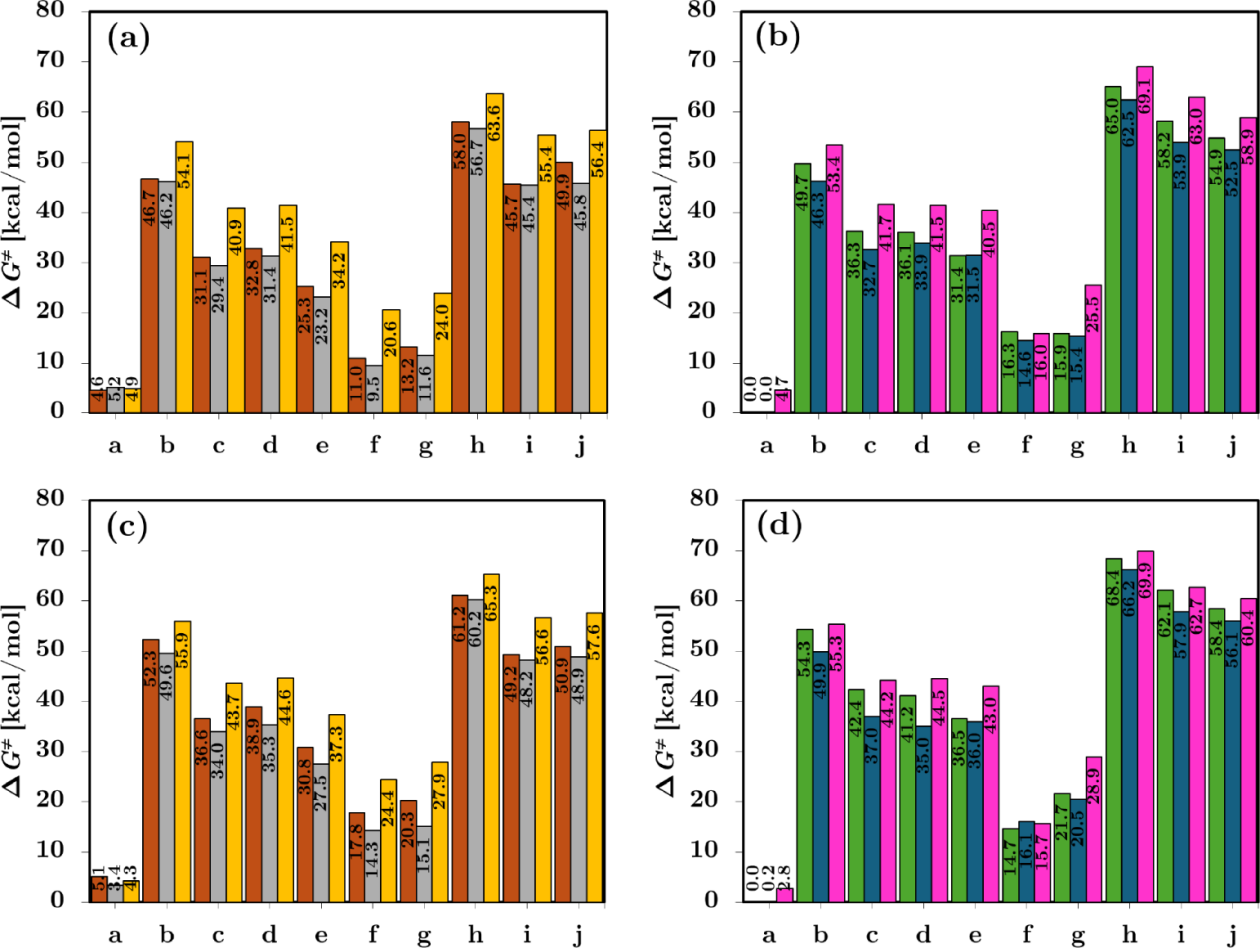
Activation Gibbs free energy barriers (Δ*G* ^‡^) in kcal/mol, comparing different functionals and metals. (a) and (b) show results computed with M06-2X for Cu, Ag, Au, and Ni, Pd, Pt metals, respectively, while (c) and (d) display analogous benchmarks using the ωB97XD functional. Values are presented in Table S2.

In order to benchmark these results, we compared the Δ*G* ^‡^ obtained with M06-2X to those computed using the ωB97XD functional,^60^ which is widely employed in studies of organometallic reactivity involving second- and third-row transition metals.^61,62^ As shown in Figure 2c, d, the two functionals yield consistent qualitative trends across the metal series. In particular, both methods reproduce the ordering Ag > Cu > Au for group-11 metals and Pd > Ni > Pt for group-10 metals over the majority of carbenoids. For the low-barrier systems (f and g), M06-2X systematically underestimates the Δ*G* ^‡^ relative to ωB97XD by approximately 4–7 kcal/mol, although the qualitative metal dependence is preserved. For the high-barrier carbenoid h, ωB97XD predicts Δ*G* ^‡^ of 65.4 and 69.9 kcal/mol for Au and Pt, respectively, in close agreement with the corresponding M06-2X values (63.6 and 69.1 kcal/mol), further supporting the robustness of the observed trends.

According to transition state theory, as formalized by the Eyring equation, the reaction rate constant depends exponentially on the Δ*G* ^‡^, such that changes of only a few kcal/mol in Δ*G* ^‡^ can translate into orders-of-magnitude differences in reaction times.^63^ At 298 K, an Δ*G* ^‡^ of ∼10 kcal/mol corresponds to a reaction occurring on the subsecond time scale, whereas barriers of ∼20 and ∼30 kcal/mol are associated with reactions occurring over hours and days, respectively. In this context, reactions with Δ*G* ^‡^ ≤ 1–5 kcal/mol are effectively barrierless on the experimental time scale.

Based on the computed Δ*G* ^‡^ values, the carbenoids were categorized into three groups: high, medium, and low activation barrier processes. High activation barriers (typically >40 kcal/mol) were observed for carbenoids **b** and **h**–**j**; medium barriers (30–40 kcal/mol) for **c**, **d**, and **e**; and low barriers (<20 kcal/mol) for **f** and **g**. Notably, carbenoid **a**, with computed Δ*G* ^‡^ values close to 0 kcal/mol across all metals, exhibited a barrierless reaction. Overall, the agreement between methods demonstrates that the original computational approach (M06-2X) reliably captures the Δ*G* ^‡^ trends across a diverse set of transition-metal carbenoids.

In addition, the frontier orbital energies underlying the descriptor analysis were examined by comparing HOMO and LUMO energies and the resulting gaps obtained with M06-2X and ωB97XD, confirming that the relative orbital-energy trends are preserved across functionals, with correlations of 92% for Cu and 99% for Ni systems based on the HOMO–LUMO gaps (see Supporting Information, Figure S1; Table S3).

Our results indicate that Ni carbenoids are good candidates to be used for C–H activation and insertion reactions presenting reactivity and energy patterns comparable to their widely used noble counterparts, most notably with its analogous counterpart Cu. To further explore these similarities, we retrieved the proposed structures of the reported Cu carbenoids and computed their corresponding activation barriers with our selected methodology. This comparative analysis enables a direct assessment of whether the energy trends and mechanistic features observed for Ni are conserved in Cu. Having validated the reliability of the M06-2X functional for describing activation processes, we applied it to both Ni and Cu carbenoids as shown in Figure 3.

**Figure 3:**
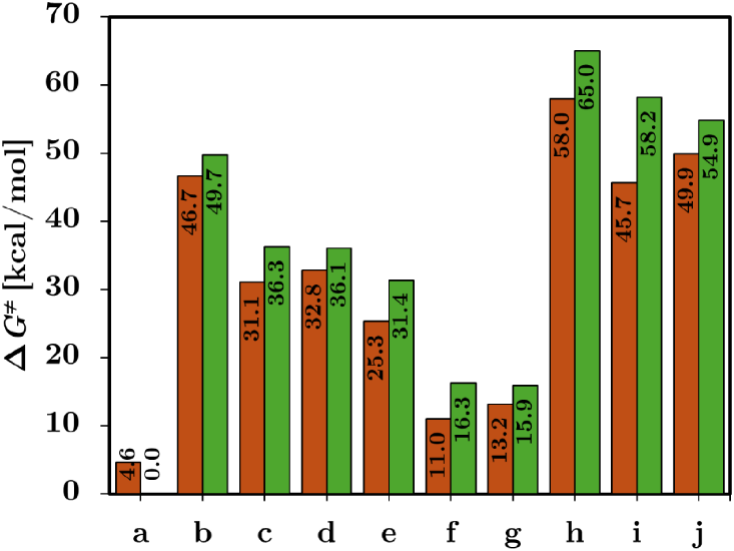
Activation energy barriers (Δ*G* ^‡^) in kcal/mol for Cu (orange) and Ni (green) carbenoids. Values are in Table S4.

Both Cu and Ni carbenoids span a wide energetic range, from barrierless processes (**a**) to highly activated reaction mechanisms (**h**). Despite this variability, Cu generally exhibits lower or comparable Δ*G* ^‡^ values relative to Ni. Carbenoids **c** to **e** display intermediate activation barriers, with Δ*G* ^‡^ values from 25.3–32.8 kcal/mol for Cu and 31.4–36.3 kcal/mol for Ni. Carbenoids **f** and **g** yield low barriers for both metals (Cu: 11.01 and 13.21 kcal/mol; Ni: 16.3 and 15.9 kcal/mol), indicative of minimal TS energy requirements with no control over the reaction. By contrast, Carbenoids **h** to **j** result in high activation barriers, exceeding 45 kcal/mol in all cases. Specifically, **h** yields Δ*G* ^‡^ values of 58.0 kcal/mol for Cu and 65.0 kcal/mol for Ni, hindering the reaction mechanisms to activate the C–H bond. Nevertheless, these results indicate that the activation barriers for Cu are lower than those for Ni, suggesting a kinetic preference for Cu-based carbenoids.

This is attributed to a Natural Bond Orbital (NBO) analysis,^64^ which reveals similar donor–acceptor interactions for both metals. Specifically, the *N*_*i*_–*C*_*α*_ bond is stabilized by *sp* ^2^(C) → *sd*(Ni) σ-donation and *d*(Ni) → *p*(C) π-backdonation. This bonding pattern directly resembles the *sp* ^2^(C) → *d*(Cu) and *d*(Cu) *p*(C) interactions obtained for the Cu analogues. Overall, regarding the reactivity associated with different substituent combinations, Ni displays the same qualitative trends observed for Cu.

### 4.2 Comparison between Ni and Cu Carbenoids

To deepen the understanding of the observed reactivity trends between Ni and Cu carbenoids, it is essential to complement energetic analyses with conceptual descriptors that capture the underlying electronic factors. While activation barriers provide a kinetic perspective, they do not always offer mechanistic insight into the local electronic properties that govern reactivity. Accordingly, we extended our analysis by examining the reactivity patterns using the Dual Descriptor, which allows for the identification of electrophilic and nucleophilic regions.

#### 4.2.1 Relation between Activation Barriers and the Dual Descriptor

Reactivity patterns were examined using the Dual Descriptor [Δ*f*(**r**)], as shown in Figure 4, revealing a well-localized electrophilic regions Δ*f* > 0, in red) centered on the carbenoid Cα for all systems except system **h**, which did not exhibit the electrophilic region at the Cα. In order to enable a quantitative comparison, domain-condensed Dual Descriptors [Δ*f*(*D*_*i*_)] was computed by integrating Δ*f*(**r**) over regions centered on the Cα using a grid base algorithm. These values showed moderate correlation with Δ*G* ^‡^, with *R* ^2^ of 58% for Cu and 64% for Ni systems (Figure 4a, b; orange).

**Figure 4:**
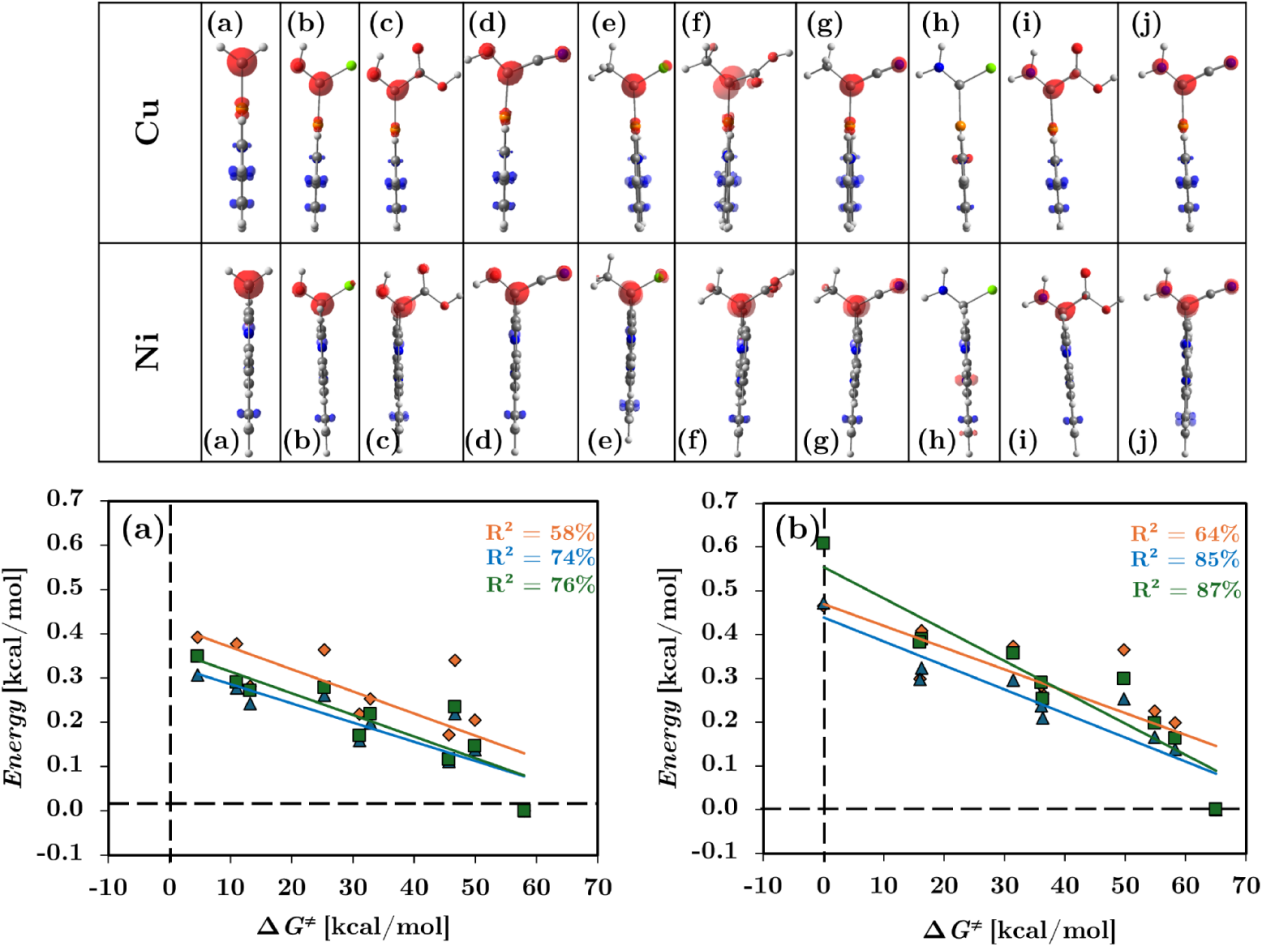
Dual Descriptor Δ*f*(**r**) for all carbenoids. Red surfaces indicate electrophilic regions, while blue surfaces correspond to nucleophilic regions. An isosurface value of 0.015 was used for all systems to clearly define the domains. (a) Correlation values for the Cu carbenoids and (b) Ni carbenoids. Orange represents Δ*f*(*Cα*), while blue and green correspond to the GCDDs scaled by S and P, respectively. Plot values are presented in Table S5.

Further refinement was achieved through a Grand Canonical ensemble. The Grand Canonical ensemble is particularly useful for comparing systems with different numbers of electrons because it allows for particle exchange with a reservoir, ensuring that the electronic chemical potential (rather than the total electron count) remains constant across systems. This enables a consistent comparison of reactivity descriptors even when the systems under study vary in electron count. In this sense, the condensed Dual Descriptor was scaled using two different Grand Canonical ensemble approaches, the global softness (*S*) and the recently proposed ensemble transitions the hypersoftness (*P*) from a composite descriptor to a basic descriptor demonstrating improved accuracy. For practicality, we will name these approaches SGCDD and PGCDD, respectively.

By incorporating the systems electronic response through the SGCDD ensemble, the correlation with activation barriers improved significantly, with *R* ^2^ rising to 74% for Cu and 85% for Ni systems as shown in Figure 4a, b in blue with an overall increase of 16% and 21%, respectively. When obtaining the PGCDD a slight improved correlation values to 76% for Cu and 87% for Ni systems were obtained as shown in Figure 4a, b in green, increasing by 18% and 23%, respectively. Based on these results the following analysis in this research will be carried out using the improved PGCDD approach. Given that system **h** did not display the expected electrophilic region at *Cα*, additional strategies were explored to characterize its reactivity.

#### 4.2.2 Electronic Structure and Reactivity Corrections for Carbenoid **h**

As mentioned previously, the carbenoid **h** did not exhibit a well-defined Δ*f*(*Cα*) value. To address this, we explored alternative computational approaches to correctly describe the electrophilic region. When unoccupied orbitals are involved in defining a reactive site, careful treatment of their contribution is essential to accurately capture local reactivity.^65^ Using appropriate methodologies ensures that the calculated descriptors reliably reflect the electronic structure.

In system **h**, considering higher virtual orbitals (LUMO+n) better represents the accessible unoccupied states. To verify this, we compared the finite-difference approximation (FDA) with the frontier molecular orbital approximation including orbital relaxation (FMOA),^66^ as shown in Figure S2. This refinement is necessary because the conventional orbital-based finite-difference approximation to Δ*f*(**r**), where Δ*f*(*r*) ≈ *f* ^+^(*r*) – *f* ^–^(*r*) is approximated as the density difference between the LUMO and HOMO, may fail when the LUMO does not contribute significantly to the electrophilic reactivity at the site of interest. Both approaches yielded consistent results, confirming that orbital relaxation effects do not significantly affect the local reactivity in this system.

Based on these observations, the FMOA approach and the exploration of LUMO + *n* orbitals were applied. For the Cu system, the LUMO + 1 orbital was employed, whereas for the Ni system, the LUMO + 2 orbital correctly localized the electrophilic region at *Cα*. Using these orbitals, Δ*f*(*r*) was recalculated for carbenoid **h**. These modified orbital selections enabled the recovery of a Δ*f*(*r*) at *Cα*, allowing the direct computation of meaningful domain-integrated values for system **h**, as discussed in the SI (see Figure S3 and Table S6). While this approach yields moderate-to-high correlations with the PGCDD, it also leads to an overestimation of the integrated domain value at *Cα*.

As an alternative strategy, we employed the Specific State Dual Descriptor (SSDD) framework. Unlike the ground-state Δ*f*(**r**), the SSDD is constructed from electron density differences between selected excited states and the ground state, thereby enabling a direct connection between reactivity patterns and specific electronic transitions. Within this formalism, Δ*f* is defined as the electron density difference between an excited state and the ground state, capturing reactivity features that emerge from accessible excited-state configurations rather than solely from frontier orbital interactions. This approach is particularly appropriate for system **h**, where the ground-state LUMO does not localize at the reactive *C*_α_ and therefore fails to describe the electrophilic character governing C–H activation.

Time-dependent density functional theory (TD-DFT) calculations were performed to identify the relevant excited states for each system. For the Cu series, the relevant excitations corresponded to transitions from the HOMO to the LUMO + 1, associated with excited states 12 and 16, respectively. In the Ni systems, the relevant transitions involved the HOMO to LUMO + 2 excitations and were identified as excited states 10 and 14. The characteristics of these transitions, including their main electron excitations and TD-DFT expansion coefficients, are summarized in Table 1.

**Table 1:** Summary of Critical Electron Excitations Selected for SSDD Construction

TM	State	Electron Excitations	Expansion Coefficient
Cu	12	HOMO → LUMO + 1	0.21283
Cu	16	HOMO → LUMO + 1	0.66825
Ni	10	HOMO → LUMO + 2	0.67804
Ni	14	HOMO → LUMO + 2	0.17460

The SSDD spatial distributions derived from these transitions are illustrated in Figure 5a, which highlights the corresponding excitations for Cu and Ni systems. This excited state based approach successfully restores an electrophilic region centered on the carbenoid *C*_α_ in system **h**, which is in agreement with the LUMO + *n* orbital-based correction discussed earlier. Moreover, it offers a more rigorous and physically grounded justification for the observed reactivity patterns in cases where the ground-state Δ*f*(**r**) is missing.

**Figure 5:**
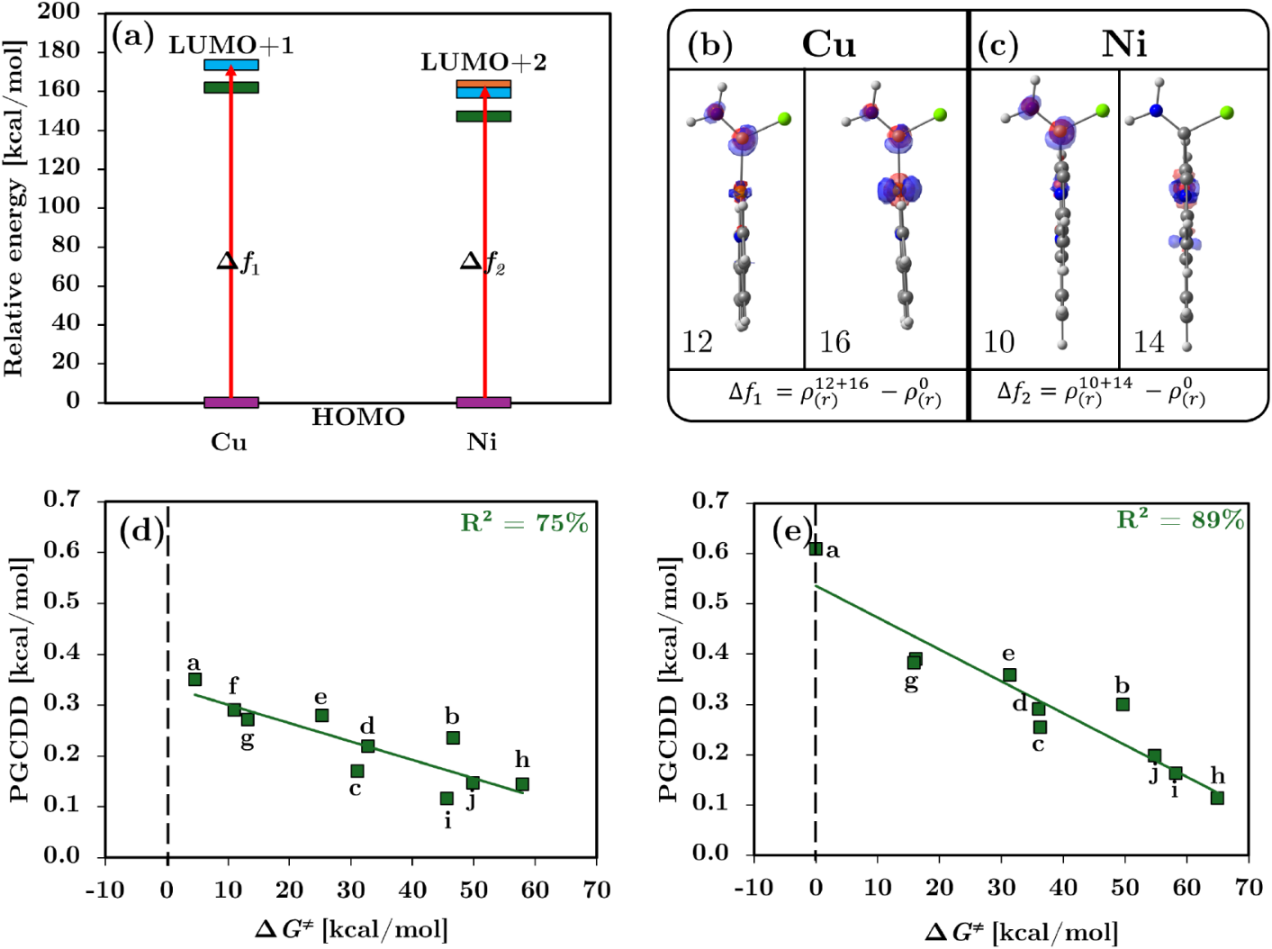
(a) Corresponding electron excitation for Cu and Ni carbenoids. (b, c) SSDD for system **h**. Updated correlations for (d) Cu and (e) Ni carbenoids. Plot values are presented in Table S7.

Following the construction of the SSDD for system **h**, we reevaluated the condensed GCDD values integrated over the Δ*f*(*C*_α_). As shown in Figure 5b, c, the SSDD successfully recovers the electrophilic region localized at the reactive *C*_α_ for system **h**, correcting the deficiencies observed with the ground-state descriptor.

Notably, the correlation between the PGCDD and the activation barriers improves when compared to the LUMO+n approach, with coefficients of 75% for the Cu systems and 89% for the Ni systems, as shown in Figure 5d, e. Thus, the SSDD framework provides a physically grounded correction for anomalous cases such as system **h** without compromising the strong predictive performance initially established using the unmodified PGCDD. Overall, the consistent performance of the Δ*f*(**r**) domain methodology across both Cu and Ni systems establishes a unified predictive framework for C–H activation reactivity.

#### 4.2.3 Introducing −COCl as an EWG Substitute

By introducing a substituent that acts as a predictable, moderately strong EWG without the conflicting +M effects of −Cl, the carbonyl chloride group (−COCl) was selected for this purpose to validate the improved correlation. Systems **k** (OH/COCl), **l** (CH_3_/COCl), and **m** (NH_2_/COCl) were designed to provide a broader perspective. All calculations were performed using the same computational methodology.

Upon introduction of the −COCl substituent, a clear modulation of the activation barriers was observed across the examined systems (Figure 6). The OH/COCl derivative (**k**) displayed moderate barriers for both Cu and Ni (29.0 and 30.5 kcal/mol, respectively), consistent with the electron-withdrawing character of the −COCl group. In contrast, the CH_3_/COCl system (**l**) exhibited one of the lowest barriers in the series (14.2 kcal/mol for Cu and 13.0 kcal/mol for Ni), indicating that, when combined with a weakly donating alkyl group, −COCl efficiently stabilizes the transition state. Conversely, the NH_2_/COCl analogue (**m**) presented significantly higher activation barriers (43.4 kcal/mol for Cu and 53.8 kcal/mol for Ni). Most importantly, the strong −I character of −COCl allowed us to obtain a better distribution of activation barriers compared to the −Cl substituent, for which the activation energies were higher. These results confirm that −COCl acts as a predictable, moderately strong EW probe, enhancing the substituent–barrier correlation without introducing the confounding resonance effects characteristic of −Cl. The consistently higher barriers observed for Ni relative to Cu further highlight the robustness of the substituent-dependent trend across both metal centers.

**Figure 6:**
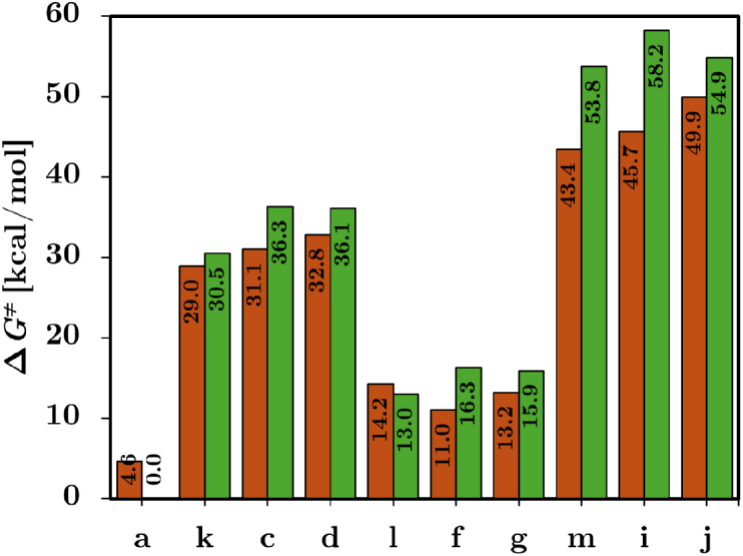
Updated activation energy barriers (Δ*G* ^‡^) for Cu (orange) and Ni (green) carbenoids. Values are reported in Table S8.

Analysis of the Dual Descriptor for these systems confirmed the electrophilic nature of the carbenoid center, with the electrophilic region localized on the Cα atom as shown in Figure 7a, b. This observation verifies that −COCl behaves as an electron-withdrawing group as intended, without requiring additional corrective procedures such as the SSDD approach. Furthermore, Figure 7c, d shows that inclusion of the −COCl systems yields a high correlation between the PGCDD descriptor and the activation barriers, with coefficients of 86% for the Cu systems and 88% for the Ni systems. This outcome underscores the reliability of PGCDD in capturing the electronic control exerted by substituents over the reaction barrier when their electronic character remains internally consistent.

**Figure 7:**
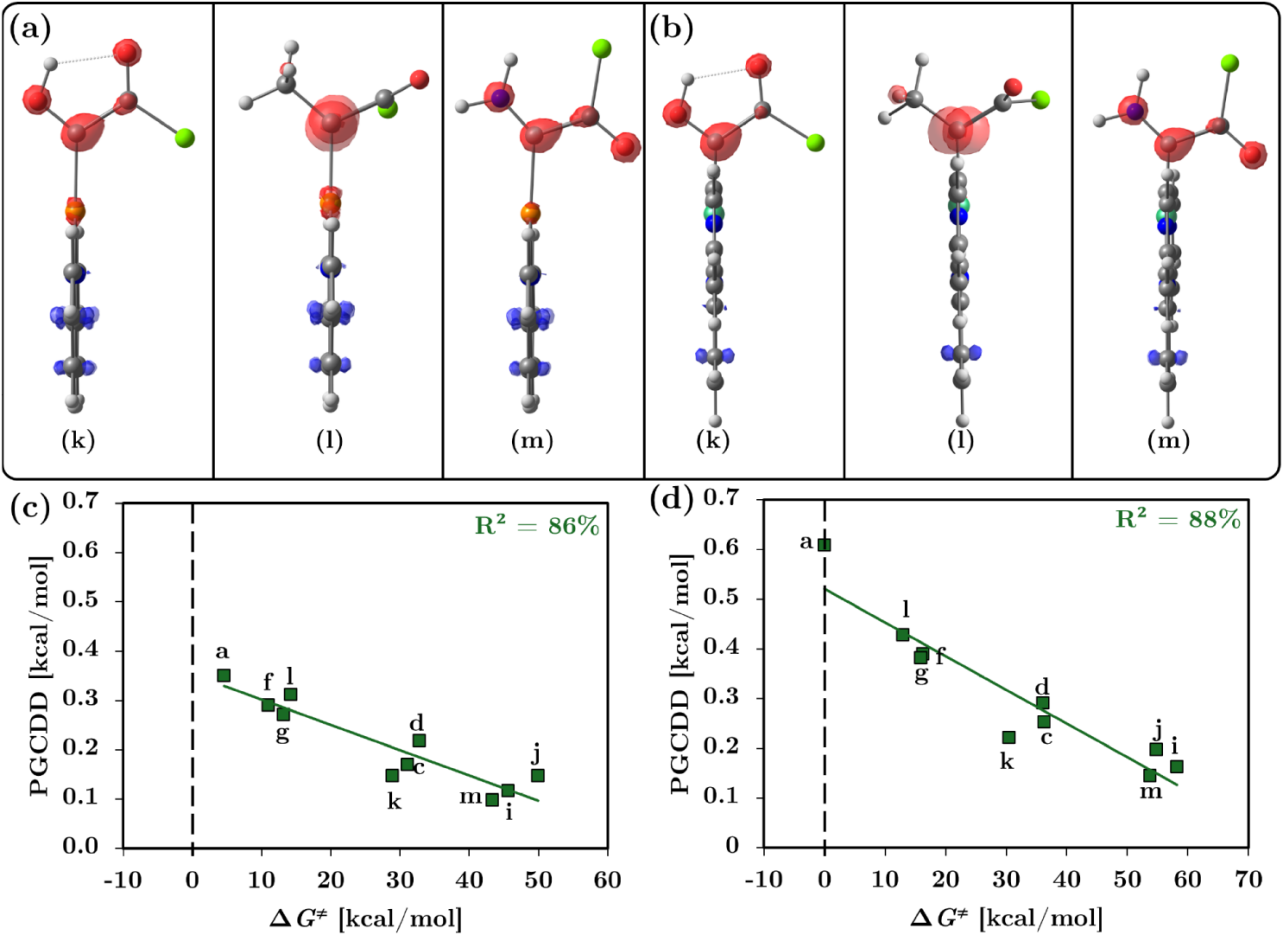
(a, b) Dual Descriptor showing the electrophilic region (red) localized on the Cα atom. (c, d) Correlation between PGCDD and Δ*G* ^‡^, utilizing the new systems with the −COCl substituent (k, l, m). Plot values are presented in Table S9.

In summary, the integration of conceptual DFT descriptors, particularly the Dual Descriptor and its Grand Canonical extensions, provides a consistent and quantitative framework for interpreting the reactivity of Ni and Cu carbenoids toward C–H activation. By capturing local electronic effects at the carbenoid center and correlating them with activation barriers, we demonstrate that both ground-state and excited-state electronic structures offer valuable insights into reactivity trends. These findings validate the predictive utility of domain-condensed reactivity indices and underscore their potential for guiding the rational design of carbenoid-based catalysts. The insights gained from this comparative analysis reinforce the broader applicability of descriptor-based approaches in bond activation.

## 5 Conclusions

This work presents a comprehensive theoretical investigation of C–H bond activation mediated by transition metal carbenoids, using methane as a model substrate and a range of electron–donating and electron–withdrawing substituents. The results demonstrate that modulation of carbenoid electrophilicity through substituent effects determines the activation barriers. Although Ni carbenoids generally display higher barriers than their Cu counterparts, both metals exhibit analogous reactivity patterns, underscoring the transferability of descriptor analyses across first–row transition metals.

Within the framework of CDFT, the Dual Descriptor and its Grand Canonical extensions (SGCDD and PGCDD) effectively capture the relationship between local electronic structure and reactivity. Among these, the PGCDD descriptor emerged as the most robust predictive parameter for both Cu and Ni systems. In cases where ground state descriptors failed particularly for -Cl and NH_2_ refinements involving higher virtual orbitals and the Specific State Dual Descriptor (SSDD) successfully restored accurate localization of electrophilic regions and improved the overall correlations with activation barriers.

Furthermore, substitution of −Cl by −COCl as a representative EWG eliminated resonance ambiguities and enhanced the reliability of the descriptor correlations, increasing the *R* ^2^ values. This confirms that consistent electronic character of substituents is essential for robust descriptor performance.

Overall, this study establishes a unified CDFT framework for interpreting and predicting C–H insertion reactivity in transition metal carbenoids. The methodology offers a transferable and quantitative foundation for the rational design of first-row transition metal catalysts, thereby advancing the predictive application of conceptual DFT in carbenoid and C–H activation chemistry.

## Supplementary Material



## Notes

HOMO–LUMO gap comparisons at the M062X and ωB97XD levels for Cu and Ni carbenoid systems (Figure S1); comparison between FDA and FMOA approaches for system **h** (Figure S2); electrophilic region for system **h** using higher unoccupied molecular orbitals (Figure S3); geometrical parameters for all studied carbenoids (Table S1); benchmark of activation energies using M06–2X and ωB97XD functionals (Table S2); HOMO and LUMO energies and corresponding HOMO–LUMO gaps for Cu and Ni carbenoid systems (Table S3); activation energies for Cu and Ni carbenoid reactions (Tables S4 and S9); calculated activation energies and quantified Dual Descriptor domains for copper and nickel systems (Table S5); calculated activation energies and the Grand Canonical Dual Descriptor for Cu and Ni systems using higher unoccupied molecular orbitals (Table S6); State-Specific Dual Descriptor analysis (Table S7); calculated activation energies and the Grand Canonical Dual Descriptor for Cu and Ni systems including additional substituents (Table S8); and Cartesian coordinates for all studied structures (Table S10) (PDF)
