# Supplementary material for: Nickel and Copper in C–H Activation and Carbenoid Chemistry: A Descriptor-Based Comparative Analysis of Transition Metals
Source: J Phys Chem A. 2026 Jan 27;130(5):1078–89. doi: 10.1021/acs.jpca.5c07321 (PMC12884515; doi:10.1021/acs.jpca.5c07321)
Supplement: Supplementary file 1 [file jp5c07321_si_001.pdf]

Nickel and Copper in C–H Activation and Carbenoid Chemistry: A Descriptor-Based Comparative Analysis of Transition Metals

Sasha Gazzari-Jara,[†] Olivier Aroule,[‡] Guillaume Hoffmann,[‡] Henry Chermette,[‡] Christophe Morell,[*,‡] and Bárbara Herrera[*,†]

[†]QTC, Departamento de Química Física, Pontificia Universidad Católica de Chile, Avenida Vicuña Mackenna 4860, Macul, Santiago, Chile

[‡]Université de Lyon, Institut des Sciences Analytiques, UMR 5280, CNRS, Université Lyon 1 - 5, rue de la Doua, F-69100 Villeurbanne, France;

E-mail: christophe.morell@univ-lyon1.fr; bherrera@uc.cl

Contents in this document

Figure S1: HOMO–LUMO gap comparison at the M06-2X and ωB97XD levels for Cu and Ni carbenoid systems.

Figure S2: Comparison between FDA and FMOA approaches for system **h.**

Figure S3: Electrophilic region for system **h** using higher unoccupied orbitals.

Table S1: Geometrical parameters for the studied carbenoids.

Table S2: Benchmark of activation energies for all carbenoid reactions using two different functionals.

Table S3: HOMO and LUMO energies and corresponding HOMO-LUMO gaps for Cu and Ni carbenoid systems.

Table S4: Activation energies for Cu and Ni carbenoid reactions.

Table S5: Calculated activation energies and quantified Dual Descriptor domains for copper and nickel systems.

Table S6: Calculated activation energies and the Grand Canonical Dual Descriptor for Cu and Ni systems using higher unoccupied molecular orbitals**.**

Table S7: Calculated activation energies and the Grand Canonical Dual Descriptor for Cu and Ni systems using the State Specific Dual Descriptor approach.

Table S8: Calculated activation energies and the Grand Canonical Dual Descriptor for Cu and Ni systems including additional substituents.

Table S9: Activation energies for Cu and Ni carbenoid reactions including additional substituents.

Table S10: Cartesian coordinates for all studied structures.

Table S1. Geometrical parameters for the studied carbenoids. Bond distances (*d*) are in Å.

M	*Systems*	d_1	d_2	d_3	∠*α*	∠*β*	∠*γ*	*θ*
Cu	**a(H/H)**	1.91	2.07	2.07	139.90	139.88	80.22	0.00
	**b(OH/Cl)**	2.00	2.10	2.10	140.16	140.21	79.63	1.01
	**c(OH/COOH)**	1.99	2.10	2.10	140.32	140.32	79.36	0.71
	**d(OH/CN)**	2.00	2.09	2.09	139.95	139.97	79.96	3.70
	**e(CH_3/Cl)**	1.98	2.10	2.10	139.31	141.33	79.35	1.06
	**f(CH_3/COOH)**	1.96	2.10	2.07	124.08	156.04	79.88	0.27
	**g(CH_3/CN)**	1.96	2.08	2.08	138.81	141.14	80.03	1.26
	**h(NH_2/Cl)**	2.01	2.11	2.11	141.26	139.66	78.99	3.21
	**i(NH_2/COOH)**	2.01	2.11	2.11	140.63	140.62	78.72	1.91
	**j(NH_2/CN)**	2.02	2.10	2.10	140.29	140.30	79.29	3.72
Ag	**a(H/H)**	2.10	2.33	2.33	144.24	144.23	71.54	0.00
	**b(OH/Cl)**	2.23	2.36	2.36	144.32	144.57	71.10	1.15
	**c(OH/COOH)**	2.20	2.36	2.36	144.54	144.64	70.81	0.44
	**d(OH/CN)**	2.22	2.35	2.35	143.98	144.51	71.44	2.99
	**e(CH_3/Cl)**	2.20	2.36	2.36	144.54	144.66	70.80	0.64
	**f(CH_3/COOH)**	2.18	2.38	2.33	126.19	162.63	71.17	0.10
	**g(CH_3/CN)**	2.18	2.35	2.34	143.13	145.54	71.32	0.86
	**h(NH_2/Cl)**	2.23	2.37	2.37	145.12	144.21	70.61	2.91
	**i(NH_2/COOH)**	2.21	2.38	2.37	144.52	145.16	70.30	1.48
	**j(NH_2/CN)**	2.23	2.36	2.37	145.10	143.95	70.87	3.11
Au	**a(H/H)**	1.87	2.29	2.29	144.58	144.57	70.85	0.00
	**b(OH/Cl)**	1.94	2.34	2.34	145.21	145.25	69.54	0.03
	**c(OH/COOH)**	1.93	2.34	2.34	145.30	145.30	69.40	0.03
	**d(OH/CN)**	1.93	2.33	2.33	144.97	144.97	70.02	2.14
	**e(CH_3/Cl)**	1.92	2.33	2.33	144.90	145.49	69.60	0.47
	**f(CH_3/COOH)**	1.90	2.32	2.31	143.89	146.04	70.07	0.06
	**g(CH_3/CN)**	1.91	2.30	2.30	144.73	144.68	70.58	0.04
	**h(NH_2/Cl)**	1.97	2.29	2.47	154.95	136.55	68.47	1.50
	**i(NH_2/COOH)**	1.96	2.38	2.38	145.79	145.79	68.40	1.53
	**j(NH_2/CN)**	1.96	2.36	2.36	145.46	145.45	69.05	2.19

M	*Systems*	d_1	d_2	d_3	d_4	∠*α*	∠*β*	∠*γ*	∠*δ*	*θ*
Ni	**a(H/H)**	1.93	1.97	1.88	1.97	97.6	82.4	82.4	97.6	0.01
	**b(OH/Cl)**	1.99	1.97	1.88	1.97	97.6	82.4	82.4	97.7	1.20
	**c(OH/COOH)**	1.97	1.98	1.89	1.98	96.9	82.2	82.2	98.8	3.53
	**d(OH/CN)**	1.99	1.97	1.88	1.97	97.5	82.5	82.5	97.5	0.28
	**e(CH_3/Cl)**	1.96	1.98	1.89	1.98	97.1	82.2	82.2	98.5	1.29
	**f(CH_3/COOH)**	1.97	1.98	1.89	1.98	97.9	82.2	82.2	97.8	4.57
	**g(CH_3/CN)**	1.97	1.98	1.88	1.97	96.7	82.3	82.3	98.8	2.65
	**h(NH_2/Cl)**	1.98	1.98	1.89	1.98	97.7	82.2	82.2	97.7	0.17
	**i(NH_2/COOH)**	1.98	1.98	1.89	1.98	97.9	82.1	82.1	97.9	0.32
	**j(NH_2/CN)**	1.99	1.98	1.88	1.98	97.7	82.3	82.3	97.7	1.19
Pd	**a(H/H)**	1.88	2.05	2.12	2.12	101.7	78.3	78.3	101.7	0.00
	**b(OH/Cl)**	2.02	2.01	2.12	2.11	101.6	79.3	79.3	100.0	2.41
	**c(OH/COOH)**	1.99	2.02	2.12	2.11	101.6	79.1	79.1	100.4	3.21
	**d(OH/CN)**	2.01	2.01	2.11	2.11	100.3	79.4	79.4	100.9	0.49
	**e(CH_3/Cl)**	1.96	2.03	2.12	2.12	100.4	78.8	78.8	102.0	1.10
	**f(CH_3/COOH)**	1.95	2.03	2.11	2.12	100.7	78.8	78.8	101.8	4.51
	**g(CH_3/CN)**	1.96	2.03	2.12	2.12	100.7	78.9	78.9	101.6	1.44
	**h(NH_2/Cl)**	2.02	2.01	2.11	2.11	101.1	79.3	79.3	100.3	0.17

	**i(NH_2/COOH)**	2.01	2.02	2.11	2.12	100.3	79.1	79.1	101.5	0.28
	**j(NH_2/CN)**	2.04	2.01	2.11	2.11	99.7	79.3	79.3	101.6	0.64
Pt	**a(H/H)**	1.87	2.04	2.09	2.09	101.0	79.0	79.0	101.0	0.00
	**b(OH/Cl)**	1.95	2.02	2.08	2.09	99.9	79.2	79.2	101.6	2.26
	**c(OH/COOH)**	1.93	2.03	2.09	2.08	101.7	79.1	79.1	100.3	3.62
	**d(OH/CN)**	1.94	2.02	2.08	2.08	100.6	79.3	79.3	100.7	0.59
	**e(CH_3/Cl)**	1.92	2.04	2.09	2.09	100.2	78.8	78.8	102.1	1.35
	**f(CH_3/COOH)**	1.90	2.05	2.08	2.08	102.1	78.6	78.6	100.7	3.78
	**g(CH_3/CN)**	1.91	2.04	2.09	2.09	100.8	78.8	78.8	101.7	1.50
	**h(NH_2/Cl)**	1.98	2.02	2.08	2.08	100.7	79.5	79.5	100.3	0.48
	**i(NH_2/COOH)**	1.97	2.02	2.08	2.08	101.0	79.4	79.4	100.4	0.08
	**j(NH_2/CN)**	1.98	2.02	2.08	2.08	99.9	79.4	79.4	101.2	0.61

Table S2. Benchmark of activation energies ($\Delta G^{\neq}$) for all carbenoids reactions using the M06-2X and ωB97XD functionals, energies are in kcal/mol.

	ΔG≠						
	Systems	*Cu*	*Ag*	*Au*	*Ni*	*Pd*	*Pt*
M06-2X	**a(H/H)**	4.61	5.20	4.90	0.00	0.00	4.68
	**b(OH/Cl)**	46.70	46.18	54.10	49.73	46.26	53.42
	**c(OH/COOH)**	31.07	29.44	40.87	36.33	32.66	41.66
	**d(OH/CN)**	32.83	31.38	41.46	36.10	33.93	41.50
	**e(CH_3/Cl)**	25.33	23.18	34.24	31.40	31.48	40.45
	**f(CH_3/COOH)**	11.01	9.50	20.62	16.27	14.59	15.96
	**g(CH_3/CN)**	13.21	11.61	23.96	15.92	15.43	25.54
	**h(NH_2/Cl)**	57.99	56.69	63.64	65.04	62.46	69.07
	**i(NH_2/COOH)**	45.66	45.45	55.43	58.22	53.95	62.98
	**j(NH_2/CN)**	49.93	45.81	56.36	54.85	52.51	58.89
	ΔE≠						
	Systems	*Cu*	*Ag*	*Au*	*Ni*	*Pd*	*Pt*
ωB97XD	**a(H/H)**	5.09	3.40	4.27	0.00	0.17	2.78
	**b(OH/Cl)**	52.30	49.56	55.94	54.26	49.89	55.34
	**c(OH/COOH)**	36.59	33.96	43.67	42.38	36.97	44.16
	**d(OH/CN)**	38.85	35.32	44.63	41.17	35.05	44.53
	**e(CH_3/Cl)**	30.83	27.52	37.35	36.52	35.97	43.01
	**f(CH_3/COOH)**	17.83	14.27	24.45	14.67	16.08	15.71
	**g(CH_3/CN)**	20.27	15.14	27.86	21.69	20.52	28.92
	**h(NH_2/Cl)**	61.18	60.21	65.35	68.42	66.24	69.90
	**i(NH_2/COOH)**	49.25	48.23	56.62	62.14	57.89	62.75
	**j(NH_2/CN)**	50.95	48.85	57.57	58.45	56.06	60.45

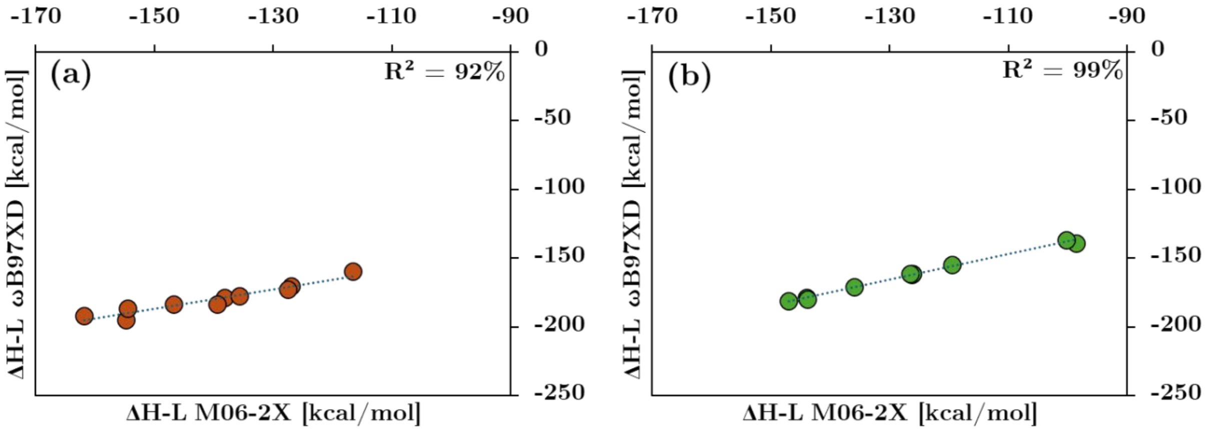

Figure S1. Calculated HOMO–LUMO gaps (ΔH-L) computed at the M06-2X and ωB97XD levels for **(a)** Cu and **(b)** Ni carbenoid systems, all orbital based energy values are reported in **Table S3**.

Table S3. HOMO and LUMO energies and corresponding gaps (ΔH-L) computed using the M06-2X and ωB97XD functionals for representative Cu and Ni carbenoid systems. All energies are in kcal/mol.

	Cu						Ni					
	M06-2X			ωB97XD			M06-2X			ωB97XD		
System	HOMO	LUMO	ΔH-L	HOMO	LUMO	ΔH-L	HOMO	LUMO	ΔH-L	HOMO	LUMO	ΔH-L
**a**	-270	-143	-127	-289	-118	-170	-344	-246	-99	-359	-220	-139
**b**	-265	-110	-155	-283	-88	-195	-340	-196	-144	-354	-175	-179
**c**	-264	-126	-138	-282	-104	-179	-339	-213	-126	-353	-191	-162
**d**	-267	-140	-128	-288	-115	-173	-344	-225	-120	-358	-203	-155
**e**	-266	-126	-139	-286	-103	-184	-339	-213	-126	-353	-192	-161
**f**	-264	-128	-136	-281	-104	-177	-337	-211	-127	-351	-189	-161
**g**	-269	-153	-117	-288	-128	-160	-342	-242	-100	-356	-219	-137
**h**	-263	-101	-162	-275	-84	-192	-338	-191	-147	-352	-171	-181
**i**	-262	-107	-154	-273	-86	-187	-337	-193	-144	-351	-171	-180
**j**	-265	-118	-147	-281	-97	-184	-340	-204	-136	-354	-183	-171

Table S4. Activation energies ($\Delta G^{\neq}$) for Cu and Ni carbenoid reactions using the M06-2X functional, energies are in kcal/mol.

Systems	*Cu*	*Ni*
**a(H/H)**	4.61	0.00
**b(OH/Cl)**	46.7	49.7
**c(OH/COOH)**	31.1	36.3
**d(OH/CN)**	32.8	36.1
**e(CH_3/Cl)**	25.3	31.4

**f(CH_3/COOH)**	11.0	16.3
**g(CH_3/CN)**	13.2	15.9
**h(NH_2/Cl)**	58.0	65.0
**i(NH_2/COOH)**	45.7	58.2
**j(NH_2/CN)**	49.9	54.9

Table S5. Calculated activation energies ($\Delta G^{\neq}$, kcal/mol) and quantified Dual Descriptor domains around the Cα atom ($\Delta f C\alpha$), the Grand Canonical Dual Descriptor (GCDD) ensemble scaled using softness (S) and hypersoftness (P) on the Cu and Ni systems.

Cu					*Ni*				
Systems	$\Delta G^{\neq}$	$\Delta f C\alpha$	*SGCDD*	*PGCDD*	*Systems*	$\Delta G^{\neq}$	$\Delta f C\alpha$	*SGCDD*	*PGCDD*
**a(H/H)**	4.61	0.39	0.31	0.35	**a(H/H)**	0.00	0.47	0.47	0.61
**b(OH/Cl)**	46.7	0.34	0.22	0.23	**b(OH/Cl)**	49.7	0.37	0.25	0.30
**c(OH/COOH)**	31.1	0.22	0.16	0.17	**c(OH/COOH)**	36.3	0.26	0.21	0.25
**d(OH/CN)**	32.8	0.25	0.20	0.22	**d(OH/CN)**	36.1	0.28	0.24	0.29
**e(CH_3/Cl)**	25.3	0.36	0.26	0.28	**e(CH_3/Cl)**	31.4	0.37	0.30	0.36
**f(CH_3/COOH)**	11.0	0.38	0.28	0.29	**f(CH_3/COOH)**	16.3	0.41	0.32	0.39
**g(CH_3/CN)**	13.2	0.28	0.24	0.27	**g(CH_3/CN)**	15.9	0.30	0.30	0.38
**h(NH_2/Cl)**	58.0	0.00	0.00	0.00	**h(NH_2/Cl)**	65.0	0.00	0.00	0.00
**i(NH_2/COOH)**	45.7	0.17	0.11	0.12	**i(NH_2/COOH)**	58.2	0.20	0.14	0.16
**j(NH_2/CN)**	49.9	0.20	0.14	0.15	**j(NH_2/CN)**	54.9	0.23	0.17	0.20

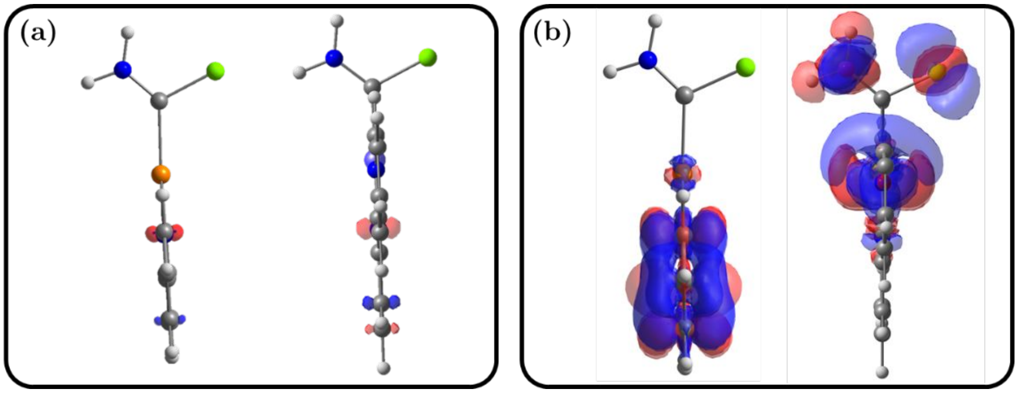

Figure S2. Dual Descriptor $\Delta f(\mathbf{r})$ for system **h** for **(a)** Cu in the and **(b)** Ni carbenoids obtained using the Frontier Molecular Orbital Approximation (FMOA; left) and Finite-Difference Approximation (FDA; right).

4.2.2. Electronic Structure and Reactivity Corrections for Carbenoid h.

Since the carbenoid **h** did not exhibit a measurable $\Delta f C\alpha$ value, the Dual Descriptor was recalculated using higher unoccupied molecular orbitals (LUMO+n) to recover the electrophilic region. In the Cu system, the LUMO+1 orbital localized the electrophilic site at Cα while in the Ni system this was achieved with the LUMO+2 orbital. These adjustments, based on the orbital-based finite difference approximation, resulted in an electrophilic region the Cα, enabling the computation of domain-integrated values for system **h** (Figure S3**a**, **b**).

Furthermore, re-evaluation of the correlation between the recalculated PGCDD and the activation barriers yielded lower R^2 values (70% for Cu and 82% for Ni systems) as shown in Figure S3**c**,**d**. This decrease reflects an increased PGCDD value for system **h**, which exceeds those of systems **i** and **j** despite comparable activation barriers, indicating an overestimation of the electrophilic contribution when using the $\Delta f C\alpha$ (LUMO+n) as a descriptor.

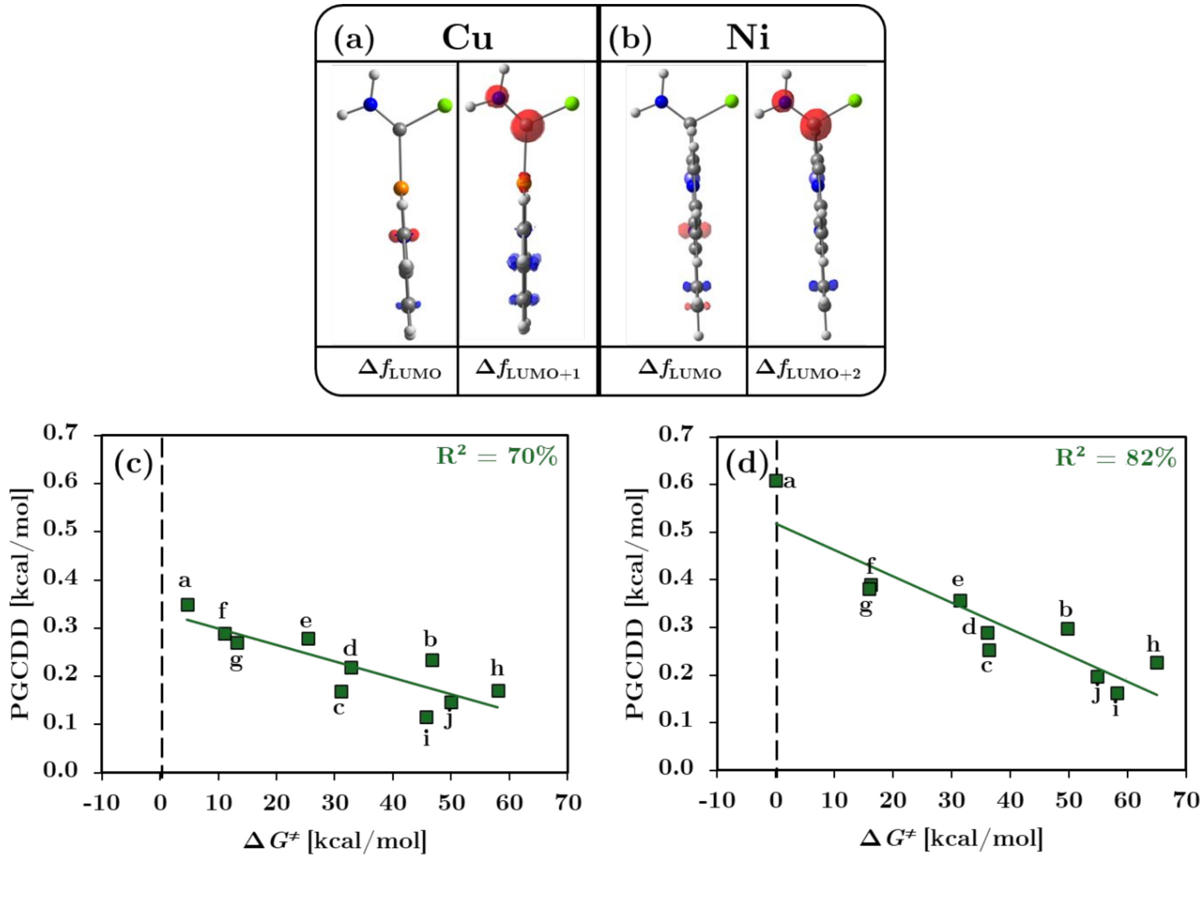

Figure S3. Electrophilic region for (**a**) Cu carbenoid using the LUMO+1 and (**b**) LUMO+2 for Ni. Correlation between activation barriers and the PGCDD for (**c**) Cu and (**d**) Ni in green. Plot values are presented in **Table S6**.

Table S6. Calculated activation energies ($\Delta G^{\neq}$) are in kcal/mol. The Grand Canonical Dual Descriptor (GCDD) around the Cα atom was scaled using hypersoftness (P) for Cu and Ni systems, employing the LUMO+1 and LUMO+2 orbitals, respectively, to determine the **h** value.

Cu			*Ni*		
System	*ΔG≠*	*PGCDD*	*System*	*ΔG≠*	*PGCDD*
**a(H/H)**	4.61	0.35	**a(H/H)**	0.00	0.61
**b(OH/Cl)**	46.7	0.23	**b(OH/Cl)**	49.7	0.30
**c(OH/COOH)**	31.1	0.17	**c(OH/COOH)**	36.3	0.25
**d(OH/CN)**	32.8	0.22	**d(OH/CN)**	36.1	0.29
**e(CH_3/Cl)**	25.3	0.28	**e(CH_3/Cl)**	31.4	0.36
**f(CH_3/COOH)**	11.0	0.29	**f(CH_3/COOH)**	16.3	0.39
**g(CH_3/CN)**	13.2	0.27	**g(CH_3/CN)**	15.9	0.38
**h(NH_2/Cl)**	58.0	0.28*	**h(NH_2/Cl)**	65.0	0.32*
**i(NH_2/COOH)**	45.7	0.12	**i(NH_2/COOH)**	58.2	0.16
**j(NH_2/CN)**	49.9	0.15	**j(NH_2/CN)**	54.9	0.20

*Calculated value using the LUMO+n approach

Table S7. Calculated activation energies ($\Delta G^{\neq}$) are in kcal/mol. The Grand Canonical Dual Descriptor (GCDD) around the Cα atom was scaled using hypersoftness (P) for Cu and Ni systems, employing the State Specific Dual Descriptor (SSDD) approach, to determine the **h** value.

Cu			*Ni*		
System	*ΔG≠*	*PGCDD*	*System*	*ΔG≠*	*PGCDD*
**a(H/H)**	4.61	0.35	**a(H/H)**	0.00	0.61
**b(OH/Cl)**	46.7	0.23	**b(OH/Cl)**	49.7	0.30
**c(OH/COOH)**	31.1	0.17	**c(OH/COOH)**	36.3	0.25
**d(OH/CN)**	32.8	0.22	**d(OH/CN)**	36.1	0.29
**e(CH_3/Cl)**	25.3	0.28	**e(CH_3/Cl)**	31.4	0.36
**f(CH_3/COOH)**	11.0	0.29	**f(CH_3/COOH)**	16.3	0.39
**g(CH_3/CN)**	13.2	0.27	**g(CH_3/CN)**	15.9	0.38
**h(NH_2/Cl)**	58.0	0.14*	**h(NH_2/Cl)**	65.0	0.16*
**i(NH_2/COOH)**	45.7	0.12	**i(NH_2/COOH)**	58.2	0.16
**j(NH_2/CN)**	49.9	0.15	**j(NH_2/CN)**	54.9	0.20

*Calculated value using the SSDD approach

Table S8. Activation energies ($\Delta G^{\neq}$) for Cu and Ni carbenoid reactions using the -COCl EWG, energies are in kcal/mol.

Systems	*Cu*	*Ni*
**a(H/H)**	4.61	0.00
**k(OH/COCl)**	29.0	30.5
**c(OH/COOH)**	31.1	36.3
**d(OH/CN)**	32.8	36.1
**l(CH_3/COCl)**	14.2	13.0
**f(CH_3/COOH)**	11.0	16.3
**g(CH_3/CN)**	13.2	15.9
**m(NH_2/COCl)**	43.4	53.8
**i(NH_2/COOH)**	45.7	58.2
**j(NH_2/CN)**	49.9	54.9

Table S9.Calculated activation energies ($\Delta G^{\neq}$) are in kcal/mol. The Dual Descriptor around the Cα atom was scaled using hypersoftness (P) for Cu and Ni systems, including the systems using -COCl as an EWG (**k**-**m**).

Cu			Ni		
system	$\Delta G^{\neq}$	*PGCDD*	system	$\Delta G^{\neq}$	*PGCDD*
**a(H/H)**	4.61	0.35	**a(H/H)**	0.00	0.61
**k(OH/COCl)**	29.0	0.15	**k(OH/COCl)**	30.5	0.22
**c(OH/COOH)**	31.1	0.17	**c(OH/COOH)**	36.3	0.25
**d(OH/CN)**	32.8	0.22	**d(OH/CN)**	36.1	0.29
**l(CH_3/COCl)**	14.2	0.31	**l(CH_3/COCl)**	13.0	0.43
**f(CH_3/COOH)**	11.0	0.29	**f(CH_3/COOH)**	16.3	0.39
**g(CH_3/CN)**	13.2	0.27	**g(CH_3/CN)**	15.9	0.38
**m(NH_2/COCl)**	43.4	0.10	**m(NH_2/COCl)**	53.8	0.14
**i(NH_2/COOH)**	45.7	0.12	**i(NH_2/COOH)**	58.2	0.16
**j(NH_2/CN)**	49.9	0.15	**j(NH_2/CN)**	54.9	0.20

Table S10. Cartesian coordinates (XYZ) for all studied structures.

Isolated systems			
Ni carbenoids			
a(H/H)			
28	-0.000003000	1.033625000	0.000019000
6	-0.000108000	2.961211000	0.000250000
6	4.260404000	1.408760000	-0.000094000
6	2.903572000	1.714382000	-0.000065000
7	1.947846000	0.773984000	-0.000009000
6	2.318956000	-0.538416000	-0.000002000
6	3.653649000	-0.916711000	-0.000032000
6	4.639927000	0.070882000	-0.000075000
1	2.578817000	2.758168000	-0.000097000
1	3.926757000	-1.971224000	-0.000030000
1	5.694469000	-0.206700000	-0.000102000
1	4.996116000	2.211914000	-0.000133000
6	1.179289000	-1.485280000	0.000012000
7	0.000012000	-0.849369000	-0.000010000
6	-1.179259000	-1.485293000	0.000011000
6	-1.215421000	-2.877185000	0.000046000
6	1.215466000	-2.877173000	0.000047000
1	-2.158624000	-3.421337000	0.000065000
1	0.000032000	-4.655647000	0.000093000
1	2.158675000	-3.421314000	0.000067000
6	-2.318937000	-0.538443000	-0.000004000
7	-1.947843000	0.773961000	-0.000008000
6	-2.903578000	1.714349000	-0.000062000
6	-4.260407000	1.408717000	-0.000096000
6	-4.639915000	0.070833000	-0.000079000
6	-3.653628000	-0.916749000	-0.000035000
1	-2.578836000	2.758143000	-0.000086000
1	-4.996127000	2.211863000	-0.000136000
1	-5.694454000	-0.206758000	-0.000108000
1	-3.926727000	-1.971265000	-0.000034000
6	0.000027000	-3.565137000	0.000062000
1	-0.000019000	3.595317000	-0.906496000
1	-0.000319000	3.594836000	0.907337000
b(OH/Cl)			
28	0.000333000	-0.518103000	-0.128984000
6	0.009988000	-2.502047000	-0.231628000
6	4.275845000	-0.845050000	-0.187929000
6	2.922259000	-1.169065000	-0.201847000
7	1.955574000	-0.245133000	-0.117912000
6	2.313163000	1.066831000	-0.016915000
6	3.642331000	1.462801000	0.003583000
6	4.641099000	0.491747000	-0.082876000
1	2.609188000	-2.209960000	-0.285000000
1	3.901582000	2.517603000	0.084817000
1	5.692030000	0.781973000	-0.068771000
1	5.020847000	-1.636233000	-0.259382000
6	1.165539000	1.999283000	0.062302000
7	-0.009810000	1.357707000	0.012144000
6	-1.192120000	1.986154000	0.065315000
6	-1.235505000	3.373245000	0.178282000
6	1.193744000	3.386770000	0.175193000
1	-2.182001000	3.909529000	0.223892000
1	-0.030550000	5.153091000	0.321507000
1	2.134303000	3.933588000	0.218427000
6	-2.329433000	1.040888000	-0.010907000
7	-1.957524000	-0.266925000	-0.113853000
6	-2.914004000	-1.201653000	-0.195264000
6	-4.271088000	-0.892847000	-0.176354000
6	-4.650961000	0.439711000	-0.069433000
6	-3.662889000	1.421898000	0.014070000
1	-2.589350000	-2.238714000	-0.281260000
1	-5.007406000	-1.692298000	-0.245655000
1	-5.705034000	0.718114000	-0.051646000
1	-3.933716000	2.473690000	0.096582000
6	-0.024629000	4.066268000	0.232719000
8	-0.025949000	-3.132175000	-1.336366000
1	-0.017193000	-4.116513000	-1.260686000
17	0.063990000	-3.452865000	1.176302000
c(OH/COOH)			
28	-0.018989000	-0.390925000	-0.207944000
6	-0.472321000	-2.308824000	-0.330401000
6	4.090011000	-1.629975000	-0.257001000
6	2.698591000	-1.652689000	-0.302381000
7	1.951250000	-0.549453000	-0.163061000
6	2.581608000	0.646081000	0.019533000
6	3.964040000	0.743297000	0.076651000
6	4.732435000	-0.413734000	-0.061085000
1	2.171309000	-2.591713000	-0.465769000
1	4.442184000	1.711011000	0.222502000
1	5.820615000	-0.358797000	-0.019710000
1	4.648074000	-2.557555000	-0.376627000
6	1.662999000	1.802136000	0.132021000
7	0.379242000	1.438833000	0.015138000

6	-0.637516000	2.308985000	0.079221000
6	-0.381446000	3.663410000	0.274384000
6	1.990279000	3.141495000	0.329094000
1	-1.188666000	4.392000000	0.330016000
1	1.177829000	5.123821000	0.552618000
1	3.025473000	3.464404000	0.427338000
6	-1.950816000	1.643785000	-0.084562000
7	-1.871761000	0.293121000	-0.247543000
6	-3.004737000	-0.398255000	-0.427599000
6	-4.259482000	0.202616000	-0.450087000
6	-4.341625000	1.579248000	-0.274054000
6	-3.167755000	2.310617000	-0.090040000
1	-2.913557000	-1.475647000	-0.574856000
1	-5.148407000	-0.407071000	-0.605616000
1	-5.307832000	2.084479000	-0.284440000
1	-3.203954000	3.391141000	0.042446000
6	0.949163000	4.068799000	0.398821000
6	-1.125677000	-3.154856000	0.794318000
8	-1.345175000	-4.315008000	0.579771000
8	-1.354765000	-2.447239000	1.874795000
1	-1.745321000	-3.011798000	2.573288000
8	-0.277350000	-3.041946000	-1.336307000
1	-0.605352000	-3.975164000	-1.137578000
d(OH/CN)			
28	-0.000003000	-0.589543000	-0.165910000
6	-0.000103000	-2.570855000	-0.302940000
6	4.270433000	-0.959288000	-0.103936000
6	2.915124000	-1.270398000	-0.162734000
7	1.954649000	-0.335297000	-0.123117000
6	2.321063000	0.974409000	-0.013147000
6	3.652882000	1.356712000	0.047784000
6	4.644532000	0.375302000	0.000508000
1	2.598005000	-2.311573000	-0.241100000
1	3.920056000	2.409059000	0.135845000
1	5.697233000	0.655619000	0.049029000
1	5.009005000	-1.759079000	-0.138054000
6	1.179723000	1.916310000	0.047398000
7	0.000079000	1.282203000	-0.003541000
6	-1.179510000	1.916413000	0.047372000
6	-1.214976000	3.304125000	0.154692000
6	1.215309000	3.304019000	0.154719000
1	-2.158216000	3.846350000	0.198526000
1	0.000242000	5.077545000	0.290801000
1	2.158595000	3.846161000	0.198575000
6	-2.320934000	0.974613000	-0.013191000
7	-1.954634000	-0.335126000	-0.123158000
6	-2.915192000	-1.270141000	-0.162786000
6	-4.270473000	-0.958912000	-0.103988000
6	-4.644455000	0.375711000	0.000448000
6	-3.652719000	1.357034000	0.047725000
1	-2.598167000	-2.311345000	-0.241145000
1	-5.009115000	-1.758639000	-0.138094000
1	-5.697132000	0.656121000	0.048970000
1	-3.919801000	2.409404000	0.135786000
6	0.000196000	3.990281000	0.206650000
6	-0.000354000	-3.298212000	0.939310000
7	-0.000552000	-3.725084000	2.018765000
8	-0.000086000	-3.339616000	-1.321544000
1	-0.000002000	-2.845095000	-2.167260000
e(CH_3/Cl)			
28	0.000944000	-0.532862000	0.109922000
6	0.015922000	-2.491274000	0.256857000
6	-4.283168000	-0.855427000	0.083105000
6	-2.930332000	-1.181263000	0.111033000
7	-1.959785000	-0.257521000	0.083418000
6	-2.314815000	1.057528000	0.017675000
6	-3.643564000	1.455865000	-0.012583000
6	-4.645189000	0.485062000	0.022430000
1	-2.620811000	-2.226027000	0.150502000
1	-3.899887000	2.513156000	-0.065795000
1	-5.695292000	0.777702000	-0.000923000
1	-5.029801000	-1.647954000	0.107425000
6	-1.167318000	1.993884000	-0.026084000
7	0.007884000	1.352369000	-0.001772000
6	1.188649000	1.983711000	-0.033378000
6	1.231208000	3.374176000	-0.096433000
6	-1.198071000	3.384782000	-0.088946000
1	2.176711000	3.913442000	-0.123557000
1	0.024004000	5.156584000	-0.173356000
1	-2.138963000	3.932287000	-0.110277000
6	2.328267000	1.037339000	0.003095000
7	1.961741000	-0.274160000	0.074105000
6	2.923047000	-1.207869000	0.095285000
6	4.278588000	-0.895280000	0.054385000
6	4.652754000	0.441783000	-0.010990000
6	3.660592000	1.422542000	-0.038505000
1	2.603583000	-2.249847000	0.142254000
1	5.017814000	-1.694861000	0.073215000
1	5.705446000	0.724117000	-0.043659000
1	3.926980000	2.477182000	-0.094923000

6	0.019422000	4.067268000	-0.124065000
17	-0.105112000	-3.356836000	-1.164369000
6	0.112990000	-3.270324000	1.490569000
1	-0.868954000	-3.111788000	1.992046000
1	0.289534000	-4.346792000	1.371160000
1	0.827326000	-2.790693000	2.181389000
f(CH_3/COOH)			
28	-0.012762000	-0.391656000	0.145426000
6	-0.112081000	-2.329584000	0.494443000
6	-4.299894000	-0.529147000	0.096689000
6	-2.962455000	-0.913032000	0.131315000
7	-1.954889000	-0.029146000	0.097080000
6	-2.252485000	1.298189000	0.004436000
6	-3.563096000	1.751969000	-0.034714000
6	-4.604602000	0.824684000	0.017087000
1	-2.695828000	-1.970198000	0.168977000
1	-3.774257000	2.817923000	-0.109036000
1	-5.641341000	1.161245000	-0.011470000
1	-5.079909000	-1.288505000	0.128362000
6	-1.064440000	2.180503000	-0.062275000
7	0.080824000	1.485694000	-0.036089000
6	1.288983000	2.061840000	-0.074330000
6	1.393510000	3.448636000	-0.158084000
6	-1.032632000	3.569600000	-0.145583000
1	2.362729000	3.943518000	-0.192165000
1	0.268988000	5.282600000	-0.260518000
1	-1.947870000	4.158864000	-0.169799000
6	2.384003000	1.066407000	-0.020414000
7	1.958931000	-0.226449000	0.078420000
6	2.876350000	-1.203861000	0.098648000
6	4.244769000	-0.948150000	0.042493000
6	4.678844000	0.368223000	-0.041946000
6	3.730456000	1.392201000	-0.078509000
1	2.507460000	-2.228456000	0.135890000
1	4.946538000	-1.780787000	0.060329000
1	5.742616000	0.603161000	-0.086969000
1	4.042186000	2.432861000	-0.157216000
6	0.215068000	4.195489000	-0.195094000
6	-0.224971000	-3.271692000	-0.632279000
8	0.871248000	-3.502839000	-1.078418000
8	-1.397872000	-3.700881000	-1.024084000
1	-1.297920000	-4.306833000	-1.788169000
6	0.066164000	-2.912867000	1.813701000
1	-1.003576000	-2.875397000	2.158367000
1	0.354635000	-3.973654000	1.839205000

1	0.622872000	-2.287357000	2.522935000
g(CH_3/CN)			
28	0.000999000	-0.592255000	-0.114762000
6	0.004938000	-2.557837000	-0.304649000
6	4.286062000	-0.853254000	-0.059318000
6	2.938355000	-1.198528000	-0.095803000
7	1.954471000	-0.288237000	-0.084495000
6	2.290086000	1.032365000	-0.022986000
6	3.612639000	1.449688000	0.013721000
6	4.628314000	0.492831000	-0.007112000
1	2.645804000	-2.248805000	-0.125081000
1	3.853482000	2.510810000	0.063628000
1	5.673972000	0.800539000	0.022822000
1	5.043846000	-1.635489000	-0.068628000
6	1.128107000	1.949946000	0.016933000
7	-0.036679000	1.288636000	0.002600000
6	-1.228399000	1.899964000	0.038767000
6	-1.293775000	3.289683000	0.094540000
6	1.135096000	3.341419000	0.072044000
1	-2.248258000	3.812802000	0.125092000
1	-0.116379000	5.092797000	0.153417000
1	2.066794000	3.904754000	0.085108000
6	-2.350767000	0.933453000	0.020030000
7	-1.959669000	-0.370780000	-0.054276000
6	-2.901553000	-1.324729000	-0.048325000
6	-4.261842000	-1.039816000	0.020973000
6	-4.661605000	0.290100000	0.085025000
6	-3.689581000	1.291464000	0.086287000
1	-2.562248000	-2.360906000	-0.095987000
1	-4.984668000	-1.854519000	0.025539000
1	-5.719025000	0.550969000	0.138619000
1	-3.976452000	2.340685000	0.144003000
6	-0.093643000	4.003417000	0.110310000
6	0.260479000	-3.293406000	0.871646000
7	0.476701000	-3.822232000	1.888998000
6	-0.197262000	-3.298695000	-1.538543000
1	0.867377000	-3.356863000	-1.898843000
1	-0.525907000	-4.342332000	-1.431228000
1	-0.723985000	-2.731387000	-2.316160000
h(NH_2/Cl)			
28	-0.000003000	-0.527178000	0.159733000
6	-0.000312000	-2.504577000	0.284907000
6	-4.275153000	-0.878738000	0.103683000
6	-2.920262000	-1.194645000	0.156963000
7	-1.958537000	-0.262547000	0.120054000

6	-2.321564000	1.047765000	0.019143000
6	-3.652326000	1.435656000	-0.035756000
6	-4.646455000	0.457088000	0.008549000
1	-2.598933000	-2.233899000	0.227862000
1	-3.916235000	2.489338000	-0.116393000
1	-5.698415000	0.740626000	-0.034875000
1	-5.016517000	-1.675865000	0.135528000
6	-1.178154000	1.988232000	-0.037278000
7	0.000252000	1.352566000	0.007412000
6	1.178834000	1.987905000	-0.037307000
6	1.215204000	3.376679000	-0.132950000
6	-1.214138000	3.377015000	-0.132927000
1	2.158660000	3.918825000	-0.171218000
1	0.000777000	5.151603000	-0.254559000
1	-2.157445000	3.919423000	-0.171180000
6	2.321982000	1.047115000	0.019056000
7	1.958598000	-0.263095000	0.119978000
6	2.920065000	-1.195467000	0.156775000
6	4.275039000	-0.879939000	0.103391000
6	4.646707000	0.455788000	0.008276000
6	3.652849000	1.434634000	-0.035930000
1	2.598441000	-2.234630000	0.227676000
1	5.016183000	-1.677276000	0.135144000
1	5.698744000	0.739032000	-0.035220000
1	3.917047000	2.488244000	-0.116564000
6	0.000628000	4.063741000	-0.179662000
17	-0.001429000	-3.338905000	-1.207958000
7	0.000202000	-3.265786000	1.334800000
1	0.000853000	-2.849980000	2.267326000
1	-0.000054000	-4.290547000	1.279945000
i(NH_2/COOH)			
28	-0.000010000	0.399598000	-0.250783000
6	-0.000216000	2.365060000	-0.460634000
6	-4.276721000	0.746368000	-0.228503000
6	-2.921761000	1.059334000	-0.293136000
7	-1.959317000	0.132127000	-0.203637000
6	-2.321537000	-1.170970000	-0.035800000
6	-3.652545000	-1.556714000	0.035408000
6	-4.647293000	-0.583016000	-0.063393000
1	-2.601470000	2.093183000	-0.425697000
1	-3.915846000	-2.605097000	0.169304000
1	-5.699183000	-0.864966000	-0.009426000
1	-5.018669000	1.539750000	-0.307081000
6	-1.177712000	-2.107375000	0.069046000
7	0.000149000	-1.474621000	-0.006626000
6	1.178117000	-2.107176000	0.069059000
6	1.214919000	-3.489726000	0.232805000
6	-1.214283000	-3.489932000	0.232792000
1	2.158368000	-4.029495000	0.296879000
1	0.000466000	-5.256691000	0.440580000
1	-2.157641000	-4.029860000	0.296855000
6	2.321782000	-1.170574000	-0.035774000
7	1.959343000	0.132463000	-0.203609000
6	2.921632000	1.059834000	-0.293091000
6	4.276644000	0.747095000	-0.228442000
6	4.647439000	-0.582226000	-0.063337000
6	3.652854000	-1.556094000	0.035446000
1	2.601164000	2.093628000	-0.425642000
1	5.018458000	1.540604000	-0.307003000
1	5.699376000	-0.864000000	-0.009361000
1	3.916333000	-2.604433000	0.169338000
6	0.000375000	-4.173737000	0.313119000
6	-0.000552000	3.237582000	0.806442000
8	-0.000573000	4.433541000	0.763193000
8	-0.000342000	2.467543000	1.885612000
1	-0.000353000	3.030816000	2.685038000
7	-0.000166000	3.112695000	-1.507109000
1	0.000023000	2.733871000	-2.455044000
1	-0.000340000	4.140227000	-1.392436000
j(NH_2/CN)			
28	-0.000015000	-0.591017000	-0.165045000
6	-0.000426000	-2.579389000	-0.318261000
6	4.273537000	-0.949288000	-0.063183000
6	2.918948000	-1.262847000	-0.131511000
7	1.957779000	-0.329232000	-0.112183000
6	2.321841000	0.981047000	-0.012020000
6	3.652559000	1.366913000	0.055591000
6	4.645691000	0.386663000	0.028064000
1	2.599082000	-2.302947000	-0.199588000
1	3.917190000	2.420517000	0.135157000
1	5.697533000	0.668794000	0.082888000
1	5.013613000	-1.748116000	-0.079357000
6	1.179110000	1.922209000	0.035299000
7	0.000297000	1.286715000	-0.009051000
6	-1.178306000	1.922601000	0.035250000
6	-1.214020000	3.311554000	0.127003000
6	1.215284000	3.311149000	0.127051000
1	-2.157399000	3.853918000	0.164832000
1	0.000924000	5.086606000	0.242704000
1	2.158842000	3.853200000	0.164921000

6	-2.321351000	0.981827000	-0.012155000
7	-1.957723000	-0.328567000	-0.112381000
6	-2.919205000	-1.261856000	-0.131865000
6	-4.273692000	-0.947848000	-0.063610000
6	-4.645403000	0.388220000	0.027741000
6	-3.651944000	1.368135000	0.055409000
1	-2.599696000	-2.302062000	-0.200022000
1	-5.014036000	-1.746425000	-0.079919000
1	-5.697154000	0.670697000	0.082527000
1	-3.916225000	2.421822000	0.135040000
6	0.000745000	3.998512000	0.171019000
6	-0.001523000	-3.286301000	0.934458000
7	-0.002471000	-3.746062000	1.999317000
7	0.000119000	-3.313186000	-1.385688000
1	0.000876000	-2.883832000	-2.312519000
1	-0.000202000	-4.339176000	-1.361199000
k(OH/COCl)			
28	0.000057000	-0.255860000	-0.343502000
6	0.001866000	-2.208598000	-0.656908000
6	4.273052000	-0.599971000	-0.416122000
6	2.917757000	-0.910654000	-0.472736000
7	1.955563000	0.005312000	-0.298179000
6	2.319710000	1.296873000	-0.057161000
6	3.651446000	1.679541000	0.012286000
6	4.645017000	0.716438000	-0.169119000
1	2.600423000	-1.934709000	-0.674072000
1	3.916366000	2.718315000	0.205173000
1	5.697562000	0.996753000	-0.118482000
1	5.013840000	-1.384174000	-0.565855000
6	1.176584000	2.222876000	0.115316000
7	-0.001539000	1.595674000	-0.001661000
6	-1.180737000	2.220842000	0.115409000
6	-1.218056000	3.589216000	0.370313000
6	1.211562000	3.591313000	0.370225000
1	-2.161981000	4.122841000	0.469862000
1	-0.004745000	5.339348000	0.695153000
1	2.154573000	4.126564000	0.469709000
6	-2.322263000	1.292845000	-0.056975000
7	-1.955885000	0.001923000	-0.298034000
6	-2.916490000	-0.915733000	-0.472487000
6	-4.272320000	-0.607415000	-0.415735000
6	-4.646560000	0.708343000	-0.168714000
6	-3.654658000	1.673186000	0.012593000
1	-2.597359000	-1.939221000	-0.673852000
1	-5.011750000	-1.392913000	-0.565390000

1	-5.699588000	0.986823000	-0.117987000
1	-3.921382000	2.711495000	0.205490000
6	-0.003825000	4.267226000	0.495960000
8	0.001989000	-2.755827000	-1.791850000
1	0.003099000	-3.755570000	-1.711117000
6	0.003477000	-3.298452000	0.460407000
8	0.004701000	-4.445054000	0.146173000
17	0.003264000	-2.667767000	2.055920000
l(CH_3/COCl)			
28	0.000241000	0.242488000	-0.265906000
6	-0.245006000	2.177105000	-0.637627000
6	-4.295967000	0.044222000	-0.351820000
6	-2.991357000	0.526623000	-0.399883000
7	-1.918179000	-0.261882000	-0.244341000
6	-2.118019000	-1.593585000	-0.028358000
6	-3.390278000	-2.143726000	0.032509000
6	-4.498949000	-1.312899000	-0.131970000
1	-2.809293000	1.588881000	-0.561361000
1	-3.519829000	-3.210895000	0.207626000
1	-5.507177000	-1.725726000	-0.087180000
1	-5.129840000	0.732177000	-0.484188000
6	-0.871092000	-2.374862000	0.130770000
7	0.222445000	-1.605678000	0.040131000
6	1.467267000	-2.090117000	0.144029000
6	1.667584000	-3.451158000	0.361220000
6	-0.742562000	-3.743929000	0.347966000
1	2.668715000	-3.869702000	0.450431000
1	0.673317000	-5.342464000	0.633788000
1	-1.615012000	-4.390558000	0.426187000
6	2.491878000	-1.031832000	0.004742000
7	1.979863000	0.217482000	-0.193320000
6	2.833052000	1.245365000	-0.314978000
6	4.215473000	1.086504000	-0.258067000
6	4.736408000	-0.186216000	-0.063630000
6	3.857609000	-1.262122000	0.072728000
1	2.406486000	2.240927000	-0.436460000
1	4.859564000	1.958784000	-0.360288000
1	5.813742000	-0.346395000	-0.012559000
1	4.237929000	-2.270107000	0.232881000
6	0.544307000	-4.273195000	0.463143000
6	0.041914000	3.142142000	0.443007000
8	1.044966000	3.725675000	0.687125000
17	-1.367785000	3.282669000	1.417404000
6	-0.617672000	2.751241000	-1.910818000
1	-1.055796000	3.760716000	-1.894685000

1	0.445206000	2.904556000	-2.268484000
1	-1.078575000	2.062566000	-2.628269000
m(NH_2/COCl)			
28	0.170699000	0.001411000	-0.316286000
6	2.145919000	0.014116000	-0.585661000
6	0.494738000	4.280591000	-0.285325000
6	0.811063000	2.928252000	-0.377039000
7	-0.105600000	1.959218000	-0.255552000
6	-1.401665000	2.312866000	-0.026780000
6	-1.790964000	3.640921000	0.073656000
6	-0.827973000	4.642086000	-0.058125000
1	1.840225000	2.615907000	-0.556383000
1	-2.833559000	3.896760000	0.257664000
1	-1.112575000	5.691780000	0.019664000
1	1.280285000	5.027598000	-0.389969000
6	-2.324818000	1.163099000	0.114531000
7	-1.688988000	-0.010713000	0.005330000
6	-2.309530000	-1.192692000	0.114488000
6	-3.682109000	-1.238078000	0.344967000
6	-3.697870000	1.190661000	0.344998000
1	-4.211388000	-2.185112000	0.437301000
1	-5.445021000	-0.035100000	0.637209000
1	-4.239420000	2.130727000	0.437366000
6	-1.371448000	-2.330322000	-0.026972000
7	-0.080041000	-1.959829000	-0.255480000
6	0.849139000	-2.916847000	-0.377205000
6	0.550361000	-4.273194000	-0.286006000
6	-0.767587000	-4.651882000	-0.059150000
6	-1.743500000	-3.663346000	0.072902000
1	1.874177000	-2.591056000	-0.556355000
1	1.345529000	-5.009909000	-0.390860000
1	-1.038586000	-5.705199000	0.018103000
1	-2.782723000	-3.932765000	0.256572000
6	-4.369588000	-0.028115000	0.457210000
6	2.875207000	0.017396000	0.762333000
8	2.231411000	0.012770000	1.759641000
17	4.624899000	0.027945000	0.739669000
7	2.881898000	0.020098000	-1.640227000
1	2.470300000	0.018521000	-2.576356000
1	3.911491000	0.026605000	-1.584872000
Pd carbenoids			
a(H/H)			
46	-0.000006000	1.087889000	0.000021000
6	0.000120000	2.967878000	0.000004000
6	-0.000021000	-3.673101000	-0.000052000
6	4.423486000	1.141751000	-0.000004000
6	3.090263000	1.537616000	0.000012000
7	2.075453000	0.659572000	0.000007000
6	2.352826000	-0.678414000	-0.000018000
6	3.664815000	-1.134439000	-0.000035000
6	4.715085000	-0.216611000	-0.000027000
1	2.830656000	2.595987000	0.000027000
1	3.872198000	-2.203460000	-0.000056000
1	5.748228000	-0.565212000	-0.000040000
1	5.208299000	1.897193000	0.000003000
6	1.181770000	-1.591125000	-0.000027000
7	-0.000014000	-0.957174000	-0.000012000
6	-1.181802000	-1.591120000	-0.000015000
6	-1.213382000	-2.984567000	-0.000035000
6	1.213343000	-2.984573000	-0.000048000
1	-2.154320000	-3.532000000	-0.000039000
1	-0.000023000	-4.763491000	-0.000068000
1	2.154278000	-3.532010000	-0.000059000
6	-2.352846000	-0.678399000	0.000002000
7	-2.075447000	0.659582000	0.000017000
6	-3.090241000	1.537645000	0.000033000
6	-4.423472000	1.141801000	0.000034000
6	-4.715095000	-0.216555000	0.000020000
6	-3.664842000	-1.134401000	0.000004000
1	-2.830610000	2.596008000	0.000048000
1	-5.208272000	1.897257000	0.000045000
1	-5.748245000	-0.565137000	0.000021000
1	-3.872243000	-2.203419000	-0.000005000
1	0.000467000	3.570595000	-0.925940000
1	-0.000135000	3.570609000	0.925939000
b(OH/Cl)			
46	-0.010323000	-0.582352000	-0.093929000
6	-0.156182000	-2.597641000	-0.141436000
6	0.388779000	4.111902000	0.245069000
6	4.386189000	-1.044459000	-0.111769000
6	3.022227000	-1.323454000	-0.140245000
7	2.089665000	-0.366440000	-0.064595000
6	2.481434000	0.937994000	0.034699000
6	3.826453000	1.281358000	0.068313000
6	4.793838000	0.278504000	-0.003183000
1	2.667271000	-2.348228000	-0.234235000
1	4.125133000	2.325573000	0.146610000
1	5.853054000	0.535608000	0.022878000
1	5.104098000	-1.861011000	-0.175517000
6	1.390580000	1.941884000	0.092358000

7	0.157056000	1.415334000	0.020457000
6	-0.968300000	2.147158000	0.054806000
6	-0.877944000	3.533206000	0.169247000
6	1.538270000	3.322659000	0.207287000
1	-1.769158000	4.157405000	0.200833000
1	0.481770000	5.194474000	0.335705000
1	2.522639000	3.783284000	0.268357000
6	-2.214252000	1.347534000	-0.045003000
7	-2.052327000	-0.005693000	-0.131966000
6	-3.135515000	-0.782877000	-0.249276000
6	-4.429632000	-0.269406000	-0.277591000
6	-4.603307000	1.104660000	-0.178991000
6	-3.478526000	1.921853000	-0.063140000
1	-2.966310000	-1.855871000	-0.324143000
1	-5.275872000	-0.947875000	-0.376532000
1	-5.601140000	1.543807000	-0.195326000
1	-3.592315000	3.002384000	0.008695000
8	0.475327000	-3.293598000	-1.002645000
1	0.342265000	-4.268523000	-0.929464000
17	-1.080273000	-3.464218000	0.991759000
c(OH/COOH)			
46	-0.035616000	-0.463359000	-0.159585000
6	-0.487888000	-2.396995000	-0.267923000
6	1.073383000	4.103909000	0.384806000
6	4.240556000	-1.586082000	-0.175041000
6	2.850285000	-1.651695000	-0.220846000
7	2.073845000	-0.568234000	-0.100877000
6	2.658357000	0.655812000	0.059665000
6	4.039641000	0.786947000	0.115339000
6	4.843587000	-0.347648000	0.000073000
1	2.344646000	-2.604882000	-0.367650000
1	4.493274000	1.768634000	0.242687000
1	5.929248000	-0.256699000	0.042198000
1	4.826099000	-2.498710000	-0.277603000
6	1.733938000	1.814257000	0.150431000
7	0.437173000	1.488582000	0.034971000
6	-0.563100000	2.381920000	0.081849000
6	-0.265463000	3.731694000	0.260321000
6	2.089523000	3.149858000	0.330540000
1	-1.050189000	4.484803000	0.304444000
1	1.328756000	5.154419000	0.526249000
1	3.130937000	3.451126000	0.428981000
6	-1.914031000	1.786186000	-0.079996000
7	-1.960178000	0.428429000	-0.217204000
6	-3.145735000	-0.166016000	-0.396698000

6	-4.343162000	0.541811000	-0.444579000
6	-4.306116000	1.922077000	-0.293620000
6	-3.074113000	2.549775000	-0.110178000
1	-3.141112000	-1.249162000	-0.510391000
1	-5.279870000	0.008121000	-0.599512000
1	-5.222982000	2.511315000	-0.322990000
1	-3.022477000	3.631640000	0.001856000
6	-1.410163000	-3.192478000	0.695703000
8	-1.525887000	-4.372241000	0.512838000
8	-1.948629000	-2.436799000	1.622246000
1	-2.488488000	-2.984501000	2.228834000
8	-0.003320000	-3.182530000	-1.128564000
1	-0.359683000	-4.110485000	-0.965679000
d(OH/CN)			
46	-0.013135000	-0.647047000	-0.134918000
6	-0.167768000	-2.642106000	-0.263572000
6	0.325292000	4.051443000	0.171576000
6	4.384111000	-1.075110000	0.034498000
6	3.025461000	-1.364742000	-0.050045000
7	2.082807000	-0.411921000	-0.051817000
6	2.459425000	0.898028000	0.043509000
6	3.799970000	1.251376000	0.127832000
6	4.776506000	0.254905000	0.121411000
1	2.684766000	-2.398719000	-0.111158000
1	4.087487000	2.299034000	0.203731000
1	5.831757000	0.521076000	0.189157000
1	5.109359000	-1.887894000	0.034429000
6	1.357578000	1.893220000	0.067629000
7	0.130383000	1.350676000	0.011704000
6	-1.006273000	2.065165000	0.031798000
6	-0.934388000	3.454802000	0.113807000
6	1.486765000	3.277963000	0.149764000
1	-1.833489000	4.068344000	0.132047000
1	0.403641000	5.137165000	0.235215000
1	2.464424000	3.754583000	0.196336000
6	-2.239476000	1.241287000	-0.034920000
7	-2.053957000	-0.109867000	-0.125073000
6	-3.123473000	-0.914793000	-0.172105000
6	-4.428117000	-0.429192000	-0.139640000
6	-4.626261000	0.942529000	-0.053306000
6	-3.515737000	1.786350000	0.000724000
1	-2.936539000	-1.987007000	-0.233284000
1	-5.262782000	-1.127920000	-0.180062000
1	-5.633340000	1.359554000	-0.024813000
1	-3.650691000	2.864427000	0.074650000

6	-0.740884000	-3.351352000	0.849777000
7	-1.249138000	-3.779543000	1.800876000
8	0.179026000	-3.412819000	-1.220693000
1	0.540351000	-2.919984000	-1.986725000
	e(CH_3/Cl)		
46	-0.005270000	-0.602856000	0.096758000
6	-0.046686000	-2.559215000	0.230720000
6	0.162557000	4.132952000	-0.109797000
6	-4.437396000	-0.508813000	0.094141000
6	-3.118547000	-0.953327000	0.117121000
7	-2.073347000	-0.117021000	0.085102000
6	-2.299599000	1.228052000	0.021684000
6	-3.592825000	1.734912000	-0.004857000
6	-4.677043000	0.858039000	0.032962000
1	-2.894523000	-2.019235000	0.158693000
1	-3.760267000	2.809542000	-0.057747000
1	-5.696131000	1.245101000	0.012573000
1	-5.251140000	-1.232199000	0.123109000
6	-1.093855000	2.096409000	-0.022856000
7	0.065582000	1.422794000	-0.011249000
6	1.270374000	2.011282000	-0.044167000
6	1.350871000	3.401960000	-0.095319000
6	-1.074678000	3.489480000	-0.073729000
1	2.310382000	3.915338000	-0.122899000
1	0.201370000	5.221875000	-0.149145000
1	-1.995032000	4.070676000	-0.084055000
6	2.411117000	1.057853000	-0.026036000
7	2.089421000	-0.267638000	0.039558000
6	3.071648000	-1.178284000	0.040700000
6	4.418151000	-0.831221000	-0.015361000
6	4.754969000	0.515336000	-0.074488000
6	3.737105000	1.469018000	-0.081199000
1	2.772004000	-2.225818000	0.084235000
1	5.177520000	-1.612021000	-0.013468000
1	5.798774000	0.827277000	-0.118683000
1	3.981294000	2.528956000	-0.132698000
17	-0.501784000	-3.440892000	-1.112249000
6	0.277414000	-3.316828000	1.443816000
1	-0.677010000	-3.316816000	2.018775000
1	0.557952000	-4.364479000	1.268473000
1	0.994522000	-2.774216000	2.076303000
	f(CH_3/COOH)		
46	0.004151000	-0.474591000	0.177054000
6	0.084404000	-2.394653000	0.507207000
6	-0.147862000	4.249909000	-0.179028000

6	-4.407172000	-0.710894000	0.021168000
6	-3.060615000	-1.054792000	0.093584000
7	-2.082538000	-0.138864000	0.087350000
6	-2.405795000	1.183802000	-0.017423000
6	-3.732198000	1.590042000	-0.090854000
6	-4.747175000	0.633470000	-0.065723000
1	-2.753925000	-2.099944000	0.142535000
1	-3.978090000	2.647613000	-0.174248000
1	-5.791615000	0.941304000	-0.122289000
1	-5.164851000	-1.493240000	0.030966000
6	-1.264646000	2.135380000	-0.067556000
7	-0.061637000	1.542735000	-0.043074000
6	1.101278000	2.209614000	-0.071976000
6	1.087054000	3.602074000	-0.144452000
6	-1.339933000	3.525071000	-0.139698000
1	2.010032000	4.178731000	-0.170133000
1	-0.182326000	5.338293000	-0.234129000
1	-2.297545000	4.042391000	-0.161500000
6	2.300973000	1.333578000	-0.026648000
7	2.064375000	-0.008372000	0.075957000
6	3.098166000	-0.860713000	0.076373000
6	4.419687000	-0.426760000	0.000451000
6	4.672284000	0.935762000	-0.084013000
6	3.596860000	1.825348000	-0.103429000
1	2.858447000	-1.923501000	0.116395000
1	5.225521000	-1.159479000	0.004126000
1	5.694251000	1.310924000	-0.143625000
1	3.773405000	2.896744000	-0.185623000
6	0.211359000	-3.291083000	-0.669666000
8	1.352578000	-3.504515000	-0.984846000
8	-0.904567000	-3.694791000	-1.230677000
1	-0.705619000	-4.263694000	-2.003448000
6	0.164336000	-3.007919000	1.825660000
1	-0.878792000	-3.380268000	1.982601000
1	0.782183000	-3.921785000	1.829600000
1	0.406449000	-2.320367000	2.642989000
	g(CH_3/CN)		
46	0.011644000	-0.663222000	-0.102609000
6	0.122468000	-2.617180000	-0.237866000
6	-0.295090000	4.062203000	0.105866000
6	4.435945000	-0.444553000	-0.111380000
6	3.130289000	-0.926824000	-0.135120000
7	2.061602000	-0.119957000	-0.104222000
6	2.250354000	1.231626000	-0.040025000
6	3.528508000	1.774234000	-0.014020000

6	4.637328000	0.928185000	-0.051275000
1	2.940743000	-1.999323000	-0.173834000
1	3.665420000	2.853157000	0.039921000
1	5.645176000	1.343701000	-0.030107000
1	5.269018000	-1.145778000	-0.137878000
6	1.021190000	2.063772000	0.010519000
7	-0.118482000	1.355863000	0.008408000
6	-1.340396000	1.908710000	0.052381000
6	-1.461320000	3.296086000	0.103003000
6	0.960293000	3.455598000	0.060243000
1	-2.435574000	3.780405000	0.139538000
1	-0.365989000	5.149576000	0.144440000
1	1.863219000	4.063672000	0.062485000
6	-2.450716000	0.921904000	0.050351000
7	-2.087751000	-0.393136000	-0.014726000
6	-3.040492000	-1.334955000	0.004301000
6	-4.396203000	-1.029923000	0.079442000
6	-4.775044000	0.305498000	0.135865000
6	-3.787977000	1.291028000	0.122570000
1	-2.709141000	-2.372569000	-0.037543000
1	-5.129923000	-1.834795000	0.095013000
1	-5.827548000	0.584243000	0.194340000
1	-4.064526000	2.343077000	0.172743000
6	0.737488000	-3.280596000	0.851659000
7	1.274704000	-3.758194000	1.768849000
6	-0.322247000	-3.424946000	-1.367610000
1	0.632676000	-3.585710000	-1.931350000
1	-0.650987000	-4.439733000	-1.091917000
1	-1.004953000	-2.902945000	-2.047447000

h(NH_2/Cl)

46	0.008487000	-0.578767000	-0.134570000
6	0.130512000	-2.595778000	-0.256209000
6	-0.247101000	4.128211000	0.164395000
6	4.428048000	-0.435279000	-0.114182000
6	3.115148000	-0.898156000	-0.150211000
7	2.060642000	-0.075047000	-0.110518000
6	2.268413000	1.271754000	-0.023504000
6	3.553727000	1.796274000	0.015782000
6	4.649262000	0.933362000	-0.031642000
1	2.901979000	-1.965509000	-0.208266000
1	3.706865000	2.872053000	0.086595000
1	5.663155000	1.333126000	-0.001029000
1	5.251814000	-1.146898000	-0.149237000
6	1.048213000	2.117778000	0.035056000
7	-0.100199000	1.423867000	0.009535000
6	-1.316884000	1.988476000	0.056334000
6	-1.421872000	3.375914000	0.136174000
6	1.001854000	3.508727000	0.114587000
1	-2.391008000	3.870142000	0.175444000
1	-0.306034000	5.215172000	0.226190000
1	1.911720000	4.105921000	0.137040000
6	-2.437767000	1.013340000	0.021232000
7	-2.085190000	-0.302241000	-0.075927000
6	-3.043887000	-1.236478000	-0.091671000
6	-4.398206000	-0.921732000	-0.020138000
6	-4.766572000	0.414610000	0.071232000
6	-3.772032000	1.392734000	0.093340000
1	-2.715286000	-2.273500000	-0.160180000
1	-5.139425000	-1.719620000	-0.034488000
1	-5.817232000	0.700136000	0.129171000
1	-4.040783000	2.445147000	0.171151000
17	0.571218000	-3.455436000	1.150423000
7	-0.092638000	-3.326537000	-1.305505000
1	-0.343501000	-2.884647000	-2.190883000
1	-0.006832000	-4.348677000	-1.295015000

i(NH_2/COOH)

46	-0.029721000	-0.460635000	-0.210969000
6	-0.379357000	-2.430569000	-0.412880000
6	0.742482000	4.178935000	0.296433000
6	4.317645000	-1.283442000	-0.086918000
6	2.937884000	-1.445255000	-0.177754000
7	2.084715000	-0.415074000	-0.122532000
6	2.577345000	0.848728000	0.034828000
6	3.944351000	1.077166000	0.130130000
6	4.827719000	-0.000651000	0.067779000
1	2.500751000	-2.436609000	-0.296442000
1	4.324650000	2.089730000	0.256041000
1	5.902494000	0.166470000	0.142801000
1	4.968055000	-2.155706000	-0.135947000
6	1.570400000	1.940200000	0.104673000
7	0.300509000	1.516681000	0.020185000
6	-0.763794000	2.331978000	0.063530000
6	-0.566367000	3.704667000	0.205289000
6	1.827282000	3.302873000	0.247321000
1	-1.404793000	4.397934000	0.243454000
1	0.920097000	5.248999000	0.406767000
1	2.844143000	3.684938000	0.318196000
6	-2.069258000	1.629850000	-0.049371000
7	-2.011477000	0.274042000	-0.197121000
6	-3.150656000	-0.421188000	-0.293456000

6	-4.404155000	0.183393000	-0.257680000
6	-4.472680000	1.563102000	-0.111185000
6	-3.288923000	2.293425000	-0.004522000
1	-3.057809000	-1.501272000	-0.407416000
1	-5.301405000	-0.428010000	-0.343493000
1	-5.435938000	2.072721000	-0.078142000
1	-3.322006000	3.375275000	0.114920000
6	-0.891566000	-3.281764000	0.764294000
8	-1.030204000	-4.466407000	0.665786000
8	-1.137523000	-2.523633000	1.819781000
1	-1.449494000	-3.089011000	2.554241000
7	-0.227805000	-3.162666000	-1.461109000
1	0.096581000	-2.785111000	-2.352037000
1	-0.448065000	-4.170372000	-1.401910000
j(NH_2/CN)			
46	-0.014090000	-0.641700000	-0.137484000
6	-0.205707000	-2.663876000	-0.263635000
6	0.356502000	4.055437000	0.154460000
6	4.380616000	-1.096953000	0.045319000
6	3.019983000	-1.377651000	-0.043276000
7	2.084612000	-0.419590000	-0.051311000
6	2.469640000	0.887283000	0.041663000
6	3.812018000	1.233326000	0.129325000
6	4.781787000	0.230464000	0.129379000
1	2.667480000	-2.407201000	-0.103473000
1	4.105985000	2.279263000	0.203405000
1	5.838456000	0.489822000	0.200148000
1	5.101040000	-1.913842000	0.050877000
6	1.373750000	1.889568000	0.060444000
7	0.143589000	1.354776000	0.006156000
6	-0.987826000	2.077173000	0.023275000
6	-0.906921000	3.466733000	0.099131000
6	1.512365000	3.273975000	0.136615000
1	-1.802055000	4.086069000	0.114639000
1	0.442091000	5.140818000	0.213255000
1	2.493339000	3.743825000	0.181455000
6	-2.227571000	1.261738000	-0.037321000
7	-2.051564000	-0.090161000	-0.123191000
6	-3.125940000	-0.887632000	-0.160154000
6	-4.427407000	-0.393713000	-0.122368000
6	-4.615984000	0.979763000	-0.041971000
6	-3.499881000	1.816385000	0.002250000
1	-2.942874000	-1.960739000	-0.216049000
1	-5.267330000	-1.086446000	-0.153243000
1	-5.620059000	1.403531000	-0.009637000

1	-3.627297000	2.895574000	0.072449000
6	-0.762101000	-3.318922000	0.889645000
7	-1.244840000	-3.746792000	1.853191000
7	0.091223000	-3.424854000	-1.269878000
1	0.467506000	-3.024083000	-2.130622000
1	-0.053231000	-4.440640000	-1.260388000
Pt carbenoids			
a(H/H)			
78	-0.000013000	0.899495000	-0.000114000
6	0.000043000	2.766573000	-0.000231000
6	0.000006000	-3.845683000	-0.000294000
6	4.389344000	1.016093000	0.014622000
6	3.050449000	1.394922000	0.018908000
7	2.050194000	0.502105000	0.009329000
6	2.345982000	-0.836614000	-0.004330000
6	3.663016000	-1.271239000	-0.011792000
6	4.700563000	-0.336938000	-0.002784000
1	2.771859000	2.447541000	0.030297000
1	3.885277000	-2.337321000	-0.024462000
1	5.738664000	-0.670471000	-0.008282000
1	5.163088000	1.782709000	0.025155000
6	1.186998000	-1.768859000	-0.006348000
7	0.000006000	-1.143508000	0.000090000
6	-1.186986000	-1.768861000	0.006340000
6	-1.217875000	-3.161154000	0.005715000
6	1.217888000	-3.161149000	-0.006112000
1	-2.156606000	-3.712525000	0.008725000
1	0.000009000	-4.936253000	-0.000458000
1	2.156618000	-3.712522000	-0.009333000
6	-2.345976000	-0.836625000	0.004509000
7	-2.050192000	0.502093000	-0.009348000
6	-3.050441000	1.394921000	-0.018783000
6	-4.389335000	1.016096000	-0.014109000
6	-4.700552000	-0.336932000	0.003559000
6	-3.663010000	-1.271242000	0.012384000
1	-2.771831000	2.447532000	-0.030399000
1	-5.163083000	1.782710000	-0.024541000
1	-5.738654000	-0.670460000	0.009403000
1	-3.885280000	-2.337319000	0.025271000
1	0.739980000	3.385424000	-0.537049000
1	-0.739771000	3.385643000	0.536504000
b(OH/Cl)			
78	0.009117000	-0.503848000	-0.080359000
6	0.142954000	-2.447500000	-0.124532000
6	-0.379582000	4.198553000	0.248427000

6	4.397449000	-0.238207000	-0.273326000
6	3.097649000	-0.735098000	-0.246040000
7	2.024739000	0.058259000	-0.120628000
6	2.207011000	1.412223000	-0.029305000
6	3.479373000	1.965013000	-0.045888000
6	4.592377000	1.132129000	-0.166299000
1	2.911783000	-1.804155000	-0.327476000
1	3.607016000	3.043749000	0.030063000
1	5.596616000	1.556460000	-0.181098000
1	5.232736000	-0.929168000	-0.378488000
6	0.973207000	2.231934000	0.067823000
7	-0.155292000	1.506844000	0.029823000
6	-1.387977000	2.033349000	0.096614000
6	-1.533334000	3.414012000	0.208259000
6	0.887747000	3.617828000	0.179115000
1	-2.515957000	3.878934000	0.265619000
1	-0.469767000	5.281594000	0.336481000
1	1.779611000	4.241037000	0.213146000
6	-2.468507000	1.017488000	0.040714000
7	-2.062725000	-0.287028000	-0.048569000
6	-2.986757000	-1.255230000	-0.117208000
6	-4.352246000	-0.986930000	-0.092867000
6	-4.773982000	0.332269000	0.003660000
6	-3.816189000	1.345078000	0.068971000
1	-2.620507000	-2.276024000	-0.202385000
1	-5.061153000	-1.811745000	-0.150200000
1	-5.835753000	0.578930000	0.025871000
1	-4.123245000	2.387496000	0.138966000
8	-0.475180000	-3.157362000	-0.995385000
1	-0.332778000	-4.126905000	-0.903251000
17	1.047030000	-3.331128000	1.025338000
c(OH/COOH)			
78	-0.029385000	-0.403702000	-0.140757000
6	-0.457967000	-2.286987000	-0.227166000
6	1.060055000	4.173603000	0.411290000
6	4.208199000	-1.534186000	-0.160520000
6	2.817917000	-1.591223000	-0.200142000
7	2.050770000	-0.499185000	-0.079484000
6	2.647804000	0.723549000	0.075667000
6	4.029151000	0.840574000	0.124531000
6	4.823325000	-0.301221000	0.008497000
1	2.302080000	-2.538668000	-0.342893000
1	4.489832000	1.819639000	0.247189000
1	5.909914000	-0.219688000	0.045541000
1	4.784673000	-2.452551000	-0.263105000
6	1.733497000	1.890169000	0.168744000
7	0.438248000	1.562496000	0.052814000
6	-0.568586000	2.446951000	0.105164000
6	-0.278839000	3.797078000	0.287748000
6	2.083335000	3.226070000	0.353172000
1	-1.066323000	4.547150000	0.336044000
1	1.310227000	5.224941000	0.556081000
1	3.122918000	3.533474000	0.452830000
6	-1.908426000	1.826752000	-0.061113000
7	-1.929647000	0.465530000	-0.206657000
6	-3.104320000	-0.148983000	-0.403932000
6	-4.311666000	0.540011000	-0.455401000
6	-4.301507000	1.918942000	-0.290659000
6	-3.081818000	2.566861000	-0.093810000
1	-3.077406000	-1.229681000	-0.529687000
1	-5.236661000	-0.009447000	-0.624768000
1	-5.228700000	2.491717000	-0.321719000
1	-3.048090000	3.648695000	0.025718000
6	-1.383449000	-3.070156000	0.743553000
8	-1.483812000	-4.255888000	0.591024000
8	-1.949948000	-2.305260000	1.647808000
1	-2.485695000	-2.855216000	2.255326000
8	0.042570000	-3.096799000	-1.070362000
1	-0.311023000	-4.016746000	-0.885705000
d(OH/CN)			
78	-0.012833000	-0.554997000	-0.107703000
6	-0.151438000	-2.488346000	-0.231394000
6	0.321912000	4.154113000	0.177090000
6	4.346560000	-1.009439000	0.057233000
6	2.985420000	-1.285523000	-0.019894000
7	2.054298000	-0.318748000	-0.026604000
6	2.447761000	0.990398000	0.057249000
6	3.792314000	1.324774000	0.133289000
6	4.756524000	0.315927000	0.131206000
1	2.629681000	-2.314450000	-0.070941000
1	4.091537000	2.369852000	0.199713000
1	5.815329000	0.569192000	0.192739000
1	5.061002000	-1.831747000	0.061754000
6	1.357354000	1.998877000	0.079753000
7	0.130182000	1.457790000	0.029426000
6	-1.008148000	2.167919000	0.048641000
6	-0.939000000	3.557394000	0.124390000
6	1.486323000	3.383307000	0.155920000
1	-1.837956000	4.171263000	0.141595000
1	0.399089000	5.240213000	0.236053000

1	2.462902000	3.862624000	0.198567000
6	-2.230634000	1.326957000	-0.016178000
7	-2.027254000	-0.025226000	-0.103642000
6	-3.087504000	-0.845562000	-0.152299000
6	-4.396652000	-0.374322000	-0.122854000
6	-4.613409000	0.994506000	-0.038287000
6	-3.513605000	1.852695000	0.016214000
1	-2.885074000	-1.914468000	-0.211704000
1	-5.221535000	-1.084506000	-0.164425000
1	-5.625907000	1.398360000	-0.012031000
1	-3.660666000	2.929389000	0.087789000
6	-0.720803000	-3.243271000	0.860258000
7	-1.227515000	-3.732796000	1.781009000
8	0.213366000	-3.245737000	-1.202781000
1	0.559779000	-2.729596000	-1.959345000
e(CH_3/Cl)			
78	-0.006937000	-0.512644000	0.078453000
6	-0.060841000	-2.426329000	0.211128000
6	0.204442000	4.227835000	-0.111503000
6	-4.395775000	-0.424447000	0.090683000
6	-3.075536000	-0.863063000	0.108990000
7	-2.035715000	-0.017301000	0.073084000
6	-2.271780000	1.329995000	0.011987000
6	-3.567937000	1.824935000	-0.009859000
6	-4.646174000	0.940231000	0.030645000
1	-2.842143000	-1.926106000	0.148958000
1	-3.740589000	2.898928000	-0.060729000
1	-5.668131000	1.319827000	0.013979000
1	-5.203593000	-1.154288000	0.122931000
6	-1.071345000	2.204738000	-0.032203000
7	0.083203000	1.524535000	-0.021658000
6	1.294159000	2.098319000	-0.051041000
6	1.388224000	3.487552000	-0.097990000
6	-1.040499000	3.597095000	-0.078692000
1	2.351937000	3.993308000	-0.123470000
1	0.253157000	5.316493000	-0.147697000
1	-1.955108000	4.187486000	-0.088154000
6	2.411451000	1.118287000	-0.037153000
7	2.055478000	-0.202708000	0.016730000
6	3.014987000	-1.140335000	0.006683000
6	4.368299000	-0.823783000	-0.046602000
6	4.740181000	0.513905000	-0.090819000
6	3.746694000	1.493282000	-0.087971000
1	2.686461000	-2.178731000	0.038884000
1	5.106759000	-1.624348000	-0.054331000

1	5.791705000	0.799232000	-0.131775000
1	4.015422000	2.547790000	-0.129331000
17	-0.599130000	-3.342742000	-1.099805000
6	0.320404000	-3.206723000	1.403571000
1	-0.632640000	-3.367097000	1.952569000
1	0.713761000	-4.205264000	1.165349000
1	0.974811000	-2.635012000	2.073930000
f(CH_3/COOH)			
78	0.008229000	-0.408609000	0.148182000
6	0.083939000	-2.287322000	0.452890000
6	-0.246128000	4.325545000	-0.207967000
6	-4.353653000	-0.769350000	-0.004690000
6	-2.997404000	-1.067662000	0.067570000
7	-2.051669000	-0.114591000	0.058269000
6	-2.423067000	1.198639000	-0.047636000
6	-3.762709000	1.553845000	-0.119874000
6	-4.742730000	0.561488000	-0.093233000
1	-2.651588000	-2.099517000	0.121399000
1	-4.044415000	2.602501000	-0.203119000
1	-5.797704000	0.831140000	-0.149182000
1	-5.081750000	-1.579267000	0.007215000
6	-1.316325000	2.188533000	-0.097349000
7	-0.100264000	1.625390000	-0.066577000
6	1.047848000	2.315502000	-0.089553000
6	1.004034000	3.706691000	-0.164732000
6	-1.423453000	3.575160000	-0.173156000
1	1.913739000	4.304355000	-0.185937000
1	-0.304716000	5.412742000	-0.265959000
1	-2.392051000	4.071437000	-0.201227000
6	2.254230000	1.451554000	-0.031189000
7	2.027426000	0.104130000	0.070636000
6	3.070318000	-0.740883000	0.084587000
6	4.386812000	-0.291564000	0.023562000
6	4.629530000	1.072414000	-0.059516000
6	3.545950000	1.951945000	-0.093058000
1	2.839027000	-1.805124000	0.119020000
1	5.197999000	-1.018218000	0.038169000
1	5.648804000	1.456519000	-0.106976000
1	3.711288000	3.025237000	-0.174262000
6	0.392115000	-3.194633000	-0.700288000
8	1.547707000	-3.427912000	-0.929704000
8	-0.680363000	-3.625076000	-1.333823000
1	-0.416473000	-4.210734000	-2.073192000
6	-0.029124000	-2.956999000	1.753004000
1	-0.743192000	-3.799534000	1.679514000

1	0.946670000	-3.463205000	1.918471000
1	-0.255834000	-2.294849000	2.593556000
g(CH_3/CN)			
78	0.013408000	-0.566620000	-0.078949000
6	0.139724000	-2.471538000	-0.209053000
6	-0.340593000	4.166739000	0.103723000
6	4.400217000	-0.339127000	-0.111422000
6	3.094592000	-0.819998000	-0.131393000
7	2.027621000	-0.007844000	-0.092119000
6	2.221938000	1.346603000	-0.027109000
6	3.501505000	1.881901000	-0.004666000
6	4.607742000	1.032110000	-0.047278000
1	2.900785000	-1.890633000	-0.172983000
1	3.640049000	2.960633000	0.050100000
1	5.617195000	1.443852000	-0.028517000
1	5.229787000	-1.044217000	-0.144554000
6	0.995992000	2.182986000	0.022672000
7	-0.137745000	1.467261000	0.021149000
6	-1.365712000	2.005208000	0.057239000
6	-1.501463000	3.390593000	0.100564000
6	0.922519000	3.573815000	0.065109000
1	-2.480014000	3.866728000	0.131543000
1	-0.422263000	5.253586000	0.136673000
1	1.818883000	4.191660000	0.066503000
6	-2.452645000	0.993113000	0.059568000
7	-2.057527000	-0.316944000	0.010815000
6	-2.988225000	-1.283162000	0.043556000
6	-4.349552000	-1.006614000	0.112538000
6	-4.761326000	0.319520000	0.148663000
6	-3.797746000	1.328200000	0.124286000
1	-2.630024000	-2.311369000	0.018060000
1	-5.062947000	-1.829274000	0.139582000
1	-5.820391000	0.573529000	0.201243000
1	-4.097419000	2.374516000	0.160693000
6	0.830082000	-3.161843000	0.833593000
7	1.428109000	-3.674909000	1.689236000
6	-0.369924000	-3.307923000	-1.306192000
1	0.539454000	-3.610071000	-1.874836000
1	-0.786812000	-4.262802000	-0.944032000
1	-1.038069000	-2.776182000	-1.991443000
h(NH_2/Cl)			
78	-0.004451000	-0.490903000	0.111244000
6	-0.067628000	-2.461891000	0.237927000
6	0.133341000	4.221747000	-0.172700000
6	-4.387379000	-0.508901000	0.071300000
6	-3.058645000	-0.920623000	0.112864000
7	-2.037356000	-0.053579000	0.081256000
6	-2.298967000	1.287523000	-0.003368000
6	-3.603939000	1.756835000	-0.046796000
6	-4.663972000	0.849850000	-0.007585000
1	-2.800103000	-1.977660000	0.168729000
1	-3.798274000	2.826188000	-0.114919000
1	-5.693141000	1.208024000	-0.042137000
1	-5.181477000	-1.253746000	0.099270000
6	-1.112145000	2.181071000	-0.054873000
7	0.054795000	1.520155000	-0.026403000
6	1.258136000	2.111723000	-0.065582000
6	1.328660000	3.501035000	-0.141200000
6	-1.101660000	3.572229000	-0.130198000
1	2.284557000	4.020952000	-0.174997000
1	0.164840000	5.310022000	-0.231376000
1	-2.025737000	4.147231000	-0.155244000
6	2.390350000	1.149229000	-0.026532000
7	2.050127000	-0.173856000	0.061276000
6	3.018240000	-1.100530000	0.079003000
6	4.368502000	-0.769261000	0.019540000
6	4.724768000	0.570879000	-0.061354000
6	3.720588000	1.539576000	-0.085925000
1	2.697170000	-2.139996000	0.139591000
1	5.117083000	-1.560181000	0.035220000
1	5.773024000	0.866792000	-0.109384000
1	3.977679000	2.595523000	-0.155811000
17	-0.297749000	-3.390289000	-1.184332000
7	0.049946000	-3.166346000	1.327061000
1	0.177106000	-2.696083000	2.223532000
1	0.004084000	-4.189316000	1.324687000
i(NH_2/COOH)			
78	0.025957000	-0.391109000	-0.171818000
6	0.364912000	-2.322027000	-0.367025000
6	-0.763388000	4.249110000	0.307726000
6	4.367285000	0.220036000	-0.241123000
6	3.109600000	-0.374657000	-0.265585000
7	1.976550000	0.333376000	-0.166104000
6	2.048864000	1.693229000	-0.027515000
6	3.275116000	2.342183000	0.005626000
6	4.451268000	1.599551000	-0.103390000
1	3.003308000	-1.453524000	-0.371070000
1	3.317397000	3.424563000	0.117515000
1	5.419816000	2.099509000	-0.079421000
1	5.257380000	-0.401333000	-0.329224000

6	0.750552000	2.408620000	0.085611000
7	-0.312483000	1.592556000	0.047507000
6	-1.584500000	2.008260000	0.128338000
6	-1.847037000	3.369958000	0.263146000
6	0.548752000	3.780827000	0.219434000
1	-2.865052000	3.749583000	0.331678000
1	-0.944918000	5.319093000	0.412162000
1	1.383960000	4.478272000	0.253812000
6	-2.572283000	0.899220000	0.063023000
7	-2.054989000	-0.359954000	-0.082000000
6	-2.889482000	-1.407894000	-0.129329000
6	-4.271264000	-1.267772000	-0.044220000
6	-4.806114000	0.006426000	0.095928000
6	-3.942617000	1.100872000	0.151210000
1	-2.432026000	-2.390805000	-0.236127000
1	-4.904713000	-2.152767000	-0.086081000
1	-5.884050000	0.154141000	0.165752000
1	-4.339711000	2.108301000	0.266511000
6	0.903728000	-3.203333000	0.779271000
8	0.969534000	-4.393808000	0.670799000
8	1.263185000	-2.470637000	1.820068000
1	1.587593000	-3.063697000	2.526422000
7	0.169846000	-3.045980000	-1.421114000
1	-0.173921000	-2.649935000	-2.295568000
1	0.375691000	-4.054998000	-1.376319000
j(NH_2/CN)			
78	-0.011890000	-0.545880000	-0.112970000
6	-0.167342000	-2.515051000	-0.236347000
6	0.308666000	4.158693000	0.172085000
6	4.348983000	-0.990707000	0.048543000
6	2.988305000	-1.270427000	-0.030510000
7	2.054991000	-0.308027000	-0.035208000
6	2.444685000	1.001329000	0.052056000
6	3.787906000	1.341178000	0.129784000
6	4.755065000	0.335451000	0.126242000
1	2.630619000	-2.297890000	-0.086500000
1	4.083537000	2.387037000	0.198962000
1	5.812943000	0.591741000	0.189430000
1	5.066631000	-1.810027000	0.051784000
6	1.350614000	2.006177000	0.075488000
7	0.125533000	1.461248000	0.025568000
6	-1.014829000	2.167891000	0.044911000
6	-0.950099000	3.557631000	0.120013000
6	1.474933000	3.391401000	0.151124000
1	-1.851254000	4.168210000	0.137578000
1	0.382342000	5.244962000	0.230624000
1	2.450220000	3.873319000	0.193346000
6	-2.235462000	1.323110000	-0.014861000
7	-2.027548000	-0.027659000	-0.100546000
6	-3.084445000	-0.851247000	-0.137938000
6	-4.395385000	-0.385569000	-0.100655000
6	-4.616690000	0.982953000	-0.020446000
6	-3.520136000	1.845270000	0.023865000
1	-2.874208000	-1.918867000	-0.193790000
1	-5.218464000	-1.098188000	-0.131876000
1	-5.630450000	1.382976000	0.011523000
1	-3.670591000	2.921577000	0.093411000
6	-0.640741000	-3.227099000	0.927813000
7	-1.053495000	-3.723909000	1.889949000
7	0.094647000	-3.256963000	-1.273575000
1	0.407651000	-2.830686000	-2.146475000
1	-0.024592000	-4.274309000	-1.266630000
Cu carbenoids			
a(H/H)			
29	-0.000291000	1.749790000	-0.000037000
6	-0.000955000	3.656498000	-0.000108000
1	-0.000965000	4.313640000	-0.888754000
1	-0.001488000	4.312859000	0.889132000
6	-3.492661000	-0.872775000	-0.000049000
6	0.746826000	-1.047085000	0.000033000
6	1.504786000	-2.217530000	-0.000367000
6	2.894056000	-2.126481000	-0.000359000
6	3.493021000	-0.871631000	0.000049000
6	2.669503000	0.249480000	0.000400000
7	1.334083000	0.166446000	0.000385000
1	1.026308000	-3.194364000	-0.000745000
1	3.500731000	-3.032140000	-0.000684000
1	4.575442000	-0.754453000	0.000079000
1	3.091037000	1.255448000	0.000723000
6	-0.746391000	-1.047349000	0.000031000
6	-1.503983000	-2.218029000	0.000310000
6	-2.893280000	-2.127431000	0.000262000
6	-2.669502000	0.248600000	-0.000262000
7	-1.334060000	0.165985000	-0.000216000
1	-1.025203000	-3.194716000	0.000613000
1	-3.499647000	-3.033295000	0.000481000
1	-4.575120000	-0.755944000	-0.000104000
1	-3.091355000	1.254435000	-0.000478000
b(OH/Cl)			
29	-0.969771000	-0.000740000	0.199567000

6	-2.967064000	-0.000649000	0.343256000
6	1.682012000	3.495959000	-0.020464000
6	1.839444000	-0.747401000	-0.034068000
6	3.011591000	-1.498409000	-0.131423000
6	2.931215000	-2.887997000	-0.124476000
6	1.684637000	-3.495146000	-0.020624000
6	0.563151000	-2.677361000	0.072415000
7	0.634865000	-1.341750000	0.066112000
1	3.982416000	-1.014852000	-0.212172000
1	3.838213000	-3.487996000	-0.199779000
1	1.574686000	-4.578339000	-0.011236000
1	-0.436655000	-3.106129000	0.155588000
6	1.838887000	0.748339000	-0.034022000
6	3.010492000	1.500227000	-0.131118000
6	2.929064000	2.889756000	-0.124114000
6	0.561127000	2.677320000	0.072350000
7	0.633848000	1.341772000	0.065999000
1	3.981700000	1.017407000	-0.211675000
1	3.835625000	3.490441000	-0.199206000
1	1.571249000	4.579069000	-0.011046000
1	-0.439018000	3.105329000	0.155376000
8	-3.637392000	-0.000712000	1.433298000
1	-4.613235000	-0.000564000	1.306508000
17	-3.968284000	-0.000305000	-1.068073000
c(OH/COOH)			
29	0.777327000	-0.000161000	-0.312351000
6	2.749437000	-0.000277000	-0.546758000
6	-1.865874000	3.494547000	0.015374000
6	-2.023880000	-0.747527000	0.047380000
6	-3.189605000	-1.500203000	0.195617000
6	-3.107743000	-2.889607000	0.179670000
6	-1.866436000	-3.494394000	0.015737000
6	-0.751332000	-2.674540000	-0.124871000
7	-0.824296000	-1.339298000	-0.108987000
1	-4.156308000	-1.017997000	0.321715000
1	-4.009513000	-3.491371000	0.293639000
1	-1.755942000	-4.577381000	-0.004564000
1	0.244083000	-3.101074000	-0.257419000
6	-2.023758000	0.747709000	0.047315000
6	-3.189332000	1.500589000	0.195707000
6	-3.107247000	2.889977000	0.179607000
6	-0.750927000	2.674497000	-0.125345000
7	-0.824104000	1.339269000	-0.109312000
1	-4.156084000	1.018552000	0.322075000
1	-4.008896000	3.491900000	0.293700000
1	-1.755208000	4.577514000	-0.005061000
1	0.244534000	3.100856000	-0.258115000
8	3.416189000	-0.000515000	-1.629421000
1	4.395056000	-0.000456000	-1.412464000
6	3.763743000	-0.000064000	0.625244000
8	4.946143000	-0.000324000	0.398006000
8	3.172680000	0.000729000	1.801695000
1	3.852232000	0.000965000	2.502458000
d(OH/CN)			
29	-1.082798000	0.000280000	-0.247386000
6	-3.075422000	0.000246000	-0.359647000
6	1.553670000	-3.496013000	0.033301000
6	1.713198000	0.747500000	0.031639000
6	2.881744000	1.499386000	0.156807000
6	2.799659000	2.888967000	0.156973000
6	1.554748000	3.495689000	0.033333000
6	0.436349000	2.677880000	-0.088311000
7	0.509961000	1.341830000	-0.091184000
1	3.851182000	1.016539000	0.255660000
1	3.704132000	3.489462000	0.254989000
1	1.443480000	4.578753000	0.031680000
1	-0.562043000	3.106972000	-0.185272000
6	1.712973000	-0.747876000	0.031629000
6	2.881297000	-1.500123000	0.156697000
6	2.798778000	-2.889679000	0.156851000
6	0.435517000	-2.677855000	-0.088254000
7	0.509546000	-1.341831000	-0.091121000
1	3.850895000	-1.017579000	0.255461000
1	3.703074000	-3.490455000	0.254780000
1	1.442073000	-4.579042000	0.031638000
1	-0.563016000	-3.106634000	-0.185157000
8	-3.907840000	0.000249000	-1.342547000
1	-3.433117000	0.000305000	-2.193976000
6	-3.783699000	0.000119000	0.904058000
7	-4.194603000	0.000306000	1.988633000
e(CH_3/Cl)			
29	-0.982934000	-0.023753000	0.177943000
6	-2.958435000	-0.016617000	0.294663000
6	1.625209000	3.507756000	-0.001130000
6	1.843381000	-0.731360000	-0.022683000
6	3.024644000	-1.468896000	-0.110497000
6	2.958799000	-2.859238000	-0.114781000
6	1.717329000	-3.480114000	-0.032141000
6	0.586092000	-2.674951000	0.052942000
7	0.643829000	-1.338685000	0.058674000

1	3.990966000	-0.974024000	-0.176094000
1	3.872823000	-3.449333000	-0.183203000
1	1.618715000	-4.564445000	-0.033613000
1	-0.410397000	-3.114501000	0.118117000
6	1.824045000	0.763474000	-0.016018000
6	2.985558000	1.532582000	-0.096106000
6	2.882697000	2.920736000	-0.088154000
6	0.515548000	2.672230000	0.075996000
7	0.609020000	1.338059000	0.069798000
1	3.964753000	1.064118000	-0.164846000
1	3.780797000	3.535435000	-0.150361000
1	1.498088000	4.589086000	0.006977000
1	-0.492339000	3.084446000	0.144702000
6	-3.819900000	-0.074211000	1.486897000
1	-3.809593000	-1.152553000	1.757204000
1	-3.338740000	0.437992000	2.332106000
1	-4.862729000	0.237266000	1.340460000
17	-3.834530000	0.026553000	-1.154414000
f(CH_3/COOH)			
29	0.695159000	-0.603885000	-0.211019000
6	2.632821000	-0.779622000	-0.441610000
6	-0.525597000	3.615859000	-0.144477000
6	-2.173391000	-0.290260000	0.059676000
6	-3.533671000	-0.583169000	0.160125000
6	-3.939893000	-1.911633000	0.245928000
6	-2.979789000	-2.917361000	0.230192000
6	-1.643503000	-2.546660000	0.123608000
7	-1.247364000	-1.271104000	0.039371000
1	-4.276329000	0.211474000	0.169683000
1	-4.999574000	-2.155067000	0.324527000
1	-3.251830000	-3.969449000	0.297183000
1	-0.853377000	-3.298604000	0.105191000
6	-1.654868000	1.110108000	-0.022780000
6	-2.481277000	2.231780000	0.042370000
6	-1.905890000	3.498654000	-0.023730000
6	0.232338000	2.449212000	-0.202118000
7	-0.321267000	1.233560000	-0.150115000
1	-3.559260000	2.132221000	0.149131000
1	-2.536564000	4.386505000	0.025187000
1	-0.037158000	4.587875000	-0.190674000
1	1.321422000	2.474913000	-0.279928000
6	3.435593000	-0.068405000	0.575537000
8	3.341160000	1.135454000	0.586511000
6	3.416257000	-1.234085000	-1.587882000
1	3.756533000	-2.216364000	-1.168361000
1	2.835823000	-1.453043000	-2.489433000
1	4.337807000	-0.668022000	-1.792869000
8	4.179671000	-0.818834000	1.370098000
1	4.641884000	-0.236435000	2.003210000
g(CH_3/CN)			
29	1.075187000	-0.031586000	-0.188901000
6	3.031134000	-0.029248000	-0.308543000
6	-1.490581000	3.513060000	0.000193000
6	-1.734757000	-0.724511000	0.030017000
6	-2.917575000	-1.457046000	0.130653000
6	-2.856963000	-2.847792000	0.139601000
6	-1.618921000	-3.474340000	0.048770000
6	-0.484735000	-2.675051000	-0.049323000
7	-0.538171000	-1.338300000	-0.059864000
1	-3.881175000	-0.957835000	0.203244000
1	-3.772706000	-3.433901000	0.218476000
1	-1.524670000	-4.559023000	0.054245000
1	0.509110000	-3.119217000	-0.120750000
6	-1.707709000	0.769537000	0.019541000
6	-2.862910000	1.546258000	0.108550000
6	-2.750852000	2.933896000	0.098288000
6	-0.386419000	2.671344000	-0.085790000
7	-0.489529000	1.337521000	-0.077785000
1	-3.844347000	1.083964000	0.186600000
1	-3.644400000	3.554397000	0.167828000
1	-1.356609000	4.593527000	-0.009264000
1	0.623187000	3.077386000	-0.162753000
6	3.723454000	0.018349000	0.927516000
7	4.217105000	0.069706000	1.982265000
6	3.892940000	-0.103568000	-1.495319000
1	4.126446000	-1.195078000	-1.536367000
1	3.376028000	0.144965000	-2.427553000
1	4.866252000	0.398998000	-1.392807000
h(NH_2/Cl)			
29	0.985547000	-0.046318000	0.234920000
6	2.997277000	-0.072784000	0.345422000
6	-1.772897000	-3.465880000	-0.051582000
6	-1.817462000	0.779682000	-0.016948000
6	-2.967441000	1.562778000	-0.128803000
6	-2.848667000	2.949527000	-0.117045000
6	-1.587105000	3.521392000	0.004999000
6	-0.490407000	2.672070000	0.112294000
7	-0.598990000	1.339275000	0.103305000
1	-3.949823000	1.106464000	-0.226833000
1	-3.737367000	3.574730000	-0.204432000

1	-1.447300000	4.601134000	0.016363000
1	0.520383000	3.071933000	0.207962000
6	-1.856760000	-0.715661000	-0.028682000
6	-3.047829000	-1.434800000	-0.140199000
6	-3.003388000	-2.825836000	-0.151135000
6	-0.631508000	-2.677945000	0.057877000
7	-0.668570000	-1.341166000	0.070917000
1	-4.005517000	-0.925328000	-0.218762000
1	-3.925085000	-3.401404000	-0.238095000
1	-1.690504000	-4.551554000	-0.058458000
1	0.357196000	-3.132825000	0.136919000
17	3.842467000	0.042072000	-1.163299000
7	3.818261000	-0.154389000	1.352709000
1	4.836452000	-0.149820000	1.247930000
1	3.440981000	-0.226614000	2.295099000
i(NH_2/COOH)			
29	-0.807128000	0.000677000	-0.351458000
6	-2.800647000	0.001487000	-0.578848000
6	1.849352000	-3.494658000	0.017181000
6	2.008583000	0.747368000	0.035297000
6	3.173367000	1.498962000	0.199089000
6	3.091920000	2.888323000	0.190433000
6	1.851957000	3.493922000	0.019057000
6	0.738717000	2.674267000	-0.138193000
7	0.811209000	1.339250000	-0.131395000
1	4.138664000	1.016040000	0.333101000
1	3.992448000	3.489384000	0.317250000
1	1.741301000	4.577043000	0.006562000
1	-0.256151000	3.101091000	-0.275290000
6	2.008023000	-0.748231000	0.034937000
6	3.172132000	-1.500780000	0.199139000
6	3.089650000	-2.890075000	0.189711000
6	0.736820000	-2.674091000	-0.140316000
7	0.810305000	-1.339132000	-0.132763000
1	4.137680000	-1.018646000	0.334177000
1	3.989638000	-3.491875000	0.316858000
1	1.737902000	-4.577689000	0.004030000
1	-0.258280000	-3.100099000	-0.278263000
7	-3.591951000	0.003456000	-1.600607000
1	-4.612644000	0.003374000	-1.457115000
1	-3.236191000	0.005104000	-2.553322000
6	-3.668255000	-0.000657000	0.689435000
8	-4.869697000	-0.000148000	0.676904000
8	-2.904931000	-0.002361000	1.777633000
1	-3.487289000	-0.003325000	2.559841000

j(NH_2/CN)			
29	1.085051000	-0.000942000	-0.240593000
6	3.098945000	-0.001903000	-0.345273000
6	-1.571533000	3.495946000	0.029565000
6	-1.730397000	-0.747202000	0.028561000
6	-2.900887000	-1.497660000	0.148930000
6	-2.820433000	-2.887125000	0.149987000
6	-1.575419000	-3.494765000	0.031936000
6	-0.456061000	-2.677282000	-0.085134000
7	-0.527731000	-1.341698000	-0.088858000
1	-3.870278000	-1.013819000	0.243347000
1	-3.725884000	-3.486731000	0.244325000
1	-1.465171000	-4.577951000	0.031237000
1	0.542738000	-3.106601000	-0.177759000
6	-1.729573000	0.748558000	0.028081000
6	-2.899168000	1.500394000	0.148526000
6	-2.817161000	2.889772000	0.148622000
6	-0.453135000	2.677142000	-0.087451000
7	-0.526299000	1.341637000	-0.090247000
1	-3.869041000	1.017694000	0.243817000
1	-3.721896000	3.490446000	0.243017000
1	-1.460088000	4.579008000	0.028080000
1	0.546100000	3.105282000	-0.180826000
7	3.904777000	-0.004716000	-1.367312000
1	4.924563000	-0.004809000	-1.283454000
1	3.527809000	-0.006921000	-2.312682000
6	3.779504000	0.001153000	0.927800000
7	4.228765000	0.003284000	1.996873000
k(OH/COCl)			
29	0.607539000	-0.000221000	-0.490541000
6	2.561406000	-0.000794000	-0.841670000
6	-2.001533000	3.495071000	0.006130000
6	-2.157944000	-0.747464000	0.044574000
6	-3.311252000	-1.500079000	0.268857000
6	-3.230448000	-2.889565000	0.249971000
6	-2.002359000	-3.494862000	0.007342000
6	-0.898652000	-2.675544000	-0.206562000
7	-0.970781000	-1.340030000	-0.188395000
1	-4.267834000	-1.017825000	0.456729000
1	-4.122848000	-3.491114000	0.423347000
1	-1.893181000	-4.577880000	-0.017543000
1	0.086122000	-3.102783000	-0.401673000
6	-2.157767000	0.747719000	0.044335000
6	-3.310846000	1.500688000	0.268617000
6	-3.229712000	2.890148000	0.249232000

6	-0.898060000	2.675419000	-0.207696000
7	-0.970509000	1.339925000	-0.189052000
1	-4.267493000	1.018730000	0.456908000
1	-4.121931000	3.491970000	0.422599000
1	-1.892107000	4.578055000	-0.019165000
1	0.086778000	3.102353000	-0.403145000
8	3.168860000	-0.001845000	-1.959448000
1	4.157142000	-0.001845000	-1.826843000
6	3.636000000	-0.000012000	0.293208000
8	4.797348000	-0.000262000	0.034624000
17	2.969594000	0.001176000	1.889246000
l(CH_3/COCl)			
29	0.546587000	-0.455151000	0.326821000
6	2.469021000	-0.808518000	0.572531000
6	-2.917817000	-3.069392000	-0.215419000
6	-1.919161000	1.052302000	-0.001536000
6	-2.829309000	2.102706000	-0.121408000
6	-2.365754000	3.413471000	-0.045686000
6	-1.008031000	3.645054000	0.145757000
6	-0.163994000	2.544555000	0.256456000
7	-0.606134000	1.284595000	0.186952000
1	-3.889328000	1.912721000	-0.274217000
1	-3.064117000	4.245378000	-0.138028000
1	-0.603313000	4.653896000	0.207816000
1	0.910201000	2.670246000	0.403542000
6	-2.325153000	-0.384623000	-0.078670000
6	-3.647907000	-0.783495000	-0.271327000
6	-3.945952000	-2.141498000	-0.339866000
6	-1.624790000	-2.593879000	-0.024080000
7	-1.333447000	-1.289662000	0.044108000
1	-4.444999000	-0.049943000	-0.369019000
1	-4.974995000	-2.468047000	-0.490657000
1	-3.104678000	-4.140839000	-0.265240000
1	-0.784511000	-3.282110000	0.077566000
6	3.215556000	-1.106756000	1.799221000
1	4.261450000	-0.768046000	1.813505000
1	2.675727000	-0.864558000	2.720156000
1	3.248048000	-2.222499000	1.725195000
6	3.290869000	-0.720663000	-0.630487000
8	3.679713000	-1.473130000	-1.442447000
17	3.570199000	1.084593000	-0.661128000
m(NH_2/COCl)			
29	-0.491796000	-0.000664000	0.426858000
6	-2.489137000	-0.001419000	0.698964000
6	2.139197000	3.495536000	-0.051266000

6	2.297105000	-0.747294000	-0.064342000
6	3.453921000	-1.498089000	-0.279147000
6	3.373448000	-2.887515000	-0.272409000
6	2.141950000	-3.494525000	-0.052433000
6	1.036098000	-2.676169000	0.154624000
7	1.107963000	-1.340924000	0.151058000
1	4.412537000	-1.014539000	-0.452588000
1	4.268030000	-3.487546000	-0.439658000
1	2.031830000	-4.577696000	-0.041313000
1	0.047878000	-3.104627000	0.329245000
6	2.296506000	0.748435000	-0.064125000
6	3.452622000	1.500207000	-0.279286000
6	3.371060000	2.889568000	-0.272065000
6	1.034083000	2.676240000	0.156023000
7	1.106988000	1.341055000	0.151978000
1	4.411511000	1.017460000	-0.453460000
1	4.265080000	3.490357000	-0.439600000
1	2.028233000	4.578616000	-0.039723000
1	0.045610000	3.103871000	0.331251000
6	-3.219747000	-0.000523000	-0.646761000
8	-2.620073000	-0.000960000	-1.666798000
17	-5.002695000	0.001247000	-0.619443000
7	-3.270351000	-0.002959000	1.727542000
1	-4.295180000	-0.003238000	1.641835000
1	-2.881877000	-0.003826000	2.669332000
Ag carbenoids			
a(H/H)			
47	1.699715000	-0.000154000	0.000088000
6	3.804015000	-0.000151000	0.000213000
1	4.456412000	-0.000487000	-0.891607000
1	4.456140000	0.000304000	0.892235000
6	-1.267376000	3.501912000	-0.000167000
6	-1.386646000	-0.748557000	-0.000090000
6	-2.571810000	-1.488591000	-0.000689000
6	-2.510571000	-2.878411000	-0.000621000
6	-1.267890000	-3.501723000	0.000030000
6	-0.133909000	-2.696483000	0.000519000
7	-0.188044000	-1.359830000	0.000452000
1	-3.541164000	-0.996253000	-0.001287000
1	-3.428928000	-3.465679000	-0.001094000
1	-1.170105000	-4.586167000	0.000120000
1	0.863284000	-3.139535000	0.000984000
6	-1.386537000	0.748766000	-0.000059000
6	-2.571593000	1.488973000	0.000385000
6	-2.510149000	2.878784000	0.000322000

6	-0.133513000	2.696504000	-0.000507000
7	-0.187846000	1.359861000	-0.000446000
1	-3.541020000	0.996778000	0.000856000
1	-3.428420000	3.466187000	0.000673000
1	-1.169431000	4.586342000	-0.000246000
1	0.863745000	3.139409000	-0.000841000
b(OH/Cl)			
47	0.979825000	-0.005758000	0.173972000
6	3.205185000	-0.015190000	0.305407000
6	-2.031632000	-3.496489000	-0.070296000
6	-2.116483000	0.753056000	-0.051008000
6	-3.285095000	1.487127000	-0.272726000
6	-3.232924000	2.877034000	-0.250673000
6	-2.015416000	3.504100000	-0.010446000
6	-0.894899000	2.702751000	0.182561000
7	-0.938934000	1.366042000	0.160907000
1	-4.228563000	0.987027000	-0.479515000
1	-4.136685000	3.461359000	-0.424212000
1	-1.925642000	4.588849000	0.019665000
1	0.083924000	3.150254000	0.363859000
6	-2.121205000	-0.744796000	-0.046288000
6	-3.313958000	-1.471814000	0.003413000
6	-3.267410000	-2.862035000	-0.010218000
6	-0.890260000	-2.701877000	-0.098811000
7	-0.928979000	-1.364897000	-0.084653000
1	-4.274476000	-0.965967000	0.070696000
1	-4.190244000	-3.440895000	0.030369000
1	-1.944781000	-4.581784000	-0.086297000
1	0.101826000	-3.155359000	-0.135289000
8	3.862031000	-0.061011000	1.399284000
1	4.840630000	-0.061632000	1.285319000
17	4.210939000	0.033978000	-1.096167000
c(OH/COOH)			
47	0.803423000	-0.052024000	-0.269860000
6	2.987780000	-0.110231000	-0.499967000
6	-2.105740000	3.528652000	-0.042340000
6	-2.310257000	-0.713813000	0.079772000
6	-3.519332000	-1.414926000	0.083916000
6	-3.501602000	-2.805443000	0.118104000
6	-2.277994000	-3.465330000	0.144589000
6	-1.119746000	-2.695206000	0.121240000
7	-1.130606000	-1.358229000	0.087623000
1	-4.470522000	-0.889027000	0.041830000
1	-4.437153000	-3.364966000	0.118798000
1	-2.213699000	-4.551924000	0.173579000
1	-0.136592000	-3.169104000	0.129453000
6	-2.271581000	0.782904000	0.056258000
6	-3.414202000	1.549310000	0.302736000
6	-3.329205000	2.936791000	0.251402000
6	-1.012173000	2.696107000	-0.257547000
7	-1.087547000	1.361768000	-0.207683000
1	-4.361792000	1.076085000	0.550282000
1	-4.212083000	3.546754000	0.443369000
1	-1.991124000	4.610068000	-0.097631000
1	-0.029711000	3.115261000	-0.481883000
8	3.647840000	-0.285703000	-1.566609000
1	4.630008000	-0.261758000	-1.352385000
6	4.007456000	0.062663000	0.657153000
8	5.187188000	0.012586000	0.421341000
8	3.425017000	0.253779000	1.819818000
1	4.108084000	0.354811000	2.510150000
d(OH/CN)			
47	1.086195000	-0.040561000	-0.205996000
6	3.304776000	-0.074378000	-0.318251000
6	-1.824437000	3.528126000	-0.012025000
6	-2.015062000	-0.718116000	0.054414000
6	-3.221979000	-1.422477000	0.036744000
6	-3.202314000	-2.813037000	0.070879000
6	-1.978408000	-3.471063000	0.120014000
6	-0.821365000	-2.699270000	0.115729000
7	-0.834365000	-1.361772000	0.079978000
1	-4.173719000	-0.899069000	-0.019652000
1	-4.136919000	-3.373947000	0.056219000
1	-1.912634000	-4.557419000	0.153654000
1	0.161818000	-3.172212000	0.144734000
6	-1.981318000	0.779186000	0.043312000
6	-3.131987000	1.538299000	0.273141000
6	-3.051810000	2.926819000	0.243377000
6	-0.722693000	2.703544000	-0.213930000
7	-0.794096000	1.367701000	-0.186418000
1	-4.083127000	1.058936000	0.493332000
1	-3.941576000	3.530140000	0.424095000
1	-1.712550000	4.610687000	-0.046228000
1	0.263045000	3.131038000	-0.405600000
8	4.126442000	-0.194347000	-1.297167000
1	3.647600000	-0.287616000	-2.141838000
6	4.015810000	0.065487000	0.933038000
7	4.429900000	0.188626000	2.009549000
e(CH_3/Cl)			
47	-0.995085000	0.028880000	0.147003000

6	-3.189952000	0.064697000	0.258998000
6	2.064555000	3.479582000	-0.083321000
6	2.101943000	-0.769115000	-0.030188000
6	3.263470000	-1.520577000	-0.229597000
6	3.190504000	-2.909482000	-0.203674000
6	1.959898000	-3.517784000	0.017734000
6	0.848390000	-2.699348000	0.190278000
7	0.912367000	-1.363624000	0.165759000
1	4.217366000	-1.034746000	-0.422356000
1	4.088263000	-3.507686000	-0.360267000
1	1.853615000	-4.601028000	0.048683000
1	-0.140042000	-3.131683000	0.355836000
6	2.125004000	0.727982000	-0.035175000
6	3.324840000	1.442514000	0.022914000
6	3.292933000	2.832977000	-0.002612000
6	0.915256000	2.696647000	-0.119291000
7	0.939755000	1.359594000	-0.092980000
1	4.279085000	0.926945000	0.104945000
1	4.221156000	3.402703000	0.044132000
1	1.989466000	4.565545000	-0.110121000
1	-0.071637000	3.159736000	-0.172926000
6	-4.024738000	0.167095000	1.463549000
1	-3.914566000	-0.834303000	1.934141000
1	-3.559952000	0.859742000	2.181310000
1	-5.090289000	0.374848000	1.300117000
17	-4.076526000	-0.087080000	-1.165870000
f(CH_3/COOH)			
47	-0.741579000	-0.635400000	0.129053000
6	-2.911263000	-0.714903000	0.337546000
6	0.906649000	3.693492000	0.196466000
6	2.409536000	-0.272203000	-0.063730000
6	3.772984000	-0.577506000	-0.031776000
6	4.180938000	-1.902933000	-0.137786000
6	3.218515000	-2.897540000	-0.272232000
6	1.881384000	-2.515855000	-0.278626000
7	1.482423000	-1.242357000	-0.174382000
1	4.515638000	0.207591000	0.091892000
1	5.241656000	-2.153101000	-0.111580000
1	3.488749000	-3.948429000	-0.362698000
1	1.090966000	-3.262351000	-0.372805000
6	1.921404000	1.141374000	0.024057000
6	2.783386000	2.231203000	-0.123653000
6	2.267519000	3.520726000	-0.031557000
6	0.112449000	2.556382000	0.316481000
7	0.607794000	1.317725000	0.236263000
1	3.842178000	2.087214000	-0.327856000
1	2.926481000	4.381601000	-0.146010000
1	0.460925000	4.684149000	0.271687000
1	-0.965743000	2.632016000	0.475001000
6	-3.621642000	0.271521000	-0.501549000
8	-3.368514000	1.429822000	-0.276710000
6	-3.741574000	-1.335683000	1.354619000
1	-4.076669000	-2.190875000	0.702676000
1	-3.204227000	-1.780787000	2.197578000
1	-4.660257000	-0.803290000	1.642497000
8	-4.464754000	-0.210076000	-1.397571000
1	-4.852585000	0.536070000	-1.894317000
g(CH_3/CN)			
47	1.084253000	-0.047688000	-0.157282000
6	3.263506000	-0.057791000	-0.277725000
6	-1.816001000	3.530591000	-0.047095000
6	-2.024274000	-0.713847000	0.048742000
6	-3.231042000	-1.418301000	0.026951000
6	-3.211714000	-2.808620000	0.067858000
6	-1.988300000	-3.466615000	0.127858000
6	-0.831227000	-2.694878000	0.129224000
7	-0.844081000	-1.357798000	0.087523000
1	-4.182640000	-0.895603000	-0.036358000
1	-4.146294000	-3.369463000	0.050039000
1	-1.922955000	-4.552836000	0.166057000
1	0.151879000	-3.167182000	0.167233000
6	-1.987208000	0.782984000	0.026617000
6	-3.141958000	1.548775000	0.208583000
6	-3.054272000	2.936638000	0.169779000
6	-0.711612000	2.699713000	-0.203993000
7	-0.790505000	1.364857000	-0.167276000
1	-4.103228000	1.075981000	0.396299000
1	-3.947282000	3.545219000	0.312819000
1	-1.698238000	4.612338000	-0.086786000
1	0.282248000	3.120494000	-0.366015000
6	3.951855000	0.121658000	0.943076000
7	4.445853000	0.288468000	1.986464000
6	4.109272000	-0.273445000	-1.449654000
1	4.247138000	-1.383430000	-1.380345000
1	3.599133000	-0.091880000	-2.401194000
1	5.119323000	0.157858000	-1.397790000
h(NH_2/Cl)			
47	0.995294000	-0.038054000	0.197805000
6	3.219856000	-0.059532000	0.303533000
6	-2.085152000	-3.473473000	-0.100908000

6	-2.102125000	0.774934000	-0.038128000
6	-3.254464000	1.529534000	-0.275947000
6	-3.177874000	2.918231000	-0.248676000
6	-1.952958000	3.522522000	0.011918000
6	-0.850082000	2.700488000	0.220534000
7	-0.917572000	1.365059000	0.195465000
1	-4.202396000	1.045831000	-0.501260000
1	-4.067896000	3.519357000	-0.435424000
1	-1.844057000	4.605448000	0.044924000
1	0.134283000	3.129626000	0.416341000
6	-2.131231000	-0.722229000	-0.044458000
6	-3.335617000	-1.430272000	0.000682000
6	-3.310913000	-2.820759000	-0.029014000
6	-0.931407000	-2.696442000	-0.122623000
7	-0.948819000	-1.359432000	-0.091653000
1	-4.287390000	-0.909059000	0.076758000
1	-4.242448000	-3.385804000	0.007535000
1	-2.015622000	-4.559702000	-0.131640000
1	0.053622000	-3.164406000	-0.169014000
17	4.083950000	0.095584000	-1.185144000
7	4.019570000	-0.169397000	1.322973000
1	5.040261000	-0.163318000	1.237137000
1	3.625229000	-0.266014000	2.256297000
i(NH_2/COOH)			
47	0.831652000	-0.051873000	-0.303432000
6	3.028328000	-0.095801000	-0.528391000
6	-2.091671000	3.527036000	-0.030273000
6	-2.295731000	-0.715115000	0.070605000
6	-3.504814000	-1.416585000	0.080272000
6	-3.486064000	-2.806975000	0.116939000
6	-2.261682000	-3.465757000	0.140717000
6	-1.104114000	-2.694543000	0.110338000
7	-1.115666000	-1.357909000	0.073094000
1	-4.456241000	-0.890672000	0.040993000
1	-4.421171000	-3.367260000	0.122653000
1	-2.196612000	-4.552219000	0.173611000
1	-0.120194000	-3.167192000	0.117303000
6	-2.257761000	0.781539000	0.051109000
6	-3.395875000	1.546435000	0.322766000
6	-3.310719000	2.934183000	0.279653000
6	-1.002798000	2.694856000	-0.270612000
7	-1.078347000	1.360486000	-0.230309000
1	-4.338706000	1.071538000	0.585314000
1	-4.189432000	3.543525000	0.491754000
1	-1.976768000	4.608802000	-0.078145000
1	-0.023212000	3.114584000	-0.506734000
7	3.810427000	-0.224505000	-1.545573000
1	4.832910000	-0.214478000	-1.407364000
1	3.448715000	-0.336763000	-2.489805000
6	3.900982000	0.051897000	0.727789000
8	5.102301000	0.037646000	0.704123000
8	3.147049000	0.193731000	1.811091000
1	3.734899000	0.283624000	2.584044000
j(NH_2/CN)			
47	-1.088819000	-0.047373000	-0.200253000
6	-3.318093000	-0.075263000	-0.301453000
6	2.003223000	-3.466220000	0.122341000
6	1.995755000	0.782445000	0.039403000
6	3.143064000	1.543684000	0.279980000
6	3.059940000	2.931926000	0.248117000
6	1.833426000	3.529609000	-0.019882000
6	0.735557000	2.701734000	-0.231017000
7	0.809353000	1.366531000	-0.201626000
1	4.092342000	1.065589000	0.511382000
1	3.946204000	3.537817000	0.437358000
1	1.719290000	4.611883000	-0.056245000
1	-0.249745000	3.126109000	-0.432185000
6	2.032906000	-0.714543000	0.051806000
6	3.241602000	-1.415738000	0.018660000
6	3.225307000	-2.806178000	0.055434000
6	0.844661000	-2.696402000	0.131686000
7	0.854030000	-1.359297000	0.093518000
1	4.190817000	-0.889324000	-0.053057000
1	4.160622000	-3.365496000	0.028648000
1	1.940187000	-4.552657000	0.158949000
1	-0.137458000	-3.170703000	0.174254000
7	-4.107848000	-0.202584000	-1.324394000
1	-5.129502000	-0.198951000	-1.253724000
1	-3.719133000	-0.312847000	-2.258888000
6	-4.007420000	0.073928000	0.954741000
7	-4.466779000	0.201236000	2.012003000
Au carbenoids			
a(H/H)			
79	-1.367866000	-0.000015000	0.000111000
6	-3.237119000	-0.000140000	0.000128000
1	-3.857615000	-0.000067000	-0.909450000
1	-3.857557000	-0.000544000	0.909746000
6	1.522597000	-3.489110000	-0.000135000
6	1.711199000	0.744302000	-0.000136000
6	2.877999000	1.507644000	-0.000491000

6	2.780313000	2.896281000	-0.000461000
6	1.522395000	3.489168000	-0.000077000
6	0.404039000	2.661946000	0.000230000
7	0.498207000	1.327412000	0.000194000
1	3.856437000	1.032134000	-0.000827000
1	3.683005000	3.507253000	-0.000744000
1	1.399386000	4.570924000	-0.000026000
1	-0.604907000	3.075569000	0.000519000
6	1.711244000	-0.744236000	-0.000116000
6	2.878089000	-1.507509000	-0.000003000
6	2.780481000	-2.896152000	-0.000014000
6	0.404193000	-2.661950000	-0.000213000
7	0.498286000	-1.327414000	-0.000198000
1	3.856500000	-1.031946000	0.000126000
1	3.683207000	-3.507073000	0.000079000
1	1.399650000	-4.570873000	-0.000154000
1	-0.604730000	-3.075629000	-0.000292000
b(OH/Cl)			
79	0.861332000	-0.000290000	-0.133405000
6	2.796445000	0.000078000	-0.254652000
6	-2.098590000	3.492297000	0.052573000
6	-2.263944000	-0.746109000	0.063621000
6	-3.435726000	-1.500835000	0.137653000
6	-3.350051000	-2.889678000	0.131970000
6	-2.099027000	-3.491954000	0.052652000
6	-0.978351000	-2.671150000	-0.017955000
7	-1.059391000	-1.335966000	-0.012516000
1	-4.409087000	-1.019426000	0.199232000
1	-4.255866000	-3.493400000	0.189142000
1	-1.984610000	-4.574715000	0.045146000
1	0.025204000	-3.093716000	-0.081532000
6	-2.263866000	0.746490000	0.063596000
6	-3.435569000	1.501355000	0.137468000
6	-3.349704000	2.890189000	0.131758000
6	-0.978013000	2.671339000	-0.017898000
7	-1.059236000	1.336177000	-0.012435000
1	-4.409005000	1.020081000	0.198923000
1	-4.255445000	3.494034000	0.188803000
1	-1.984038000	4.575044000	0.045057000
1	0.025607000	3.093769000	-0.081377000
8	3.439556000	0.000248000	-1.367827000
1	4.414000000	0.000454000	-1.249743000
17	3.814137000	0.000305000	1.136348000
c(OH/COOH)			
79	0.720945000	-0.000062000	-0.223594000

6	2.637332000	-0.000068000	-0.421677000
6	-2.228424000	3.491124000	0.073821000
6	-2.396443000	-0.745945000	0.099213000
6	-3.563484000	-1.502184000	0.218125000
6	-3.476214000	-2.890890000	0.205404000
6	-2.228542000	-3.491071000	0.074128000
6	-1.112526000	-2.668546000	-0.038858000
7	-1.194855000	-1.333692000	-0.026040000
1	-4.534151000	-1.021861000	0.319113000
1	-4.378215000	-3.496145000	0.296703000
1	-2.113319000	-4.573657000	0.057973000
1	-0.111725000	-3.088993000	-0.145252000
6	-2.396417000	0.746008000	0.099166000
6	-3.563401000	1.502297000	0.218319000
6	-3.476084000	2.890998000	0.205465000
6	-1.112462000	2.668550000	-0.039349000
7	-1.194836000	1.333701000	-0.026398000
1	-4.534055000	1.022017000	0.319640000
1	-4.378038000	3.496294000	0.296956000
1	-2.113168000	4.573705000	0.057541000
1	-0.111671000	3.088952000	-0.146014000
8	3.275566000	-0.000239000	-1.528284000
1	4.252994000	-0.000123000	-1.326522000
6	3.655021000	0.000212000	0.741036000
8	4.831195000	-0.000404000	0.485993000
8	3.090338000	0.000766000	1.927504000
1	3.787612000	0.000760000	2.610412000
d(OH/CN)			
79	0.931398000	-0.000069000	-0.152186000
6	2.862854000	-0.000040000	-0.247151000
6	-2.004351000	3.492208000	0.073876000
6	-2.174900000	-0.745799000	0.070550000
6	-3.343856000	-1.502260000	0.161525000
6	-3.255564000	-2.891153000	0.162754000
6	-2.004493000	-3.492114000	0.073955000
6	-0.885791000	-2.670733000	-0.014933000
7	-0.969974000	-1.335153000	-0.017836000
1	-4.317155000	-1.021977000	0.232702000
1	-4.159554000	-3.496085000	0.234201000
1	-1.888158000	-4.574696000	0.074002000
1	0.117652000	-3.092512000	-0.084628000
6	-2.174872000	0.745903000	0.070520000
6	-3.343814000	1.502412000	0.161268000
6	-3.255463000	2.891302000	0.162473000
6	-0.885670000	2.670776000	-0.014819000

7	-0.969910000	1.335203000	-0.017705000
1	-4.317148000	1.022174000	0.232258000
1	-4.159441000	3.496274000	0.233740000
1	-1.887971000	4.574785000	0.073920000
1	0.117803000	3.092512000	-0.084361000
8	3.630882000	-0.000109000	-1.287163000
1	3.103748000	-0.000210000	-2.106714000
6	3.646607000	0.000132000	0.969548000
7	4.179680000	0.000276000	1.998034000
e(CH_3/Cl)			
79	-0.861959000	-0.002756000	0.111506000
6	-2.774079000	0.003728000	0.220699000
6	2.079273000	3.493297000	-0.032452000
6	2.261039000	-0.742689000	-0.049425000
6	3.432571000	-1.498382000	-0.110505000
6	3.344509000	-2.887208000	-0.114835000
6	2.091497000	-3.488112000	-0.058828000
6	0.970687000	-2.666476000	0.000689000
7	1.054268000	-1.331585000	0.005812000
1	4.407182000	-1.017384000	-0.154895000
1	4.250060000	-3.492140000	-0.162353000
1	1.975530000	-4.570720000	-0.061145000
1	-0.034765000	-3.087051000	0.044944000
6	2.258520000	0.748507000	-0.043836000
6	3.427327000	1.508732000	-0.100263000
6	3.334317000	2.897255000	-0.094130000
6	0.961334000	2.667360000	0.021856000
7	1.049779000	1.332735000	0.016786000
1	4.403585000	1.031530000	-0.149253000
1	4.237679000	3.505726000	-0.137971000
1	1.959490000	4.575475000	-0.026510000
1	-0.045532000	3.084073000	0.070312000
6	-3.583111000	-0.054708000	1.458413000
1	-3.880788000	-1.117004000	1.564501000
1	-2.992201000	0.220980000	2.338622000
1	-4.510136000	0.531399000	1.381459000
17	-3.719236000	0.038643000	-1.191717000
f(CH_3/COOH)			
79	-0.721985000	-0.033584000	0.218603000
6	-2.612309000	-0.040760000	0.420194000
6	2.116824000	3.522255000	-0.101360000
6	2.383840000	-0.708436000	-0.100904000
6	3.563628000	-1.444233000	-0.216214000
6	3.500011000	-2.834378000	-0.208358000
6	2.262276000	-3.456944000	-0.086425000

6	1.130946000	-2.655631000	0.024695000
7	1.191703000	-1.319096000	0.018115000
1	4.525901000	-0.946030000	-0.311546000
1	4.412710000	-3.423711000	-0.297506000
1	2.165730000	-4.541402000	-0.076712000
1	0.136411000	-3.092569000	0.122014000
6	2.353992000	0.781941000	-0.103859000
6	3.501635000	1.565288000	-0.228537000
6	3.378949000	2.951779000	-0.226536000
6	1.020390000	2.674636000	0.019161000
7	1.138468000	1.342326000	0.018626000
1	4.483632000	1.107954000	-0.327750000
1	4.265418000	3.578690000	-0.323395000
1	1.974989000	4.601767000	-0.096805000
1	0.008462000	3.069559000	0.118559000
6	-3.468506000	0.090133000	-0.801294000
8	-3.879376000	1.171024000	-1.122775000
6	-3.395294000	-0.055198000	1.667749000
1	-4.085746000	-0.919859000	1.627084000
1	-2.797396000	-0.077621000	2.581695000
1	-4.053982000	0.834322000	1.645882000
8	-3.680878000	-1.065009000	-1.419961000
1	-4.246792000	-0.891382000	-2.196353000
g(CH_3/CN)			
79	0.914264000	0.000393000	-0.119742000
6	2.818703000	0.000046000	-0.226835000
6	-1.988975000	3.489956000	0.056423000
6	-2.171058000	-0.745177000	0.054647000
6	-3.337663000	-1.506478000	0.124513000
6	-3.242666000	-2.895111000	0.124892000
6	-1.987706000	-3.490562000	0.056248000
6	-0.870299000	-2.665225000	-0.011882000
7	-0.961983000	-1.330478000	-0.013482000
1	-4.313751000	-1.029418000	0.179442000
1	-4.144877000	-3.504404000	0.179683000
1	-1.866932000	-4.572643000	0.055848000
1	0.136535000	-3.080856000	-0.065700000
6	-2.171307000	0.744495000	0.054699000
6	-3.338173000	1.505371000	0.124797000
6	-3.243703000	2.894038000	0.125237000
6	-0.871274000	2.665039000	-0.011904000
7	-0.962445000	1.330247000	-0.013558000
1	-4.314068000	1.027937000	0.179889000
1	-4.146133000	3.502991000	0.180217000
1	-1.868588000	4.572080000	0.056053000

1	0.135396000	3.081051000	-0.065842000
6	3.561007000	-0.000201000	0.994656000
7	4.141659000	-0.000483000	2.001750000
6	3.658109000	-0.000066000	-1.445672000
1	4.333369000	-0.874978000	-1.404477000
1	3.079893000	-0.000699000	-2.372353000
1	4.331740000	0.876175000	-1.405005000
h(NH_2/Cl)			
79	-0.880676000	-0.132024000	-0.144389000
6	-2.837646000	0.050828000	-0.235119000
6	2.176997000	-3.424921000	0.098282000
6	2.269106000	0.822002000	0.049073000
6	3.434397000	1.588032000	0.130240000
6	3.331452000	2.975847000	0.113348000
6	2.073878000	3.561820000	0.016966000
6	0.964409000	2.724339000	-0.059624000
7	1.060584000	1.393105000	-0.044846000
1	4.413756000	1.121273000	0.207914000
1	4.229071000	3.591097000	0.176539000
1	1.947516000	4.643323000	0.002253000
1	-0.044514000	3.134027000	-0.134387000
6	2.284649000	-0.671793000	0.065578000
6	3.469576000	-1.405894000	0.150082000
6	3.415181000	-2.795292000	0.166469000
6	1.041090000	-2.628828000	0.014393000
7	1.091224000	-1.289504000	-0.002502000
1	4.432477000	-0.903485000	0.203261000
1	4.333991000	-3.378121000	0.232796000
1	2.083341000	-4.509545000	0.109618000
1	0.046974000	-3.073671000	-0.040280000
17	-3.759512000	0.213540000	1.211248000
7	-3.576017000	0.073810000	-1.310854000
1	-4.591920000	0.174241000	-1.270146000
1	-3.134665000	-0.018171000	-2.222773000
i(NH_2/COOH)			
79	0.742232000	-0.000102000	-0.241989000
6	2.692263000	-0.000271000	-0.419566000
6	-2.248071000	3.491670000	0.083356000
6	-2.411218000	-0.746512000	0.095821000
6	-3.579618000	-1.500379000	0.223979000
6	-3.494901000	-2.889082000	0.217616000
6	-2.248475000	-3.491501000	0.083992000
6	-1.132616000	-2.669921000	-0.039251000
7	-1.211746000	-1.335253000	-0.034026000
1	-4.549159000	-1.018599000	0.328546000
1	-4.397320000	-3.492477000	0.316880000
1	-2.134636000	-4.574354000	0.074722000
1	-0.132407000	-3.092294000	-0.146128000
6	-2.411126000	0.746701000	0.095739000
6	-3.579338000	1.500725000	0.224684000
6	-3.494464000	2.889417000	0.218033000
6	-1.132391000	2.669940000	-0.040506000
7	-1.211670000	1.335282000	-0.034994000
1	-4.548837000	1.019073000	0.330215000
1	-4.396733000	3.492933000	0.317922000
1	-2.134115000	4.574508000	0.073802000
1	-0.132208000	3.092180000	-0.148153000
7	3.405249000	-0.000824000	-1.502888000
1	4.429975000	-0.000845000	-1.419587000
1	2.978372000	-0.001227000	-2.424864000
6	3.633495000	0.000244000	0.796342000
8	4.828578000	0.000107000	0.676079000
8	2.963603000	0.000825000	1.937359000
1	3.609242000	0.001119000	2.668174000
j(NH_2/CN)			
79	0.941196000	0.000014000	-0.149790000
6	2.903435000	-0.000010000	-0.239030000
6	-2.038002000	3.492381000	0.069172000
6	-2.201011000	-0.746519000	0.066027000
6	-3.372812000	-1.500287000	0.154080000
6	-3.288142000	-2.889099000	0.155289000
6	-2.037986000	-3.492395000	0.069245000
6	-0.918016000	-2.672058000	-0.016666000
7	-0.997753000	-1.336871000	-0.019403000
1	-4.345377000	-1.018274000	0.222946000
1	-4.193688000	-3.491965000	0.224526000
1	-1.923915000	-4.575238000	0.069326000
1	0.084973000	-3.095674000	-0.084088000
6	-2.201012000	0.746504000	0.066031000
6	-3.372793000	1.500267000	0.154380000
6	-3.288132000	2.889080000	0.155548000
6	-0.918046000	2.672051000	-0.016981000
7	-0.997776000	1.336862000	-0.019673000
1	-4.345334000	1.018250000	0.223551000
1	-4.193662000	3.491942000	0.225019000
1	-1.923937000	4.575226000	0.069198000
1	0.084924000	3.095671000	-0.084648000
7	3.658432000	-0.000090000	-1.305210000
1	4.678430000	-0.000112000	-1.252435000
1	3.236756000	-0.000138000	-2.230641000

6	3.645823000	0.000005000	1.001526000
7	4.195174000	0.000056000	2.021272000
Methane (me)			
6	0.000000000	0.000000000	0.000000000
1	0.633483000	0.633483000	0.633483000
1	-0.633483000	-0.633483000	0.633483000
1	0.633483000	-0.633483000	-0.633483000
1	-0.633483000	0.633483000	-0.633483000
Transition Step (TS)			
Ni carbenoid reactions			
a(H/H)			
6	0.032554000	-3.754951000	-0.335069000
28	0.000020000	0.841480000	0.187275000
6	4.305555000	1.146229000	0.282612000
6	2.952676000	1.475322000	0.291287000
7	1.978481000	0.564831000	0.163128000
6	2.335897000	-0.743614000	0.024288000
6	3.663843000	-1.146387000	0.005682000
6	4.667894000	-0.186818000	0.135928000
1	2.648280000	2.512888000	0.412649000
1	3.917356000	-2.199752000	-0.104974000
1	5.717252000	-0.482593000	0.125924000
1	5.051627000	1.931853000	0.393558000
6	1.193627000	-1.678623000	-0.090491000
7	0.013536000	-1.050567000	-0.016401000
6	-1.157711000	-1.695457000	-0.091476000
6	-1.185553000	-3.078189000	-0.254639000
6	1.240618000	-3.061293000	-0.253455000
1	-2.125389000	-3.624054000	-0.318236000
1	0.040155000	-4.837832000	-0.462617000
1	2.188145000	-3.593762000	-0.316430000
6	-2.313428000	-0.777034000	0.025313000
7	-1.974522000	0.534710000	0.174045000
6	-2.961151000	1.430516000	0.310739000
6	-4.309290000	1.084833000	0.296776000
6	-4.653111000	-0.252190000	0.137418000
6	-3.636417000	-1.197104000	0.001821000
1	-2.672400000	2.471417000	0.449884000
1	-5.066262000	1.858916000	0.414912000
1	-5.698441000	-0.561807000	0.123275000
1	-3.875831000	-2.252917000	-0.116605000
6	-0.023252000	2.816808000	0.391210000
1	-0.735622000	3.393274000	-0.520538000
6	-0.083275000	3.589273000	-1.512644000
1	0.877244000	3.417263000	0.560743000
1	0.106659000	4.666122000	-1.464933000
1	-0.913820000	3.318524000	-2.180047000
1	0.803366000	2.992225000	-1.722809000
1	-0.802200000	3.258057000	1.045350000
b(OH/Cl)			
6	0.750874000	4.063214000	0.584092000
28	0.007977000	-0.427260000	-0.175634000
6	-4.245131000	0.080231000	-0.753901000
6	-2.982653000	-0.491870000	-0.639083000
7	-1.884121000	0.212789000	-0.337512000
6	-2.014462000	1.554878000	-0.151288000
6	-3.243866000	2.194946000	-0.231875000
6	-4.380111000	1.446356000	-0.535300000
1	-2.857803000	-1.563024000	-0.785436000
1	-3.315653000	3.269763000	-0.071923000
1	-5.355010000	1.929245000	-0.607288000
1	-5.100491000	-0.544946000	-1.005658000
6	-0.737763000	2.255099000	0.101625000
7	0.319308000	1.436305000	0.004208000
6	1.579501000	1.856130000	0.176612000
6	1.835024000	3.192937000	0.474682000
6	-0.553285000	3.603214000	0.393367000
1	2.851383000	3.554071000	0.622405000
1	0.923471000	5.113726000	0.819851000
1	-1.398138000	4.284599000	0.478193000
6	2.563367000	0.773322000	-0.027395000
7	2.007470000	-0.449908000	-0.261858000
6	2.825989000	-1.463056000	-0.577714000
6	4.211323000	-1.323645000	-0.636431000
6	4.777561000	-0.090042000	-0.347668000
6	3.934220000	0.979923000	-0.045741000
1	2.361458000	-2.423448000	-0.778202000
1	4.823893000	-2.183792000	-0.902622000
1	5.858588000	0.049695000	-0.371214000
1	4.344131000	1.968683000	0.155032000
6	-0.361491000	-2.479309000	0.256947000
1	-0.710082000	-2.491307000	-0.867831000
6	-1.103050000	-3.813356000	-0.862825000
17	-1.555048000	-2.409165000	1.550407000
1	-2.093392000	-3.593449000	-1.281793000
1	-1.172448000	-4.536400000	-0.045343000
1	-0.356114000	-4.095005000	-1.615763000
8	0.812707000	-3.049777000	0.597095000
1	0.672126000	-3.849256000	1.139175000
c(OH/COOH)			

6	1.040810000	4.016512000	0.866617000
28	0.017923000	-0.332722000	-0.300816000
6	-4.155529000	0.530970000	-0.986789000
6	-2.943937000	-0.140872000	-0.862986000
7	-1.815434000	0.452296000	-0.454368000
6	-1.860178000	1.782201000	-0.166082000
6	-3.036145000	2.515928000	-0.244703000
6	-4.205909000	1.880715000	-0.658960000
1	-2.887594000	-1.199911000	-1.102064000
1	-3.037917000	3.578122000	-0.004327000
1	-5.139044000	2.439491000	-0.736020000
1	-5.037587000	-0.005176000	-1.333714000
6	-0.548809000	2.367818000	0.180527000
7	0.453727000	1.491230000	0.024304000
6	1.732914000	1.806304000	0.264901000
6	2.067192000	3.088493000	0.697307000
6	-0.285276000	3.666265000	0.604671000
1	3.100652000	3.362740000	0.902200000
1	1.275488000	5.025569000	1.206702000
1	-1.086048000	4.391760000	0.737893000
6	2.649087000	0.685913000	-0.026865000
7	2.020284000	-0.472944000	-0.378167000
6	2.777678000	-1.492913000	-0.805591000
6	4.170115000	-1.429553000	-0.853106000
6	4.809105000	-0.271692000	-0.435124000
6	4.029409000	0.810913000	-0.024760000
1	2.255698000	-2.395434000	-1.106678000
1	4.729765000	-2.291618000	-1.213195000
1	5.896670000	-0.195699000	-0.444979000
1	4.496331000	1.749130000	0.271339000
6	-0.451866000	-2.392282000	0.029610000
1	-1.050832000	-2.333097000	-0.995341000
6	-1.739934000	-3.517755000	-0.914615000
1	-2.740158000	-3.279786000	-0.543171000
1	-1.302172000	-4.424021000	-0.490567000
1	-1.659421000	-3.520410000	-2.012926000
8	0.662542000	-3.155181000	-0.001603000
1	0.652712000	-3.735941000	0.789610000
6	-1.153622000	-2.484421000	1.371559000
8	-0.644581000	-3.117986000	2.253056000
8	-2.302297000	-1.817126000	1.448578000
1	-2.674373000	-1.956485000	2.341788000
d(OH/CN)			
6	0.544691000	4.061222000	0.559597000
28	-0.002697000	-0.460999000	-0.118451000
6	-4.252454000	-0.164223000	-0.770413000
6	-2.961677000	-0.674144000	-0.657327000
7	-1.907655000	0.080261000	-0.320156000
6	-2.107333000	1.415519000	-0.125333000
6	-3.366289000	1.990545000	-0.203288000
6	-4.461767000	1.185438000	-0.520533000
1	-2.779129000	-1.731296000	-0.838128000
1	-3.495203000	3.058563000	-0.033151000
1	-5.461134000	1.616577000	-0.585282000
1	-5.070384000	-0.829053000	-1.043956000
6	-0.865114000	2.180728000	0.112711000
7	0.229303000	1.415399000	0.003278000
6	1.471550000	1.893537000	0.161455000
6	1.669702000	3.241822000	0.443109000
6	-0.738399000	3.539432000	0.393916000
1	2.669132000	3.652226000	0.578420000
1	0.670837000	5.120606000	0.785190000
1	-1.613105000	4.180726000	0.489523000
6	2.500606000	0.847257000	-0.026443000
7	1.997309000	-0.411753000	-0.179390000
6	2.853380000	-1.406342000	-0.470274000
6	4.226981000	-1.213188000	-0.579984000
6	4.741615000	0.062516000	-0.378868000
6	3.861159000	1.109429000	-0.105474000
1	2.427611000	-2.400216000	-0.603743000
1	4.871655000	-2.058657000	-0.816477000
1	5.813856000	0.248737000	-0.447846000
1	4.232776000	2.124873000	0.025566000
6	-0.208391000	-2.487915000	0.398033000
1	-0.212130000	-2.719561000	-0.792848000
6	-0.423536000	-4.020074000	-0.812294000
1	-1.448118000	-4.046290000	-1.197085000
1	-0.240281000	-4.686245000	0.034177000
1	0.360382000	-4.102951000	-1.579628000
8	0.868274000	-2.984633000	1.086370000
1	1.412264000	-2.255922000	1.423356000
6	-1.472789000	-2.674751000	1.089175000
7	-2.473430000	-2.756469000	1.664980000
e(CH_3/Cl)			
6	0.722264000	4.038540000	0.685571000
28	0.017230000	-0.441945000	-0.191690000
6	-4.230206000	0.070748000	-0.847690000
6	-2.969216000	-0.500436000	-0.710266000
7	-1.879576000	0.202860000	-0.379629000
6	-2.016556000	1.541751000	-0.181575000

6	-3.244666000	2.181620000	-0.282181000
6	-4.372442000	1.434386000	-0.619150000
1	-2.836593000	-1.569824000	-0.860750000
1	-3.321756000	3.254691000	-0.113063000
1	-5.346265000	1.916483000	-0.708372000
1	-5.078978000	-0.553094000	-1.123728000
6	-0.746765000	2.240695000	0.109794000
7	0.315319000	1.431424000	0.001019000
6	1.569384000	1.852151000	0.215849000
6	1.813595000	3.176810000	0.568258000
6	-0.574461000	3.579712000	0.451949000
1	2.824833000	3.536669000	0.750818000
1	0.884560000	5.080581000	0.962552000
1	-1.424722000	4.253257000	0.545428000
6	2.563690000	0.784372000	-0.023883000
7	2.015234000	-0.427275000	-0.320022000
6	2.833664000	-1.406383000	-0.726906000
6	4.216687000	-1.261659000	-0.791009000
6	4.779299000	-0.047754000	-0.416099000
6	3.934893000	0.997427000	-0.041628000
1	2.364618000	-2.339218000	-1.027951000
1	4.830508000	-2.094039000	-1.132307000
1	5.859602000	0.098024000	-0.439092000
1	4.342780000	1.975300000	0.210440000
6	-0.340577000	-2.451158000	0.338783000
1	-0.798572000	-2.620080000	-0.774025000
6	-1.179000000	-3.866065000	-0.790031000
17	-1.575919000	-2.269028000	1.588704000
1	-2.150428000	-3.612152000	-1.234949000
1	-1.290810000	-4.502807000	0.088397000
1	-0.445228000	-4.235606000	-1.515320000
6	0.810768000	-3.295412000	0.839736000
1	1.462856000	-2.663387000	1.459568000
1	0.450660000	-4.124415000	1.465892000
1	1.402148000	-3.717589000	0.019116000
f(CH_3/COOH)			
6	1.647234000	3.966055000	0.549260000
28	0.003563000	-0.290401000	-0.067754000
6	-4.004859000	1.037488000	-0.900452000
6	-2.886654000	0.226917000	-0.723869000
7	-1.699557000	0.705441000	-0.332454000
6	-1.568776000	2.050464000	-0.160793000
6	-2.643732000	2.916859000	-0.294380000
6	-3.888134000	2.400204000	-0.656969000
1	-2.946407000	-0.842575000	-0.924747000

1	-2.511162000	3.987108000	-0.141706000
1	-4.746570000	3.062602000	-0.770986000
1	-4.945673000	0.595757000	-1.225274000
6	-0.178439000	2.490314000	0.090998000
7	0.694930000	1.480358000	-0.018820000
6	2.014714000	1.636985000	0.153919000
6	2.536919000	2.895376000	0.439297000
6	0.275477000	3.775925000	0.376719000
1	3.605631000	3.046231000	0.583693000
1	2.028529000	4.961767000	0.777679000
1	-0.415509000	4.612040000	0.471810000
6	2.750752000	0.364925000	-0.023306000
7	1.944630000	-0.722526000	-0.174370000
6	2.508872000	-1.898414000	-0.480519000
6	3.886073000	-2.066174000	-0.594573000
6	4.712188000	-0.968172000	-0.382298000
6	4.133154000	0.270071000	-0.103175000
1	1.830463000	-2.734569000	-0.648809000
1	4.291981000	-3.044613000	-0.847578000
1	5.795939000	-1.064695000	-0.453504000
1	4.753927000	1.155529000	0.027523000
6	-0.702919000	-2.074558000	0.588856000
1	-0.546660000	-2.829188000	-0.985621000
6	-0.612822000	-3.843917000	-1.479211000
1	-1.450496000	-3.783494000	-2.182965000
1	-0.793810000	-4.620133000	-0.729286000
1	0.335632000	-4.003794000	-2.006965000
6	-2.162374000	-2.217432000	0.883309000
8	-2.540309000	-1.497469000	1.769635000
8	-2.871273000	-3.087048000	0.189937000
1	-3.781611000	-3.112853000	0.548555000
6	0.079639000	-2.902699000	1.529132000
1	-0.114942000	-2.365068000	2.486053000
1	-0.315822000	-3.919477000	1.675730000
1	1.161560000	-2.897441000	1.374766000
g(CH_3/CN)			
6	0.450706000	4.080573000	0.618366000
28	-0.006457000	-0.448456000	-0.109498000
6	-4.256251000	-0.200234000	-0.822153000
6	-2.958479000	-0.693959000	-0.701428000
7	-1.926140000	0.064269000	-0.311459000
6	-2.151196000	1.392139000	-0.090882000
6	-3.417678000	1.948863000	-0.172359000
6	-4.494959000	1.134953000	-0.529146000
1	-2.752066000	-1.737710000	-0.923750000

1	-3.565584000	3.010858000	0.018764000
1	-5.500300000	1.551287000	-0.597516000
1	-5.055562000	-0.869079000	-1.137806000
6	-0.922875000	2.177393000	0.153299000
7	0.185617000	1.440290000	0.004147000
6	1.418846000	1.944531000	0.154879000
6	1.591897000	3.290215000	0.462676000
6	-0.822327000	3.532098000	0.465391000
1	2.584307000	3.719909000	0.589554000
1	0.556564000	5.137262000	0.865807000
1	-1.710003000	4.149495000	0.593339000
6	2.467034000	0.923895000	-0.065375000
7	1.983791000	-0.337525000	-0.234889000
6	2.848003000	-1.310220000	-0.554198000
6	4.217163000	-1.098706000	-0.677086000
6	4.714915000	0.181355000	-0.456397000
6	3.823243000	1.209617000	-0.153912000
1	2.428297000	-2.302067000	-0.719667000
1	4.872284000	-1.927593000	-0.941361000
1	5.783329000	0.384534000	-0.534902000
1	4.181056000	2.228049000	-0.008526000
6	-0.161162000	-2.426573000	0.523650000
1	0.168522000	-2.925135000	-0.626583000
6	0.165385000	-4.147758000	-0.792435000
1	-0.695838000	-4.235848000	-1.464675000
1	0.090557000	-4.774855000	0.096201000
1	1.140676000	-4.214941000	-1.292930000
6	0.801050000	-2.753375000	1.646617000
1	0.567023000	-2.028064000	2.446126000
1	0.651200000	-3.756421000	2.069927000
1	1.851173000	-2.609649000	1.372970000
6	-1.498787000	-2.867246000	0.839779000
7	-2.572125000	-3.188268000	1.137239000
h(NH_2/Cl)			
6	0.759041000	4.048563000	0.664813000
28	0.006523000	-0.431364000	-0.133211000
6	-4.213707000	0.114096000	-0.883070000
6	-2.959103000	-0.468516000	-0.730766000
7	-1.874584000	0.222517000	-0.357491000
6	-2.006125000	1.561394000	-0.148538000
6	-3.228866000	2.208703000	-0.257598000
6	-4.354827000	1.472214000	-0.625194000
1	-2.828830000	-1.534805000	-0.904929000
1	-3.302934000	3.279849000	-0.075333000
1	-5.324944000	1.960530000	-0.721095000

1	-5.058574000	-0.498846000	-1.193461000
6	-0.731614000	2.253439000	0.139259000
7	0.324490000	1.436241000	0.027328000
6	1.585454000	1.851030000	0.209328000
6	1.843631000	3.179795000	0.536130000
6	-0.545026000	3.595251000	0.461521000
1	2.860194000	3.536780000	0.693066000
1	0.932638000	5.093146000	0.924974000
1	-1.389071000	4.275946000	0.559746000
6	2.567094000	0.770643000	-0.026413000
7	2.008549000	-0.455424000	-0.241693000
6	2.815232000	-1.462000000	-0.614016000
6	4.194939000	-1.314902000	-0.737940000
6	4.767630000	-0.080494000	-0.460466000
6	3.935417000	0.982626000	-0.107789000
1	2.341112000	-2.424330000	-0.794741000
1	4.798594000	-2.167686000	-1.045372000
1	5.845627000	0.065420000	-0.534957000
1	4.350005000	1.972571000	0.077647000
6	-0.344027000	-2.454742000	0.359439000
1	-0.598559000	-2.531545000	-0.809594000
6	-0.874483000	-3.828098000	-0.875685000
1	-1.831111000	-3.652438000	-1.385238000
1	-0.986849000	-4.552789000	-0.066519000
1	-0.036155000	-4.060143000	-1.543318000
17	-1.768237000	-2.459223000	1.435805000
7	0.806444000	-3.072943000	0.846219000
1	1.368141000	-2.440734000	1.416115000
1	0.619839000	-3.922679000	1.385599000
i(NH_2/COOH)			
6	1.359196000	3.939221000	0.736510000
28	0.037018000	-0.367324000	-0.213030000
6	-4.039044000	0.742135000	-1.068200000
6	-2.871786000	-0.002167000	-0.918508000
7	-1.726901000	0.527994000	-0.469048000
6	-1.694028000	1.861384000	-0.194730000
6	-2.823044000	2.661243000	-0.298668000
6	-4.019615000	2.090763000	-0.733295000
1	-2.862627000	-1.062950000	-1.164659000
1	-2.768787000	3.723585000	-0.064743000
1	-4.917795000	2.701696000	-0.827291000
1	-4.940910000	0.261069000	-1.444151000
6	-0.345744000	2.373425000	0.135733000
7	0.598702000	1.433495000	-0.004737000
6	1.900296000	1.671730000	0.202979000

6	2.324705000	2.943408000	0.579687000
6	0.008809000	3.666208000	0.512515000
1	3.376992000	3.159620000	0.757329000
1	1.663384000	4.941955000	1.038078000
1	-0.741353000	4.445576000	0.636632000
6	2.731983000	0.473556000	-0.046408000
7	2.015119000	-0.663873000	-0.274568000
6	2.678674000	-1.775161000	-0.629546000
6	4.066546000	-1.819892000	-0.736277000
6	4.798427000	-0.672081000	-0.458693000
6	4.117301000	0.496185000	-0.113768000
1	2.077912000	-2.665566000	-0.807407000
1	4.552629000	-2.749222000	-1.029833000
1	5.886907000	-0.675219000	-0.522923000
1	4.662465000	1.419296000	0.078491000
6	-0.594345000	-2.317847000	0.299959000
1	-0.702234000	-2.505450000	-0.891494000
6	-1.118092000	-3.746254000	-0.901838000
1	-1.945821000	-3.544048000	-1.592332000
1	-1.446033000	-4.369333000	-0.069217000
1	-0.206236000	-4.127140000	-1.374614000
7	0.458903000	-2.960871000	0.969172000
1	1.144488000	-2.325839000	1.370072000
1	0.152163000	-3.616776000	1.689441000
6	-1.834274000	-2.053239000	1.125167000
8	-1.737481000	-1.383143000	2.116774000
8	-2.967312000	-2.603889000	0.679640000
1	-3.679851000	-2.382818000	1.310710000
j(NH_2/CN)			
6	0.587492000	4.048316000	0.585545000
28	0.001583000	-0.466255000	-0.110508000
6	-4.240040000	-0.114470000	-0.813819000
6	-2.955774000	-0.639100000	-0.694177000
7	-1.899167000	0.098079000	-0.328586000
6	-2.087968000	1.431820000	-0.115854000
6	-3.340420000	2.020633000	-0.196510000
6	-4.439856000	1.231903000	-0.539726000
1	-2.781205000	-1.694395000	-0.892857000
1	-3.460560000	3.086966000	-0.010362000
1	-5.434341000	1.673694000	-0.607478000
1	-5.060480000	-0.765833000	-1.111084000
6	-0.839345000	2.181963000	0.133942000
7	0.248400000	1.407636000	0.016628000
6	1.495350000	1.875929000	0.167607000
6	1.704966000	3.221423000	0.455581000
6	-0.700564000	3.537420000	0.423254000
1	2.708743000	3.622989000	0.584889000
1	0.723056000	5.105280000	0.816882000
1	-1.569889000	4.184897000	0.525777000
6	2.514629000	0.824243000	-0.036135000
7	1.999295000	-0.429531000	-0.190440000
6	2.842661000	-1.430145000	-0.491973000
6	4.217005000	-1.245095000	-0.616460000
6	4.744399000	0.024696000	-0.416190000
6	3.875512000	1.077485000	-0.127034000
1	2.406027000	-2.420157000	-0.610529000
1	4.852499000	-2.094471000	-0.863247000
1	5.817164000	0.202787000	-0.496729000
1	4.256125000	2.089316000	0.005726000
6	-0.251437000	-2.513187000	0.426924000
1	-0.241329000	-2.657391000	-0.766467000
6	-0.471570000	-3.970723000	-0.845109000
1	-1.390958000	-3.888336000	-1.437697000
1	-0.558212000	-4.704090000	-0.041510000
1	0.444155000	-4.113293000	-1.433935000
7	0.863888000	-2.965129000	1.147433000
1	1.396310000	-2.222388000	1.595114000
1	0.652902000	-3.699450000	1.825271000
6	-1.550690000	-2.634865000	1.058200000
7	-2.559619000	-2.713977000	1.621261000
k(OH/COCl)			
28	-0.069757000	-0.267384000	-0.411219000
6	0.581075000	-2.278013000	-0.260617000
6	-4.166180000	-1.497066000	-0.853421000
6	-2.774911000	-1.504050000	-0.840279000
7	-2.046252000	-0.454395000	-0.435053000
6	-2.695369000	0.688126000	-0.074754000
6	-4.081229000	0.764366000	-0.050311000
6	-4.829471000	-0.349048000	-0.433351000
1	-2.225569000	-2.376790000	-1.197182000
1	-4.576274000	1.688993000	0.243920000
1	-5.919116000	-0.308255000	-0.424418000
1	-4.709289000	-2.376558000	-1.196537000
6	-1.793785000	1.828508000	0.208243000
7	-0.512159000	1.533890000	-0.052525000
6	0.490559000	2.404722000	0.123243000
6	0.216443000	3.693589000	0.577102000
6	-2.136183000	3.097465000	0.664724000
1	1.012644000	4.420909000	0.727858000

1	-1.350997000	5.030528000	1.204045000
1	-3.169410000	3.362277000	0.883914000
6	1.803838000	1.823766000	-0.233096000
7	1.752540000	0.501680000	-0.566106000
6	2.866894000	-0.091295000	-1.013918000
6	4.085942000	0.577915000	-1.105819000
6	4.149583000	1.910740000	-0.722633000
6	2.984416000	2.547452000	-0.288736000
1	2.779564000	-1.134403000	-1.313182000
1	4.962305000	0.046847000	-1.474369000
1	5.089116000	2.461569000	-0.774597000
1	2.998603000	3.601539000	-0.014737000
6	-1.110940000	4.029393000	0.844665000
1	-0.554607000	-2.472922000	0.035903000
6	-0.481300000	-3.773011000	0.439247000
1	-1.160603000	-3.571801000	1.278706000
1	0.397464000	-4.343920000	0.752319000
1	-0.952220000	-4.194117000	-0.455716000
8	1.034688000	-2.782403000	-1.424193000
1	1.722500000	-3.457983000	-1.257562000
6	1.566027000	-2.251012000	0.874094000
8	2.689854000	-2.607541000	0.753493000
17	0.905965000	-1.563126000	2.359464000

l(CH_3/COCl)

28	-0.057036000	-0.166512000	-0.306055000
6	1.239858000	-1.699856000	-0.350753000
6	-3.335788000	-2.950333000	-0.650969000
6	-2.054435000	-2.407235000	-0.635758000
7	-1.807385000	-1.128599000	-0.321520000
6	-2.865513000	-0.320406000	-0.031296000
6	-4.169947000	-0.794710000	-0.013177000
6	-4.410885000	-2.133102000	-0.322419000
1	-1.194519000	-3.026683000	-0.885151000
1	-4.994923000	-0.125133000	0.226096000
1	-5.428448000	-2.524829000	-0.317337000
1	-3.475521000	-3.996760000	-0.918659000
6	-2.500025000	1.092531000	0.212609000
7	-1.193804000	1.317253000	0.018935000
6	-0.635084000	2.525347000	0.170732000
6	-1.418876000	3.615738000	0.539986000
6	-3.342129000	2.137544000	0.583141000
1	-0.986346000	4.606306000	0.670419000
1	-3.418860000	4.242021000	1.038897000

1	-4.406396000	1.977472000	0.747815000
6	0.817262000	2.511792000	-0.112483000
7	1.314892000	1.280648000	-0.414326000
6	2.608136000	1.186132000	-0.751788000
6	3.467252000	2.279914000	-0.781626000
6	2.964711000	3.530772000	-0.443171000
6	1.615798000	3.647361000	-0.109378000
1	2.986656000	0.197697000	-1.014701000
1	4.509581000	2.139571000	-1.064164000
1	3.609185000	4.410225000	-0.446573000
1	1.190555000	4.617856000	0.142387000
6	-2.783005000	3.406056000	0.746050000
1	0.213161000	-2.621331000	0.761724000
6	0.391722000	-3.492031000	1.461699000
1	0.301436000	-3.071976000	2.471075000
1	1.385386000	-3.928674000	1.308948000
1	-0.394726000	-4.225872000	1.246427000
6	1.515034000	-2.644451000	-1.421795000
1	2.127080000	-3.520939000	-1.166659000
1	0.650413000	-2.880790000	-2.052911000
1	2.172316000	-1.996203000	-2.059306000
6	2.286522000	-1.501225000	0.755667000
8	2.025006000	-0.880279000	1.733833000
17	3.883021000	-2.164208000	0.468076000

m(NH_2/COCl)

28	0.096589000	-0.322005000	-0.346557000
6	-0.528935000	-2.317194000	0.110620000
6	-3.948625000	0.831390000	-1.313412000
6	-2.786222000	0.079274000	-1.160176000
7	-1.660364000	0.581212000	-0.636523000
6	-1.638753000	1.902466000	-0.297682000
6	-2.762968000	2.706595000	-0.402799000
6	-3.944451000	2.158734000	-0.906337000
1	-2.772223000	-0.966783000	-1.460678000
1	-2.717793000	3.756243000	-0.115607000
1	-4.840720000	2.773002000	-0.996672000
1	-4.835470000	0.366510000	-1.741530000
6	-0.303444000	2.398872000	0.097637000
7	0.647224000	1.466161000	-0.051546000
6	1.942121000	1.695695000	0.207364000
6	2.352875000	2.951271000	0.645869000
6	0.038862000	3.675635000	0.537202000
1	3.399044000	3.160632000	0.863589000

1	1.674625000	4.930715000	1.160794000
1	-0.716034000	4.449190000	0.668904000
6	2.781563000	0.507186000	-0.059136000
7	2.071462000	-0.620343000	-0.349661000
6	2.744499000	-1.719663000	-0.724630000
6	4.134689000	-1.763500000	-0.789853000
6	4.858579000	-0.627536000	-0.448300000
6	4.168378000	0.529144000	-0.082958000
1	2.149383000	-2.601648000	-0.956172000
1	4.628933000	-2.682376000	-1.102222000
1	5.948569000	-0.630644000	-0.479115000
1	4.708260000	1.443856000	0.158397000
6	1.381083000	3.940213000	0.811830000
1	-0.593763000	-2.442090000	-1.087801000
6	-1.026919000	-3.688821000	-1.155916000
1	-1.783691000	-3.430706000	-1.907288000
1	-1.456908000	-4.315359000	-0.372511000
1	-0.091946000	-4.090527000	-1.559612000
7	0.494909000	-2.973356000	0.798986000
1	1.179551000	-2.362146000	1.235804000
1	0.176381000	-3.675563000	1.467976000
6	-1.859268000	-2.110065000	0.780568000
8	-2.932807000	-2.399712000	0.369101000
17	-1.634662000	-1.267198000	2.326258000
Pd carbenoid reactions			
a(H/H)			
46	-0.000327000	-0.856032000	-0.166849000
6	-0.015903000	-2.875415000	-0.421430000
6	0.019051000	3.852382000	0.330354000
6	4.434426000	-0.934159000	-0.244591000
6	3.099077000	-1.327177000	-0.261034000
7	2.086215000	-0.461741000	-0.141177000
6	2.364704000	0.867799000	-0.003764000
6	3.676293000	1.326372000	0.021032000
6	4.726232000	0.416180000	-0.099372000
1	2.835652000	-2.377944000	-0.381904000
1	3.884625000	2.389577000	0.129617000
1	5.759065000	0.764954000	-0.082908000
1	5.219763000	-1.681704000	-0.347932000
6	1.193455000	1.777921000	0.103291000
7	0.007732000	1.154928000	0.035241000
6	-1.172616000	1.787943000	0.101812000
6	-1.197263000	3.173616000	0.253811000
6	1.229722000	3.163255000	0.255279000

1	-2.135420000	3.722556000	0.312343000
1	0.023584000	4.936155000	0.449438000
1	2.172409000	3.704255000	0.314982000
6	-2.351302000	0.887883000	-0.007329000
7	-2.083948000	-0.444153000	-0.143646000
6	-3.103906000	-1.300867000	-0.266184000
6	-4.435882000	-0.895790000	-0.253522000
6	-4.716392000	0.456897000	-0.109097000
6	-3.658792000	1.357925000	0.014091000
1	-2.848522000	-2.353622000	-0.385777000
1	-5.227326000	-1.636521000	-0.359265000
1	-5.746163000	0.814736000	-0.095426000
1	-3.858061000	2.422915000	0.122214000
1	0.571978000	-3.563015000	0.522262000
6	-0.090792000	-3.668311000	1.514337000
1	0.804113000	-3.313837000	-1.023556000
1	0.687310000	-3.325648000	2.208344000
1	-0.247095000	-4.753697000	1.514564000
1	-1.012469000	-3.098815000	1.624934000
1	-0.939059000	-3.414812000	-0.665924000
b(OH/Cl)			
46	-0.021696000	-0.448811000	-0.107012000
6	-0.495625000	-2.592736000	0.026028000
6	0.942472000	4.169838000	0.254278000
6	-4.383020000	0.445084000	-0.300520000
6	-3.168424000	-0.233352000	-0.266384000
7	-1.988558000	0.388530000	-0.152964000
6	-1.972732000	1.749406000	-0.066767000
6	-3.149960000	2.488123000	-0.086982000
6	-4.373239000	1.830632000	-0.205801000
1	-3.148163000	-1.320517000	-0.313631000
1	-3.118777000	3.573752000	-0.012840000
1	-5.303387000	2.399062000	-0.223946000
1	-5.311693000	-0.116013000	-0.395069000
6	-0.636264000	2.380231000	0.042009000
7	0.392262000	1.515474000	0.005817000
6	1.677655000	1.895212000	0.081844000
6	1.986653000	3.249112000	0.210374000
6	-0.382587000	3.743979000	0.167861000
1	3.019041000	3.586940000	0.276953000
1	1.162751000	5.232711000	0.356271000
1	-1.194241000	4.468171000	0.201746000
6	2.648555000	0.780542000	0.004254000
7	2.116630000	-0.472543000	-0.106547000
6	2.947939000	-1.515034000	-0.212849000

6	4.335365000	-1.375205000	-0.205395000
6	4.881727000	-0.107116000	-0.079227000
6	4.021190000	0.986064000	0.024881000
1	2.483249000	-2.492681000	-0.300094000
1	4.961924000	-2.261346000	-0.296457000
1	5.961844000	0.040071000	-0.065056000
1	4.425233000	1.992668000	0.117344000
1	-0.872292000	-2.271747000	-1.047441000
6	-1.390215000	-3.513473000	-1.348140000
17	-1.625893000	-2.743036000	1.363642000
1	-2.352272000	-3.084748000	-1.657749000
1	-1.534007000	-4.412671000	-0.742082000
1	-0.684315000	-3.664812000	-2.174220000
8	0.638321000	-3.311381000	0.135267000
1	0.457455000	-4.208953000	0.473855000
c(OH/COOH)			
46	0.000183000	-0.307716000	-0.135415000
6	0.269433000	-2.432836000	-0.569712000
6	-0.263418000	4.399132000	-0.084318000
6	-4.394565000	-0.653080000	0.248597000
6	-3.042828000	-0.967679000	0.142652000
7	-2.092548000	-0.035168000	0.008876000
6	-2.447358000	1.281859000	0.025968000
6	-3.779991000	1.663560000	0.123405000
6	-4.768616000	0.685043000	0.224248000
1	-2.701665000	-2.001143000	0.197269000
1	-4.050163000	2.718373000	0.135539000
1	-5.817781000	0.971730000	0.300842000
1	-5.129466000	-1.449818000	0.354921000
6	-1.327300000	2.254678000	-0.014536000
7	-0.108696000	1.689109000	0.018728000
6	1.037257000	2.387461000	-0.009302000
6	0.985107000	3.780522000	-0.057751000
6	-1.435753000	3.642147000	-0.063968000
1	1.895219000	4.377222000	-0.083442000
1	-0.325311000	5.486818000	-0.126893000
1	-2.407148000	4.132626000	-0.093770000
6	2.259237000	1.548468000	0.029527000
7	2.055768000	0.199342000	-0.010020000
6	3.105447000	-0.619952000	0.105201000
6	4.411356000	-0.147945000	0.230881000
6	4.631030000	1.222018000	0.232736000
6	3.535699000	2.082112000	0.138384000
1	2.892147000	-1.686591000	0.106450000
1	5.231023000	-0.858721000	0.325769000

1	5.639645000	1.626225000	0.321111000
1	3.681512000	3.160908000	0.164018000
1	-0.779099000	-2.282880000	-1.120664000
6	-1.048281000	-3.582488000	-1.401274000
1	-1.784710000	-3.880560000	-0.650830000
1	-0.295285000	-4.344563000	-1.614570000
1	-1.480347000	-3.193986000	-2.336260000
8	1.333372000	-2.542187000	-1.393082000
1	1.819575000	-3.363564000	-1.176862000
6	0.394486000	-3.168632000	0.735015000
8	1.444015000	-3.664779000	1.039719000
8	-0.720826000	-3.149414000	1.464249000
1	-0.556387000	-3.625437000	2.301774000
d(OH/CN)			
46	-0.019269000	-0.489741000	-0.034980000
6	-0.130675000	-2.629671000	0.235790000
6	0.461557000	4.206789000	0.274709000
6	-4.447216000	-0.031610000	-0.383238000
6	-3.171696000	-0.590146000	-0.320190000
7	-2.064603000	0.141345000	-0.148457000
6	-2.189372000	1.499579000	-0.059055000
6	-3.431504000	2.117574000	-0.106064000
6	-4.581530000	1.343335000	-0.263760000
1	-3.048889000	-1.666712000	-0.400063000
1	-3.509301000	3.200610000	-0.027726000
1	-5.562909000	1.816799000	-0.300126000
1	-5.308726000	-0.683759000	-0.520293000
6	-0.927395000	2.265723000	0.057049000
7	0.183055000	1.513278000	0.004098000
6	1.424550000	2.022333000	0.078987000
6	1.596151000	3.397752000	0.216017000
6	-0.812392000	3.648947000	0.194442000
1	2.588471000	3.839630000	0.283777000
1	0.572974000	5.285615000	0.386569000
1	-1.693681000	4.285628000	0.243762000
6	2.505803000	1.012050000	-0.006560000
7	2.109050000	-0.293609000	-0.050285000
6	3.044474000	-1.246777000	-0.171158000
6	4.405851000	-0.963544000	-0.241588000
6	4.816193000	0.361677000	-0.180943000
6	3.849940000	1.360009000	-0.063941000
1	2.692149000	-2.276607000	-0.197914000
1	5.119937000	-1.780202000	-0.339442000
1	5.873620000	0.623077000	-0.229346000
1	4.147824000	2.406549000	-0.027662000

1	0.026402000	-2.650642000	-0.972645000
6	-0.008684000	-3.926433000	-1.253748000
1	-0.977520000	-3.974050000	-1.761098000
1	0.165524000	-4.714593000	-0.517437000
1	0.858124000	-3.771713000	-1.913267000
8	0.937028000	-3.133708000	0.938326000
1	1.177461000	-2.514586000	1.648198000
6	-1.424852000	-3.089236000	0.707334000
7	-2.457006000	-3.414933000	1.117887000

e(CH_3/Cl)

46	-0.027540000	-0.471601000	-0.104719000
6	-0.646486000	-2.557305000	0.033822000
6	1.090780000	4.130810000	0.254896000
6	-4.362619000	0.608047000	-0.289285000
6	-3.177407000	-0.119714000	-0.245078000
7	-1.970485000	0.450940000	-0.147460000
6	-1.899070000	1.810377000	-0.080140000
6	-3.044404000	2.598364000	-0.112408000
6	-4.294713000	1.993294000	-0.220865000
1	-3.207052000	-1.206874000	-0.264058000
1	-2.967145000	3.682535000	-0.055062000
1	-5.199643000	2.600652000	-0.248595000
1	-5.314074000	0.084181000	-0.369788000
6	-0.540413000	2.390282000	0.029780000
7	0.457259000	1.490257000	-0.001015000
6	1.751589000	1.833254000	0.084893000
6	2.103804000	3.176209000	0.217865000
6	-0.245044000	3.745913000	0.158068000
1	3.145441000	3.481611000	0.292713000
1	1.344194000	5.185941000	0.359942000
1	-1.033714000	4.495017000	0.187938000
6	2.695630000	0.695294000	0.007144000
7	2.141560000	-0.545353000	-0.122529000
6	2.967688000	-1.590805000	-0.245888000
6	4.355829000	-1.477952000	-0.228625000
6	4.920470000	-0.220374000	-0.074996000
6	4.072871000	0.879728000	0.039318000
1	2.508668000	-2.565799000	-0.365934000
1	4.967744000	-2.372689000	-0.334287000
1	6.002334000	-0.088395000	-0.050536000
1	4.490358000	1.878878000	0.148400000
1	-1.159686000	-2.230329000	-1.004145000
6	-1.738699000	-3.366365000	-1.362100000
17	-1.730414000	-2.586204000	1.422866000
1	-2.590249000	-2.780751000	-1.737861000
1	-2.062744000	-4.169891000	-0.698232000
1	-1.052068000	-3.682620000	-2.153623000
6	0.396044000	-3.642563000	0.101913000
1	1.077122000	-3.408857000	0.931831000
1	-0.057670000	-4.621603000	0.314975000
1	0.958611000	-3.711779000	-0.838196000

f(CH_3/COOH)

46	-0.007551000	-0.440892000	0.005642000
6	-0.783750000	-2.188992000	0.544866000
6	1.685430000	3.943929000	0.535260000
6	-4.084843000	1.166321000	-0.730028000
6	-3.014672000	0.295709000	-0.544225000
7	-1.784232000	0.718149000	-0.231141000
6	-1.551207000	2.058646000	-0.138784000
6	-2.578647000	2.980462000	-0.298088000
6	-3.866464000	2.530399000	-0.586549000
1	-3.149674000	-0.778262000	-0.673275000
1	-2.375816000	4.047408000	-0.219564000
1	-4.680931000	3.243270000	-0.717153000
1	-5.065221000	0.768516000	-0.988209000
6	-0.140222000	2.462333000	0.080433000
7	0.740235000	1.452239000	-0.014027000
6	2.059829000	1.606082000	0.175321000
6	2.573438000	2.871920000	0.452302000
6	0.316375000	3.750576000	0.351761000
1	3.638281000	3.027355000	0.616434000
1	2.064132000	4.941905000	0.757611000
1	-0.373126000	4.588582000	0.437574000
6	2.841046000	0.352232000	0.033946000
7	2.118445000	-0.798776000	-0.097512000
6	2.772170000	-1.930386000	-0.383567000
6	4.159140000	-2.004654000	-0.481749000
6	4.902175000	-0.849078000	-0.281537000
6	4.228843000	0.347136000	-0.037008000
1	2.165950000	-2.814535000	-0.569640000
1	4.632275000	-2.956970000	-0.717285000
1	5.990685000	-0.865884000	-0.340727000
1	4.786254000	1.276101000	0.072567000
1	-1.806767000	-2.924068000	-1.691830000
6	-1.114746000	-2.539612000	-2.457891000
1	-1.243108000	-1.454888000	-2.582121000
1	-1.348628000	-3.039106000	-3.406624000
1	-0.072097000	-2.763305000	-2.190606000
6	-2.181267000	-2.148701000	1.098400000
8	-2.344319000	-1.438670000	2.051498000

8	-3.050589000	-2.950476000	0.511057000
1	-3.901055000	-2.928011000	0.995880000
6	-0.111190000	-3.425222000	0.925598000
1	-0.130358000	-3.406627000	2.040816000
1	-0.735899000	-4.299510000	0.664716000
1	0.921943000	-3.543658000	0.599449000
g(CH_3/CN)			
46	0.010850000	-0.489691000	0.074166000
6	-0.110179000	-2.646336000	0.012330000
6	0.251183000	4.199265000	-0.465507000
6	-4.429919000	-0.238692000	0.492745000
6	-3.130285000	-0.737272000	0.414999000
7	-2.066867000	0.039833000	0.181645000
6	-2.260090000	1.385954000	0.048800000
6	-3.529265000	1.945261000	0.105499000
6	-4.634997000	1.122125000	0.322594000
1	-2.950428000	-1.802343000	0.537938000
1	-3.662067000	3.020001000	-0.006909000
1	-5.637401000	1.548299000	0.368205000
1	-5.254526000	-0.924621000	0.681777000
6	-1.038426000	2.208206000	-0.118143000
7	0.108468000	1.518694000	-0.025090000
6	1.322967000	2.081941000	-0.147449000
6	1.426241000	3.452257000	-0.369891000
6	-0.992645000	3.585257000	-0.340794000
1	2.394998000	3.937124000	-0.476312000
1	0.307748000	5.273274000	-0.644348000
1	-1.905066000	4.172697000	-0.425029000
6	2.451387000	1.128971000	-0.008087000
7	2.111303000	-0.184768000	0.126694000
6	3.079552000	-1.083431000	0.342441000
6	4.427916000	-0.746530000	0.397535000
6	4.784247000	0.586100000	0.225131000
6	3.781225000	1.533737000	0.029016000
1	2.761588000	-2.114452000	0.497618000
1	5.173795000	-1.519276000	0.578732000
1	5.830199000	0.892180000	0.258177000
1	4.037502000	2.586470000	-0.079211000
1	0.864529000	-2.584242000	-0.748161000
6	1.133689000	-3.749925000	-1.202388000
1	0.916104000	-3.513769000	-2.251065000
1	0.633564000	-4.652603000	-0.850321000
1	2.195317000	-3.713544000	-0.928849000
6	0.096012000	-3.319550000	1.351025000
1	-0.674994000	-2.922981000	2.030280000
1	-0.019560000	-4.412204000	1.312732000
1	1.071225000	-3.062059000	1.785411000
6	-1.283339000	-3.040396000	-0.733778000
7	-2.246227000	-3.328591000	-1.310213000
h(NH_2/Cl)			
46	-0.015597000	-0.459342000	-0.067326000
6	-0.354528000	-2.583469000	0.244969000
6	0.785731000	4.171250000	0.482111000
6	-4.377850000	0.344418000	-0.605863000
6	-3.153225000	-0.309113000	-0.491082000
7	-2.009041000	0.330785000	-0.222743000
6	-2.038474000	1.687720000	-0.086202000
6	-3.228395000	2.399744000	-0.172413000
6	-4.418623000	1.720505000	-0.429259000
1	-3.095347000	-1.388721000	-0.608335000
1	-3.231294000	3.481994000	-0.054272000
1	-5.359277000	2.266902000	-0.501614000
1	-5.275302000	-0.231420000	-0.827478000
6	-0.728278000	2.351581000	0.109913000
7	0.328087000	1.526944000	0.022840000
6	1.599132000	1.942879000	0.147205000
6	1.862141000	3.291213000	0.381921000
6	-0.522176000	3.710436000	0.342984000
1	2.881459000	3.655832000	0.494423000
1	0.968588000	5.229122000	0.672288000
1	-1.358865000	4.401730000	0.424103000
6	2.609311000	0.870404000	-0.012313000
7	2.124860000	-0.400635000	-0.132046000
6	2.988376000	-1.397625000	-0.369592000
6	4.363433000	-1.195205000	-0.468294000
6	4.864662000	0.089468000	-0.312312000
6	3.970679000	1.135947000	-0.086938000
1	2.559579000	-2.393366000	-0.466945000
1	5.016974000	-2.044299000	-0.664025000
1	5.935263000	0.285292000	-0.376533000
1	4.337171000	2.156246000	0.013034000
1	-0.499162000	-2.489452000	-0.946374000
6	-0.692881000	-3.768868000	-1.218117000
1	-1.616462000	-3.548944000	-1.769850000
1	-0.827526000	-4.617505000	-0.543752000
1	0.206435000	-3.854060000	-1.839786000
17	-1.860016000	-2.801879000	1.167196000
7	0.794731000	-3.192815000	0.747508000
1	1.219765000	-2.630358000	1.486267000
1	0.631057000	-4.134965000	1.114311000

i(NH_2/COOH)			
46	-0.000016000	-0.340318000	0.093336000
6	-0.528654000	-2.486180000	0.027220000
6	1.327979000	4.141835000	-0.492686000
6	-4.251134000	0.934058000	0.574154000
6	-3.104539000	0.145536000	0.490183000
7	-1.896756000	0.652239000	0.215804000
6	-1.774869000	2.003333000	0.058428000
6	-2.879504000	2.842864000	0.116504000
6	-4.140629000	2.301641000	0.367320000
1	-3.168540000	-0.926028000	0.676098000
1	-2.761397000	3.917233000	-0.015168000
1	-5.016445000	2.948891000	0.418466000
1	-5.206550000	0.465133000	0.806024000
6	-0.395411000	2.517568000	-0.121795000
7	0.559118000	1.581144000	-0.012329000
6	1.873116000	1.836326000	-0.142637000
6	2.294801000	3.141357000	-0.383656000
6	-0.026739000	3.840819000	-0.364864000
1	3.350627000	3.382251000	-0.494845000
1	1.635834000	5.169726000	-0.686078000
1	-0.774809000	4.625597000	-0.461845000
6	2.740518000	0.641845000	0.006001000
7	2.091377000	-0.549339000	0.155318000
6	2.801008000	-1.661925000	0.387194000
6	4.193042000	-1.658565000	0.436627000
6	4.865398000	-0.457720000	0.246174000
6	4.127555000	0.708253000	0.039022000
1	2.226401000	-2.572577000	0.570422000
1	4.727176000	-2.587920000	0.630047000
1	5.954638000	-0.418344000	0.275257000
1	4.635202000	1.664310000	-0.080162000
1	0.230775000	-2.311429000	-0.879748000
6	0.208420000	-3.582386000	-1.352514000
1	-0.146187000	-3.302886000	-2.349874000
1	-0.277761000	-4.480619000	-0.962397000
1	1.298273000	-3.651407000	-1.254242000
7	-0.060290000	-3.137013000	1.174595000
1	-0.024694000	-2.536755000	1.996225000
1	-0.615814000	-3.966326000	1.403055000
6	-1.965000000	-2.658900000	-0.376745000
8	-2.771799000	-3.170482000	0.349955000
8	-2.223207000	-2.148135000	-1.586043000
1	-3.152198000	-2.343072000	-1.813289000

j(NH_2/CN)			
46	-0.009129000	-0.472076000	0.036022000
6	0.068244000	-2.713067000	0.089000000
6	0.181829000	4.246777000	-0.172457000
6	-4.479853000	-0.291730000	0.120468000
6	-3.171094000	-0.775129000	0.097942000
7	-2.101762000	0.025590000	0.036887000
6	-2.305350000	1.377340000	0.004039000
6	-3.581564000	1.921856000	0.023023000
6	-4.690964000	1.077112000	0.080188000
1	-2.993214000	-1.846564000	0.128476000
1	-3.718521000	3.001518000	-0.002001000
1	-5.699020000	1.492054000	0.095850000
1	-5.305507000	-1.000458000	0.169637000
6	-1.090213000	2.218334000	-0.044831000
7	0.062471000	1.531312000	-0.005683000
6	1.272974000	2.117270000	-0.054776000
6	1.361880000	3.504074000	-0.138242000
6	-1.055746000	3.610603000	-0.127807000
1	2.326927000	4.005563000	-0.178512000
1	0.229097000	5.334034000	-0.238376000
1	-1.973620000	4.194172000	-0.162123000
6	2.413082000	1.173862000	-0.006233000
7	2.091885000	-0.150223000	0.074757000
6	3.080516000	-1.051636000	0.164329000
6	4.426305000	-0.693684000	0.152103000
6	4.760581000	0.650275000	0.050572000
6	3.737218000	1.594475000	-0.022543000
1	2.777397000	-2.091531000	0.286489000
1	5.188328000	-1.468198000	0.229251000
1	5.803367000	0.968372000	0.037535000
1	3.975921000	2.654702000	-0.087131000
1	0.575718000	-2.389092000	-0.946890000
6	0.883296000	-3.616297000	-1.410183000
1	0.215557000	-3.586886000	-2.278926000
1	0.871460000	-4.580262000	-0.897410000
1	1.906750000	-3.269514000	-1.606970000
7	0.907257000	-3.094597000	1.150659000
1	0.846673000	-2.475072000	1.957243000
1	0.776673000	-4.061733000	1.455165000
6	-1.217501000	-3.357310000	-0.058118000
7	-2.237834000	-3.901278000	-0.127647000

Pt carbenoid reactions

a(H/H)			
78	0.002630000	-0.714219000	-0.162727000
6	0.024305000	-2.643487000	-0.495092000

6	-0.052056000	3.987437000	0.341164000
6	4.396002000	-0.786286000	-0.207733000
6	3.061521000	-1.180215000	-0.235423000
7	2.049390000	-0.310127000	-0.128573000
6	2.328535000	1.023268000	0.013208000
6	3.639831000	1.476463000	0.047477000
6	4.689375000	0.563568000	-0.064362000
1	2.794590000	-2.229978000	-0.350869000
1	3.847521000	2.539575000	0.159383000
1	5.722764000	0.910282000	-0.040067000
1	5.180473000	-1.536130000	-0.300447000
6	1.156701000	1.934042000	0.120746000
7	-0.022509000	1.297977000	0.062301000
6	-1.215069000	1.906967000	0.127575000
6	-1.260960000	3.292085000	0.272176000
6	1.172124000	3.319456000	0.265488000
1	-2.206353000	3.828865000	0.329466000
1	-0.063906000	5.071912000	0.453965000
1	2.105428000	3.877468000	0.318026000
6	-2.365372000	0.969206000	0.024139000
7	-2.055417000	-0.356943000	-0.123665000
6	-3.048154000	-1.248857000	-0.229905000
6	-4.391435000	-0.885431000	-0.195078000
6	-4.715475000	0.456215000	-0.044040000
6	-3.686246000	1.392285000	0.066540000
1	-2.759223000	-2.291646000	-0.350937000
1	-5.158566000	-1.652940000	-0.288318000
1	-5.756406000	0.778971000	-0.013176000
1	-3.917404000	2.449948000	0.183946000
1	0.554015000	-3.406676000	0.613933000
6	0.165633000	-3.553435000	1.699339000
1	0.858048000	-3.117582000	-1.043785000
1	0.880368000	-2.977626000	2.297181000
1	0.263279000	-4.640196000	1.816661000
1	-0.860557000	-3.203793000	1.824843000
1	-0.885035000	-3.247445000	-0.624814000
b(OH/Cl)			
78	-0.015693000	-0.384987000	-0.091826000
6	-0.441249000	-2.477061000	0.116744000
6	0.897887000	4.227082000	0.338752000
6	-4.355488000	0.420889000	-0.377289000
6	-3.130886000	-0.236367000	-0.315534000
7	-1.965828000	0.406542000	-0.153847000
6	-1.981566000	1.770517000	-0.059357000
6	-3.171930000	2.483820000	-0.103415000
6	-4.378677000	1.804234000	-0.262010000
1	-3.088605000	-1.320653000	-0.380638000
1	-3.161225000	3.569347000	-0.021548000
1	-5.319458000	2.353862000	-0.299615000
1	-5.268177000	-0.158321000	-0.510039000
6	-0.661312000	2.429567000	0.071613000
7	0.377998000	1.579491000	0.015197000
6	1.659170000	1.967107000	0.113487000
6	1.953714000	3.319189000	0.279203000
6	-0.423506000	3.791425000	0.234424000
1	2.981648000	3.666109000	0.365813000
1	1.106418000	5.288991000	0.471016000
1	-1.242596000	4.506207000	0.286453000
6	2.627507000	0.851886000	0.017163000
7	2.092240000	-0.403173000	-0.100525000
6	2.926954000	-1.441271000	-0.246781000
6	4.312934000	-1.295474000	-0.262758000
6	4.860608000	-0.030277000	-0.118997000
6	3.998812000	1.058228000	0.018535000
1	2.465970000	-2.418288000	-0.342944000
1	4.938118000	-2.178780000	-0.385098000
1	5.940535000	0.118759000	-0.120303000
1	4.399552000	2.065515000	0.117975000
1	-0.707696000	-2.136307000	-1.007306000
6	-1.173282000	-3.388841000	-1.315728000
17	-1.682773000	-2.682511000	1.355717000
1	-2.121007000	-2.985740000	-1.696975000
1	-1.342959000	-4.312045000	-0.753734000
1	-0.409217000	-3.513078000	-2.094492000
8	0.690948000	-3.179808000	0.343246000
1	0.483317000	-4.083390000	0.646606000
c(OH/COOH)			
78	0.000325000	-0.256879000	-0.116086000
6	0.278591000	-2.365913000	-0.553528000
6	-0.243470000	4.438065000	-0.087614000
6	-4.360731000	-0.625620000	0.292071000
6	-3.008908000	-0.934566000	0.177994000
7	-2.064741000	0.004425000	0.026548000
6	-2.430959000	1.321557000	0.043128000
6	-3.764635000	1.693043000	0.147307000
6	-4.746319000	0.708746000	0.259481000
1	-2.657819000	-1.962869000	0.244877000
1	-4.039306000	2.746732000	0.157539000
1	-5.797037000	0.988168000	0.340874000
1	-5.088028000	-1.427471000	0.411199000

6	-1.317757000	2.300272000	-0.000445000
7	-0.100491000	1.730680000	0.037788000
6	1.049619000	2.422598000	-0.001495000
6	1.003146000	3.814527000	-0.061000000
6	-1.419938000	3.686928000	-0.059517000
1	1.915747000	4.406916000	-0.096322000
1	-0.300562000	5.525603000	-0.138595000
1	-2.389275000	4.181269000	-0.092975000
6	2.258812000	1.566965000	0.042461000
7	2.034749000	0.217490000	0.010524000
6	3.072273000	-0.614495000	0.164970000
6	4.380792000	-0.155839000	0.306110000
6	4.622440000	1.209746000	0.284680000
6	3.540138000	2.082846000	0.162114000
1	2.843826000	-1.676437000	0.182395000
1	5.186973000	-0.877314000	0.430770000
1	5.635406000	1.600826000	0.381522000
1	3.697803000	3.160129000	0.178554000
1	-0.716824000	-2.102872000	-1.146674000
6	-0.973187000	-3.412818000	-1.478823000
1	-1.848605000	-3.634256000	-0.863086000
1	-0.290860000	-4.262156000	-1.568731000
1	-1.210851000	-3.013247000	-2.476215000
8	1.385075000	-2.486672000	-1.332420000
1	1.806186000	-3.349624000	-1.149988000
6	0.326235000	-3.137902000	0.740069000
8	1.347589000	-3.667629000	1.077684000
8	-0.826016000	-3.131396000	1.408700000
1	-0.708803000	-3.638281000	2.236029000
	d(OH/CN)		
78	-0.016161000	-0.418778000	-0.051469000
6	-0.230208000	-2.510160000	0.250111000
6	0.590414000	4.242354000	0.346641000
6	-4.389198000	0.100987000	-0.461852000
6	-3.123890000	-0.475527000	-0.385565000
7	-2.011151000	0.238780000	-0.168338000
6	-2.121186000	1.599593000	-0.058154000
6	-3.354721000	2.231341000	-0.114234000
6	-4.510461000	1.474203000	-0.309176000
1	-3.010032000	-1.550449000	-0.491843000
1	-3.418789000	3.313848000	-0.017129000
1	-5.485758000	1.959470000	-0.352479000
1	-5.255172000	-0.536596000	-0.634375000
6	-0.849390000	2.347002000	0.079483000
7	0.243684000	1.570243000	0.008919000

6	1.498364000	2.040197000	0.104230000
6	1.705317000	3.406612000	0.274493000
6	-0.699605000	3.721696000	0.250371000
1	2.708413000	3.820607000	0.359165000
1	0.729437000	5.314936000	0.484695000
1	-1.563616000	4.380507000	0.314756000
6	2.537400000	0.988161000	0.003718000
7	2.084211000	-0.301658000	-0.065269000
6	2.979348000	-1.289978000	-0.217651000
6	4.350306000	-1.057929000	-0.287950000
6	4.817414000	0.245705000	-0.193577000
6	3.893159000	1.280262000	-0.049320000
1	2.582214000	-2.301142000	-0.267761000
1	5.028344000	-1.901247000	-0.412228000
1	5.884708000	0.463683000	-0.239271000
1	4.231360000	2.313585000	0.009096000
1	-0.177640000	-2.476573000	-0.959493000
6	-0.362371000	-3.766589000	-1.199842000
1	-1.364661000	-3.716815000	-1.637164000
1	-0.207918000	-4.594586000	-0.503595000
1	0.460259000	-3.686319000	-1.926563000
8	0.862118000	-3.107859000	0.847704000
1	1.079176000	-2.614483000	1.657749000
6	-1.499576000	-2.848817000	0.879822000
7	-2.494374000	-3.083869000	1.421839000
	e(CH_3/Cl)		
78	-0.016455000	-0.394642000	-0.104070000
6	-0.579163000	-2.458615000	0.166562000
6	1.032837000	4.186181000	0.421683000
6	-4.323057000	0.570104000	-0.458979000
6	-3.124399000	-0.130508000	-0.371892000
7	-1.940020000	0.467466000	-0.182749000
6	-1.910458000	1.830306000	-0.084321000
6	-3.072990000	2.587004000	-0.151399000
6	-4.299725000	1.952922000	-0.339241000
1	-3.122162000	-1.214965000	-0.434310000
1	-3.024320000	3.671189000	-0.066597000
1	-5.218938000	2.536325000	-0.395759000
1	-5.252544000	0.023984000	-0.613395000
6	-0.572031000	2.442047000	0.076940000
7	0.440570000	1.561416000	0.009424000
6	1.729041000	1.911576000	0.142959000
6	2.061396000	3.248155000	0.354064000
6	-0.297346000	3.791745000	0.282395000
1	3.097195000	3.560995000	0.470674000

1	1.269966000	5.236919000	0.589680000
1	-1.096924000	4.527472000	0.344374000
6	2.669443000	0.775776000	0.021500000
7	2.105244000	-0.460585000	-0.138654000
6	2.924129000	-1.499934000	-0.349329000
6	4.311543000	-1.387643000	-0.370474000
6	4.886378000	-0.142042000	-0.165550000
6	4.046278000	0.953984000	0.023166000
1	2.456058000	-2.462801000	-0.512134000
1	4.916111000	-2.276243000	-0.546577000
1	5.969068000	-0.014671000	-0.166180000
1	4.467065000	1.948748000	0.158526000
1	-0.965162000	-2.208577000	-0.949132000
6	-1.478746000	-3.402196000	-1.227013000
17	-1.811056000	-2.462645000	1.441359000
1	-2.380809000	-2.916485000	-1.624481000
1	-1.735747000	-4.235997000	-0.570755000
1	-0.757402000	-3.667601000	-2.008311000
6	0.489117000	-3.491888000	0.449666000
1	1.165007000	-3.102782000	1.223093000
1	0.035205000	-4.417226000	0.830928000
1	1.055707000	-3.741208000	-0.455834000

f(CH_3/COOH)

78	0.003780000	-0.386436000	-0.027470000
6	-0.785411000	-2.038725000	0.555500000
6	1.780491000	3.967442000	0.498736000
6	-4.035564000	1.225166000	-0.685373000
6	-2.967702000	0.350305000	-0.511446000
7	-1.728250000	0.774044000	-0.228144000
6	-1.487114000	2.118570000	-0.153022000
6	-2.514866000	3.038779000	-0.303349000
6	-3.809896000	2.589753000	-0.563020000
1	-3.106656000	-0.724850000	-0.619839000
1	-2.305620000	4.105491000	-0.238799000
1	-4.624301000	3.304066000	-0.686136000
1	-5.022238000	0.827124000	-0.917905000
6	-0.071817000	2.521179000	0.047068000
7	0.795820000	1.500132000	-0.046399000
6	2.115481000	1.623196000	0.156486000
6	2.651821000	2.879324000	0.431085000
6	0.406439000	3.801329000	0.313060000
1	3.717382000	3.017228000	0.606319000
1	2.176506000	4.959662000	0.717123000
1	-0.266241000	4.653237000	0.396458000
6	2.851055000	0.339609000	0.037233000

7	2.087050000	-0.789983000	-0.096660000
6	2.706541000	-1.943369000	-0.379298000
6	4.091169000	-2.062644000	-0.459502000
6	4.872456000	-0.935628000	-0.247441000
6	4.236696000	0.283940000	-0.015070000
1	2.073450000	-2.803645000	-0.574546000
1	4.532168000	-3.031516000	-0.689849000
1	5.960460000	-0.990834000	-0.290281000
1	4.822133000	1.195090000	0.098540000
1	-1.582074000	-1.344875000	-2.648658000
6	-1.592700000	-2.437798000	-2.532426000
1	-1.986609000	-2.892478000	-3.449609000
1	-2.240328000	-2.731667000	-1.691873000
1	-0.567429000	-2.798071000	-2.368532000
6	-2.117579000	-1.909506000	1.275604000
8	-2.231998000	-1.115714000	2.165560000
8	-3.028824000	-2.771414000	0.841697000
1	-3.831430000	-2.703240000	1.397161000
6	-0.247202000	-3.390050000	0.740002000
1	-0.349305000	-3.682607000	1.802718000
1	-0.954279000	-4.065203000	0.214543000
1	0.770004000	-3.568482000	0.397206000

g(CH_3/CN)

78	0.009378000	-0.411413000	0.061996000
6	-0.318949000	-2.545711000	-0.003243000
6	0.461532000	4.254779000	-0.402445000
6	-4.381940000	-0.035872000	0.458031000
6	-3.098542000	-0.572189000	0.383408000
7	-2.009983000	0.177172000	0.164906000
6	-2.164287000	1.532671000	0.049709000
6	-3.416801000	2.125822000	0.104862000
6	-4.547383000	1.332288000	0.302832000
1	-2.950903000	-1.642938000	0.494607000
1	-3.515625000	3.205454000	0.004877000
1	-5.537719000	1.785997000	0.345886000
1	-5.227136000	-0.700225000	0.632313000
6	-0.916781000	2.318038000	-0.097584000
7	0.200013000	1.577412000	-0.022048000
6	1.438944000	2.085686000	-0.138514000
6	1.602703000	3.454205000	-0.330227000
6	-0.809648000	3.695143000	-0.288316000
1	2.591996000	3.897108000	-0.430636000
1	0.565941000	5.329080000	-0.556034000
1	-1.694831000	4.324909000	-0.356110000
6	2.510497000	1.067436000	-0.030057000

7	2.091779000	-0.228220000	0.089797000
6	3.010974000	-1.185305000	0.277668000
6	4.376498000	-0.927024000	0.315619000
6	4.810122000	0.383819000	0.157289000
6	3.861229000	1.391276000	-0.008035000
1	2.638122000	-2.197656000	0.421592000
1	5.076777000	-1.746400000	0.471860000
1	5.872805000	0.626738000	0.176970000
1	4.174962000	2.429392000	-0.106019000
1	0.738898000	-2.545629000	-0.586437000
6	0.922136000	-3.829311000	-0.805423000
1	1.338155000	-3.555713000	-1.787466000
1	0.142057000	-4.586320000	-0.903639000
1	1.665844000	-4.074283000	-0.041552000
6	-0.455020000	-3.174815000	1.373626000
1	-1.261785000	-2.653648000	1.908419000
1	-0.704396000	-4.244383000	1.328719000
1	0.462816000	-3.029169000	1.958622000
6	-1.400386000	-2.788739000	-0.942854000
7	-2.284215000	-2.958551000	-1.671185000
h(NH_2/Cl)			
78	-0.012925000	-0.385640000	-0.061854000
6	-0.346157000	-2.472462000	0.274910000
6	0.790828000	4.229921000	0.493127000
6	-4.341407000	0.360873000	-0.619469000
6	-3.111201000	-0.279760000	-0.498527000
7	-1.977774000	0.371870000	-0.204717000
6	-2.026998000	1.731743000	-0.065333000
6	-3.225183000	2.426227000	-0.155135000
6	-4.404773000	1.733419000	-0.426015000
1	-3.037293000	-1.355486000	-0.630369000
1	-3.240147000	3.507894000	-0.032124000
1	-5.352434000	2.267215000	-0.500596000
1	-5.227359000	-0.225319000	-0.859338000
6	-0.725608000	2.413209000	0.125027000
7	0.332516000	1.592144000	0.025731000
6	1.604660000	2.003990000	0.150663000
6	1.868576000	3.350939000	0.387449000
6	-0.519300000	3.770315000	0.361881000
1	2.888033000	3.715545000	0.499410000
1	0.974448000	5.287320000	0.685145000
1	-1.355500000	4.461110000	0.452806000
6	2.598759000	0.916816000	-0.004774000
7	2.092865000	-0.350480000	-0.116148000
6	2.942329000	-1.360982000	-0.356590000
6	4.318855000	-1.175525000	-0.461704000
6	4.840725000	0.101006000	-0.310574000
6	3.962252000	1.160831000	-0.084951000
1	2.498576000	-2.350132000	-0.441716000
1	4.958333000	-2.035085000	-0.658003000
1	5.913851000	0.280638000	-0.379281000
1	4.341234000	2.177136000	0.009575000
1	-0.437422000	-2.329267000	-0.926749000
6	-0.619948000	-3.615021000	-1.195738000
1	-1.529087000	-3.393742000	-1.770845000
1	-0.765892000	-4.491722000	-0.560452000
1	0.292632000	-3.681545000	-1.801325000
17	-1.879427000	-2.742491000	1.155054000
7	0.786591000	-3.087919000	0.819088000
1	1.153135000	-2.544427000	1.602339000
1	0.605434000	-4.040413000	1.147040000
i(NH_2/COOH)			
78	-0.024625000	-0.294157000	-0.085043000
6	-0.771592000	-2.306484000	0.211065000
6	1.756015000	4.050274000	0.384297000
6	-4.106211000	1.347401000	-0.530254000
6	-3.030683000	0.464946000	-0.474735000
7	-1.778260000	0.861376000	-0.213879000
6	-1.540803000	2.198494000	-0.049521000
6	-2.572066000	3.126546000	-0.078609000
6	-3.878838000	2.697948000	-0.308782000
1	-3.178840000	-0.593687000	-0.667689000
1	-2.359130000	4.184644000	0.063873000
1	-4.698226000	3.416567000	-0.332819000
1	-5.102107000	0.963928000	-0.747250000
6	-0.120965000	2.590252000	0.100608000
7	0.734674000	1.559427000	0.006190000
6	2.068008000	1.693031000	0.097196000
6	2.618152000	2.958672000	0.286127000
6	0.375365000	3.877309000	0.293651000
1	3.694309000	3.099679000	0.367480000
1	2.165493000	5.048862000	0.538475000
1	-0.291944000	4.733005000	0.377359000
6	2.805313000	0.413620000	-0.016166000
7	2.039558000	-0.719164000	-0.072945000
6	2.655891000	-1.903281000	-0.204830000
6	4.041262000	-2.024814000	-0.282386000
6	4.822843000	-0.880531000	-0.214802000
6	4.190278000	0.355035000	-0.078347000
1	2.012423000	-2.779928000	-0.206131000

1	4.484075000	-3.014045000	-0.389476000
1	5.910101000	-0.939201000	-0.269209000
1	4.778242000	1.270144000	-0.031626000
1	-0.540101000	-2.257148000	-0.980871000
6	-0.781143000	-3.527227000	-1.240747000
1	-1.456337000	-3.276317000	-2.066854000
1	-1.192168000	-4.337479000	-0.636002000
1	0.253682000	-3.723040000	-1.542918000
7	0.146871000	-2.966660000	1.062524000
1	0.460998000	-2.352120000	1.813729000
1	-0.253812000	-3.802442000	1.495150000
6	-2.213642000	-2.323636000	0.687362000
8	-2.479006000	-1.990818000	1.805070000
8	-3.106914000	-2.746545000	-0.220721000
1	-3.981548000	-2.788485000	0.213324000
j(NH_2/CN)			
78	-0.015277000	-0.415003000	-0.039929000
6	-0.242607000	-2.541598000	0.292666000
6	0.597762000	4.238010000	0.374437000
6	-4.386277000	0.122688000	-0.499173000
6	-3.123681000	-0.459993000	-0.416044000
7	-2.011877000	0.246201000	-0.170765000
6	-2.119689000	1.605898000	-0.047509000
6	-3.349875000	2.243361000	-0.107413000
6	-4.506177000	1.493030000	-0.324710000
1	-3.013046000	-1.532812000	-0.540805000
1	-3.410714000	3.324898000	0.001610000
1	-5.479450000	1.981997000	-0.371099000
1	-5.251256000	-0.509789000	-0.694075000
6	-0.846463000	2.346878000	0.097560000
7	0.244714000	1.567637000	0.014739000
6	1.501010000	2.036380000	0.107489000
6	1.710346000	3.400861000	0.288119000
6	-0.692921000	3.719611000	0.280225000
1	2.714913000	3.811531000	0.371198000
1	0.739169000	5.309013000	0.521819000
1	-1.556189000	4.378215000	0.355204000
6	2.538884000	0.986253000	-0.007934000
7	2.084441000	-0.303439000	-0.069186000
6	2.976672000	-1.291538000	-0.240110000
6	4.346147000	-1.057574000	-0.335440000
6	4.814754000	0.245689000	-0.247634000
6	3.892955000	1.279765000	-0.084580000
1	2.576091000	-2.302675000	-0.270925000
1	5.022262000	-1.900253000	-0.473509000

1	5.880916000	0.464168000	-0.312750000
1	4.231405000	2.313282000	-0.031970000
1	-0.137214000	-2.434189000	-0.912950000
6	-0.250356000	-3.724861000	-1.210305000
1	-1.132183000	-3.588217000	-1.848571000
1	-0.333410000	-4.612990000	-0.580370000
1	0.709381000	-3.691830000	-1.743469000
7	0.857715000	-3.055137000	1.013222000
1	1.164331000	-2.409328000	1.741085000
1	0.665413000	-3.963230000	1.442510000
6	-1.568760000	-2.872097000	0.789217000
7	-2.596299000	-3.154136000	1.241751000
Cu carbenoid reactions			
a(H/H)			
29	-0.895393000	-1.143255000	-0.039030000
6	-2.229977000	-2.534345000	-0.054729000
1	-2.614831000	-3.061153000	-0.946556000
1	-2.676382000	-3.021259000	0.830756000
6	3.433882000	-1.823394000	0.029663000
6	0.613466000	1.346722000	-0.003079000
6	0.934330000	2.704290000	0.007943000
6	-0.091647000	3.644963000	-0.001597000
6	-1.411774000	3.208889000	-0.021136000
6	-1.650181000	1.838356000	-0.031480000
7	-0.668030000	0.930349000	-0.023449000
1	1.970443000	3.034759000	0.024273000
1	0.143000000	4.709474000	0.006840000
1	-2.245463000	3.909107000	-0.028242000
1	-2.667416000	1.445362000	-0.045638000
6	1.650283000	0.271367000	0.008405000
6	3.018857000	0.540915000	0.029369000
6	3.919572000	-0.520460000	0.040081000
6	2.055484000	-2.010014000	0.008570000
7	1.186253000	-0.993287000	-0.001741000
1	3.388739000	1.563755000	0.037261000
1	4.992164000	-0.326581000	0.056535000
1	4.102091000	-2.683004000	0.037497000
1	1.625903000	-3.012668000	-0.000315000
1	-4.212274000	-1.838136000	-0.361501000
6	-4.508706000	-0.892318000	0.129729000
1	-4.496537000	-0.097729000	-0.626287000
1	-3.843384000	-0.641564000	0.965671000
1	-5.528757000	-1.035215000	0.507480000
b(OH/Cl)			
29	-0.712163000	-0.373763000	-0.193058000

6	-2.790852000	-0.047024000	-0.200110000
6	2.579847000	-3.242357000	-0.001337000
6	1.896252000	0.953175000	0.005626000
6	2.902981000	1.918112000	0.067801000
6	2.549658000	3.264943000	0.063289000
6	1.205945000	3.616535000	-0.005720000
6	0.263115000	2.594357000	-0.068812000
7	0.599897000	1.301837000	-0.061636000
1	3.952592000	1.637006000	0.116013000
1	3.323143000	4.031592000	0.111835000
1	0.888286000	4.657981000	-0.012856000
1	-0.805873000	2.806725000	-0.131398000
6	2.183456000	-0.516528000	0.006538000
6	3.479715000	-1.022005000	0.118784000
6	3.679186000	-2.398988000	0.113994000
6	1.319733000	-2.664036000	-0.106623000
7	1.121277000	-1.340264000	-0.104285000
1	4.332773000	-0.354265000	0.213898000
1	4.686704000	-2.805781000	0.201740000
1	2.687212000	-4.325792000	-0.008367000
1	0.427226000	-3.284908000	-0.195673000
1	-2.826330000	-0.967062000	-0.961441000
6	-4.149140000	-1.062092000	-1.205680000
1	-4.210950000	-2.126979000	-0.948792000
1	-4.047382000	-0.859149000	-2.279092000
1	-4.956279000	-0.490325000	-0.742830000
17	-3.382247000	-0.368566000	1.434834000
8	-3.020318000	1.197305000	-0.665235000
1	-3.905257000	1.511686000	-0.399698000
c(OH/COOH)			
29	0.565676000	-0.242395000	0.246187000
6	2.607131000	0.225789000	0.329625000
6	-1.653241000	3.584415000	-0.069989000
6	-2.309936000	-0.611023000	-0.027931000
6	-3.559135000	-1.219405000	-0.161585000
6	-3.649405000	-2.607628000	-0.133851000
6	-2.490289000	-3.359126000	0.025255000
6	-1.282571000	-2.680861000	0.149299000
7	-1.189652000	-1.345928000	0.124940000
1	-4.459298000	-0.622888000	-0.290705000
1	-4.619117000	-3.094678000	-0.237700000
1	-2.512334000	-4.447341000	0.051895000
1	-0.345594000	-3.226036000	0.272611000
6	-2.137863000	0.876336000	-0.048503000
6	-3.213664000	1.759535000	-0.152070000

6	-2.964041000	3.129577000	-0.163869000
6	-0.636528000	2.638790000	0.033500000
7	-0.874114000	1.324443000	0.042413000
1	-4.237262000	1.397773000	-0.219507000
1	-3.792282000	3.833802000	-0.244270000
1	-1.416140000	4.647159000	-0.074599000
1	0.411820000	2.932942000	0.115710000
1	2.632194000	-0.503961000	1.315358000
6	3.896661000	-0.631712000	1.661405000
1	4.067641000	-1.687767000	1.430644000
1	3.637919000	-0.433229000	2.709964000
1	4.694282000	0.029502000	1.317279000
6	3.390322000	-0.279302000	-0.848860000
8	4.037013000	0.475939000	-1.528188000
8	3.254024000	-1.589577000	-1.061655000
1	3.790851000	-1.819279000	-1.842056000
8	2.787700000	1.537657000	0.561803000
1	3.574545000	1.829372000	0.054251000
d(OH/CN)			
29	0.778989000	-0.493268000	0.039954000
6	2.889362000	-0.613922000	0.233251000
6	-2.749027000	-3.090662000	-0.065441000
6	-1.718368000	1.034631000	-0.008072000
6	-2.649078000	2.073974000	-0.003776000
6	-2.195383000	3.390703000	0.008213000
6	-0.827870000	3.636692000	0.014156000
6	0.038324000	2.546200000	0.011202000
7	-0.394757000	1.280757000	0.001745000
1	-3.718021000	1.874002000	-0.008506000
1	-2.909953000	4.213965000	0.012102000
1	-0.428319000	4.649546000	0.021403000
1	1.121071000	2.687852000	0.011969000
6	-2.125970000	-0.405964000	-0.026768000
6	-3.464932000	-0.800904000	-0.062743000
6	-3.778766000	-2.155910000	-0.081772000
6	-1.441542000	-2.620541000	-0.030344000
7	-1.132771000	-1.317796000	-0.011178000
1	-4.264226000	-0.063919000	-0.077896000
1	-4.820648000	-2.475270000	-0.110035000
1	-2.946447000	-4.161266000	-0.080022000
1	-0.601022000	-3.316123000	-0.017656000
1	2.880065000	-1.336459000	-0.748575000
6	4.140461000	-1.374552000	-1.138193000
1	4.117266000	-0.751072000	-2.037401000
1	4.025106000	-2.450844000	-1.330347000

1	4.977724000	-1.170082000	-0.469080000
8	3.440364000	-1.107100000	1.372160000
1	2.823298000	-1.725060000	1.787998000
6	3.253728000	0.758874000	-0.046806000
7	3.442510000	1.881981000	-0.263047000
e(CH_3/Cl)			
29	0.700062000	-0.461724000	0.084861000
6	2.770833000	-0.397036000	0.233382000
6	-2.746588000	-3.155380000	-0.089126000
6	-1.860486000	0.999304000	0.007715000
6	-2.819050000	2.012498000	-0.049487000
6	-2.402957000	3.340668000	-0.016962000
6	-1.044614000	3.625129000	0.068586000
6	-0.152825000	2.557321000	0.117017000
7	-0.548428000	1.281680000	0.089199000
1	-3.879378000	1.782016000	-0.124223000
1	-3.138172000	4.144303000	-0.061283000
1	-0.675747000	4.649277000	0.094582000
1	0.924593000	2.724225000	0.175829000
6	-2.220369000	-0.453109000	-0.024224000
6	-3.544933000	-0.893771000	-0.047133000
6	-3.809859000	-2.259186000	-0.080680000
6	-1.455837000	-2.638804000	-0.060370000
7	-1.194120000	-1.326746000	-0.028798000
1	-4.369690000	-0.185000000	-0.034688000
1	-4.839651000	-2.616278000	-0.098543000
1	-2.905265000	-4.232204000	-0.115315000
1	-0.590025000	-3.302664000	-0.062320000
1	2.820635000	-1.435179000	-0.466500000
6	4.003976000	-1.840710000	-0.642392000
1	3.873074000	-1.976536000	-1.724295000
1	3.969363000	-2.772024000	-0.065798000
1	4.865073000	-1.214868000	-0.410813000
17	3.333075000	0.994604000	-0.706222000
6	3.388566000	-0.461920000	1.603503000
1	4.474577000	-0.280849000	1.614716000
1	2.907907000	0.328001000	2.200728000
1	3.161330000	-1.425055000	2.080962000
f(CH_3/COOH)			
29	0.550105000	-0.473057000	0.185912000
6	2.563768000	-0.348354000	0.437674000
6	-1.159454000	3.610276000	0.094462000
6	-2.354102000	-0.460421000	-0.057011000
6	-3.675361000	-0.897956000	-0.164368000
6	-3.940605000	-2.262830000	-0.215843000

6	-2.881131000	-3.161778000	-0.159115000
6	-1.593098000	-2.648935000	-0.051548000
7	-1.331350000	-1.337454000	-0.001547000
1	-4.496859000	-0.186634000	-0.208575000
1	-4.968027000	-2.617356000	-0.299915000
1	-3.040645000	-4.238129000	-0.196634000
1	-0.729750000	-3.314206000	-0.003125000
6	-1.992158000	0.990815000	-0.001457000
6	-2.944568000	2.009511000	-0.049898000
6	-2.518956000	3.334731000	0.000623000
6	-0.271163000	2.538417000	0.137973000
7	-0.679640000	1.266428000	0.093981000
1	-4.006471000	1.786623000	-0.126450000
1	-3.249200000	4.143351000	-0.035571000
1	-0.785458000	4.632201000	0.132551000
1	0.808818000	2.691376000	0.200914000
1	2.900154000	-1.677568000	-0.121027000
6	3.888747000	-2.281634000	-0.117841000
1	3.808427000	-2.832182000	-1.062957000
1	3.823243000	-2.928240000	0.763677000
1	4.769109000	-1.637153000	-0.106991000
6	3.144136000	-0.233201000	1.808822000
1	2.741948000	-0.977974000	2.504214000
1	4.243362000	-0.205613000	1.849791000
1	2.786587000	0.761191000	2.137232000
6	3.128167000	0.611151000	-0.551192000
8	2.960533000	1.790785000	-0.344079000
8	3.692315000	0.104644000	-1.644874000
1	3.930547000	0.853964000	-2.221677000
g(CH_3/CN)			
29	0.775428000	-0.475988000	0.095482000
6	2.832975000	-0.488681000	0.287052000
6	-0.910123000	3.634077000	-0.001319000
6	-2.137253000	-0.429957000	-0.025374000
6	-3.467654000	-0.853200000	-0.038434000
6	-3.751410000	-2.215125000	-0.052459000
6	-2.700854000	-3.126427000	-0.052196000
6	-1.403152000	-2.627767000	-0.034131000
7	-1.123624000	-1.318724000	-0.020821000
1	-4.282657000	-0.133232000	-0.032873000
1	-4.786158000	-2.557929000	-0.062016000
1	-2.874675000	-4.201163000	-0.063241000
1	-0.546418000	-3.303335000	-0.029690000
6	-1.758090000	1.018018000	-0.015518000
6	-2.704041000	2.041387000	-0.088308000

6	-2.271624000	3.365088000	-0.078889000
6	-0.029384000	2.557492000	0.064718000
7	-0.441815000	1.285829000	0.060029000
1	-3.767203000	1.822721000	-0.157890000
1	-2.997297000	4.176608000	-0.135494000
1	-0.527694000	4.653535000	0.005481000
1	1.050059000	2.716436000	0.116481000
1	3.010939000	-1.207246000	-0.883567000
6	4.041197000	-1.417866000	-1.441089000
1	3.834204000	-0.973233000	-2.421783000
1	4.048275000	-2.514659000	-1.441116000
1	4.923652000	-0.987967000	-0.966103000
6	3.286673000	0.841377000	-0.025143000
7	3.536420000	1.956948000	-0.233174000
6	3.559302000	-1.167887000	1.410151000
1	3.165781000	-0.676922000	2.319240000
1	3.295437000	-2.229776000	1.477640000
1	4.650092000	-1.031736000	1.409344000
h(NH_2/Cl)			
29	0.706646000	-0.464828000	0.098544000
6	2.803584000	-0.475093000	0.263646000
6	-1.004444000	3.629561000	0.070410000
6	-2.210617000	-0.439906000	-0.027616000
6	-3.538611000	-0.869899000	-0.050209000
6	-3.814161000	-2.233286000	-0.085330000
6	-2.758119000	-3.137992000	-0.095109000
6	-1.463160000	-2.632011000	-0.065351000
7	-1.191664000	-1.321931000	-0.032557000
1	-4.357785000	-0.154721000	-0.034823000
1	-4.846758000	-2.582210000	-0.102844000
1	-2.925424000	-4.213482000	-0.122519000
1	-0.602471000	-3.302553000	-0.067160000
6	-1.838978000	1.009632000	0.005366000
6	-2.788519000	2.029786000	-0.072673000
6	-2.363135000	3.354998000	-0.037703000
6	-0.121184000	2.555726000	0.137416000
7	-0.526036000	1.282641000	0.107813000
1	-3.848836000	1.806648000	-0.167227000
1	-3.091430000	4.163854000	-0.098443000
1	-0.628336000	4.651007000	0.098708000
1	0.956329000	2.714246000	0.212378000
1	2.698325000	-1.425273000	-0.480732000
6	3.951545000	-1.751306000	-0.717826000
1	3.830478000	-1.747371000	-1.808783000
1	3.879260000	-2.738405000	-0.245904000
1	4.846844000	-1.208195000	-0.412344000
17	3.315708000	0.997107000	-0.634387000
7	3.317296000	-0.664383000	1.526610000
1	4.239921000	-0.267908000	1.708720000
1	2.667727000	-0.482002000	2.284577000
i(NH_2/COOH)			
29	-0.528877000	-0.669506000	-0.215280000
6	-2.617007000	-0.540949000	-0.492227000
6	0.672468000	3.616921000	-0.107485000
6	2.337088000	-0.288074000	0.034132000
6	3.698356000	-0.559011000	0.183817000
6	4.132848000	-1.880174000	0.223408000
6	3.198750000	-2.904377000	0.113898000
6	1.860174000	-2.556229000	-0.028415000
7	1.436176000	-1.287006000	-0.067305000
1	4.420839000	0.248568000	0.275838000
1	5.193335000	-2.103575000	0.340788000
1	3.491798000	-3.952613000	0.139578000
1	1.090171000	-3.324066000	-0.115283000
6	1.802691000	1.110379000	-0.016937000
6	2.632568000	2.232695000	-0.002457000
6	2.056645000	3.499874000	-0.048342000
6	-0.086514000	2.449426000	-0.123647000
7	0.465283000	1.232702000	-0.080243000
1	3.715050000	2.134433000	0.037971000
1	2.690113000	4.387057000	-0.038414000
1	0.182378000	4.588665000	-0.141906000
1	-1.178251000	2.480479000	-0.163991000
1	-2.632766000	-1.738592000	-0.281026000
6	-3.924019000	-2.037470000	-0.158709000
1	-3.826537000	-2.574190000	0.791557000
1	-3.923959000	-2.669590000	-1.054024000
1	-4.763183000	-1.340565000	-0.161721000
6	-2.969820000	0.286539000	0.691421000
8	-3.237362000	1.460803000	0.605065000
8	-2.894935000	-0.381196000	1.854639000
1	-3.114684000	0.249117000	2.563352000
7	-3.004865000	-0.071047000	-1.720638000
1	-3.684465000	0.686712000	-1.712752000
1	-2.305553000	0.007142000	-2.447934000
j(NH_2/CN)			
29	-0.788608000	-0.463299000	-0.043063000
6	-2.912868000	-0.597644000	-0.224815000
6	0.872789000	3.622169000	-0.018859000
6	2.110498000	-0.424083000	0.026027000

6	3.436900000	-0.837078000	0.056700000
6	3.729183000	-2.190128000	0.077915000
6	2.691850000	-3.106581000	0.068676000
6	1.397232000	-2.621148000	0.037748000
7	1.110895000	-1.319600000	0.016763000
1	4.240120000	-0.118269000	0.065203000
1	4.757737000	-2.521923000	0.102011000
1	2.873571000	-4.170524000	0.085168000
1	0.553564000	-3.298188000	0.029893000
6	1.723948000	1.018444000	0.006105000
6	2.663384000	2.040194000	0.007220000
6	2.229732000	3.356841000	-0.006220000
6	-0.005368000	2.549963000	-0.020380000
7	0.410462000	1.285063000	-0.009454000
1	3.720193000	1.827448000	0.018249000
1	2.949560000	4.163380000	-0.005660000
1	0.492081000	4.632331000	-0.027289000
1	-1.076879000	2.704012000	-0.025666000
1	-2.814879000	-1.271356000	0.757703000
6	-4.089970000	-1.339106000	1.213140000
1	-3.926233000	-0.865953000	2.177214000
1	-4.051482000	-2.426108000	1.235080000
1	-4.980053000	-0.974876000	0.720011000
6	-3.211652000	0.778564000	0.053294000
7	-3.407339000	1.891797000	0.264595000
7	-3.336491000	-1.124629000	-1.412564000
1	-4.279081000	-0.975489000	-1.739676000
1	-2.845235000	-1.899684000	-1.818087000
k(OH/COCl)			
29	0.294787000	0.473510000	0.262405000
6	2.382945000	0.820132000	0.353343000
6	-3.203799000	3.072158000	-0.232652000
6	-2.190127000	-1.050248000	-0.023293000
6	-3.111301000	-2.095042000	-0.110805000
6	-2.655646000	-3.408791000	-0.039001000
6	-1.295240000	-3.648522000	0.117576000
6	-0.439828000	-2.553452000	0.199787000
7	-0.875122000	-1.290656000	0.132499000
1	-4.174233000	-1.899662000	-0.232777000
1	-3.362680000	-4.235794000	-0.106420000
1	-0.896024000	-4.659871000	0.175338000
1	0.637508000	-2.682851000	0.320380000
6	-2.596312000	0.389191000	-0.099283000
6	-3.921690000	0.782888000	-0.291452000
6	-4.227140000	2.139006000	-0.357892000
6	-1.908509000	2.601783000	-0.044298000
7	-1.610240000	1.299239000	0.022412000
1	-4.715475000	0.046474000	-0.392878000
1	-5.257979000	2.460033000	-0.508407000
1	-3.396132000	4.142739000	-0.280656000
1	-1.070503000	3.293341000	0.056229000
1	2.204027000	0.707307000	1.573901000
6	3.375202000	0.632367000	2.141423000
1	3.297434000	-0.378440000	2.555744000
1	3.127419000	1.443118000	2.838663000
1	4.324696000	0.814897000	1.633424000
6	3.012835000	-0.436761000	-0.178952000
17	4.159608000	-0.130336000	-1.505836000
8	2.772073000	-1.536414000	0.200369000
8	2.777228000	2.024233000	-0.066812000
1	3.694553000	2.001751000	-0.402905000
l(CH_3/COCl)			
29	0.397316000	-0.411938000	0.418965000
6	2.381801000	-0.283324000	0.745118000
6	-1.410677000	3.609471000	0.133764000
6	-2.464224000	-0.499251000	-0.073122000
6	-3.758691000	-0.978916000	-0.277399000
6	-3.975804000	-2.352171000	-0.337670000
6	-2.896363000	-3.216538000	-0.192409000
6	-1.637102000	-2.662879000	0.011459000
7	-1.422144000	-1.343292000	0.070835000
1	-4.596886000	-0.294658000	-0.387173000
1	-4.982138000	-2.739905000	-0.496723000
1	-3.018972000	-4.297576000	-0.233667000
1	-0.759697000	-3.299838000	0.132616000
6	-2.152015000	0.962653000	-0.004065000
6	-3.119754000	1.951550000	-0.184497000
6	-2.741390000	3.289873000	-0.112443000
6	-0.505237000	2.565661000	0.301475000
7	-0.867277000	1.280499000	0.236872000
1	-4.157625000	1.694701000	-0.384465000
1	-3.484861000	4.074833000	-0.251992000
1	-1.072203000	4.642618000	0.193380000
1	0.553522000	2.760132000	0.487747000
1	2.729063000	-1.748180000	0.080300000
6	3.673375000	-2.379142000	0.020156000
1	3.541875000	-2.965560000	-0.897515000
1	3.685633000	-3.008568000	0.917580000
1	4.565810000	-1.749679000	-0.045822000
6	3.071133000	-0.297434000	2.055384000

1	2.693660000	-1.074019000	2.728636000
1	4.169575000	-0.286729000	2.011135000
1	2.762208000	0.684031000	2.472431000
6	2.924744000	0.696826000	-0.237634000
8	3.025099000	1.841413000	0.083821000
17	3.137369000	0.166152000	-1.905275000
m(NH_2/COCl)			
29	-0.406068000	-0.671171000	-0.243005000
6	-2.507768000	-0.898735000	-0.573172000
6	0.782421000	3.609524000	-0.264410000
6	2.456524000	-0.282983000	0.023536000
6	3.824962000	-0.542169000	0.122766000
6	4.266827000	-1.857818000	0.219547000
6	3.333460000	-2.888666000	0.214122000
6	1.989021000	-2.552177000	0.104807000
7	1.558021000	-1.288283000	0.010717000
1	4.548549000	0.269538000	0.118300000
1	5.332874000	-2.072024000	0.296898000
1	3.632355000	-3.932900000	0.289364000
1	1.220133000	-3.326030000	0.092187000
6	1.913237000	1.109248000	-0.073846000
6	2.728166000	2.237057000	0.035693000
6	2.153379000	3.501326000	-0.062641000
6	0.036896000	2.437504000	-0.356514000
7	0.586005000	1.221860000	-0.265031000
1	3.798484000	2.144588000	0.204987000
1	2.775984000	4.392113000	0.022074000
1	0.290326000	4.577338000	-0.345905000
1	-1.044340000	2.473842000	-0.508397000
1	-2.413073000	-1.765783000	0.276556000
6	-3.674339000	-2.067509000	0.597979000
1	-3.443999000	-2.176642000	1.664882000
1	-3.706201000	-3.001681000	0.028497000
1	-4.557735000	-1.448760000	0.431269000
6	-2.887163000	0.423205000	-0.027736000
17	-2.551139000	0.612705000	1.730417000
8	-3.277960000	1.339949000	-0.675088000
7	-2.940059000	-1.217293000	-1.828645000
1	-3.730217000	-0.693684000	-2.198248000
1	-2.255646000	-1.492363000	-2.521772000
Ag carbenoid reactions			
a(H/H)			
47	-1.170943000	-0.808265000	0.034929000
6	-3.085068000	-1.763243000	0.067457000
1	-3.625072000	-2.169192000	-0.805251000

1	-3.671256000	-2.014349000	0.968208000
6	3.087408000	-2.550957000	-0.021546000
6	1.248124000	1.280492000	0.001979000
6	1.967135000	2.475244000	0.097424000
6	1.282138000	3.685869000	0.099606000
6	-0.105424000	3.678639000	0.008909000
6	-0.747735000	2.447365000	-0.068635000
7	-0.094337000	1.280613000	-0.070379000
1	3.051216000	2.471198000	0.184074000
1	1.831008000	4.624694000	0.175308000
1	-0.683957000	4.601048000	0.004657000
1	-1.835823000	2.389632000	-0.132582000
6	1.930440000	-0.052131000	-0.017611000
6	3.317674000	-0.167047000	-0.140997000
6	3.901005000	-1.430004000	-0.141719000
6	1.714483000	-2.353488000	0.082966000
7	1.148402000	-1.141545000	0.083230000
1	3.944847000	0.714671000	-0.250321000
1	4.981841000	-1.533254000	-0.238933000
1	3.498351000	-3.559290000	-0.014861000
1	1.035469000	-3.203241000	0.170890000
1	-4.333635000	0.025415000	0.799963000
6	-4.792714000	0.313121000	-0.159434000
1	-5.849477000	0.022284000	-0.155430000
1	-4.294642000	-0.140085000	-1.031362000
1	-4.697786000	1.402285000	-0.256401000
b(OH/Cl)			
47	-0.749082000	-0.259612000	-0.189576000
6	-3.048887000	0.140602000	-0.138448000
6	2.655696000	-3.337695000	-0.024529000
6	2.235912000	0.892771000	0.027460000
6	3.329913000	1.757376000	-0.069849000
6	3.113447000	3.131715000	-0.043114000
6	1.813165000	3.610750000	0.074788000
6	0.778127000	2.682946000	0.145439000
7	0.981910000	1.362450000	0.121841000
1	4.340375000	1.371331000	-0.186993000
1	3.955704000	3.819535000	-0.120095000
1	1.597230000	4.677714000	0.102108000
1	-0.262291000	3.005650000	0.223169000
6	2.410326000	-0.595114000	0.023636000
6	3.657676000	-1.179153000	0.262412000
6	3.781367000	-2.564221000	0.236558000
6	1.446914000	-2.682563000	-0.233449000
7	1.321222000	-1.350309000	-0.208837000

1	4.524921000	-0.562713000	0.489246000
1	4.748073000	-3.031565000	0.424922000
1	2.702266000	-4.425054000	-0.057482000
1	0.535437000	-3.249544000	-0.429940000
1	-3.117514000	-0.608934000	-1.061892000
6	-4.444233000	-0.598696000	-1.329708000
1	-4.555550000	-1.689096000	-1.283133000
1	-4.317299000	-0.196356000	-2.342398000
1	-5.227009000	-0.087347000	-0.765363000
17	-3.673274000	-0.464432000	1.399032000
8	-3.211668000	1.459092000	-0.353090000
1	-4.076959000	1.763467000	-0.019096000
c(OH/COOH)			
47	0.607082000	-0.161568000	0.239813000
6	2.868481000	0.322359000	0.308098000
6	-2.149091000	3.569456000	-0.119523000
6	-2.532228000	-0.660611000	-0.052543000
6	-3.741883000	-1.311860000	-0.310799000
6	-3.792383000	-2.701268000	-0.274742000
6	-2.632860000	-3.411848000	0.015549000
6	-1.465048000	-2.691676000	0.242584000
7	-1.409817000	-1.354947000	0.208517000
1	-4.635974000	-0.744294000	-0.559796000
1	-4.729050000	-3.220813000	-0.477514000
1	-2.622961000	-4.499846000	0.057861000
1	-0.528595000	-3.207070000	0.462760000
6	-2.434891000	0.834195000	-0.064949000
6	-3.573642000	1.642465000	-0.003211000
6	-3.426182000	3.025933000	-0.033503000
6	-1.066921000	2.694858000	-0.156824000
7	-1.204082000	1.365880000	-0.130072000
1	-4.566115000	1.206238000	0.089153000
1	-4.303867000	3.670648000	0.016095000
1	-1.986561000	4.645830000	-0.147679000
1	-0.042871000	3.070651000	-0.208748000
1	2.919631000	-0.402052000	1.295926000
6	4.191111000	-0.553585000	1.605982000
1	4.341267000	-1.610761000	1.366446000
1	3.964778000	-0.354525000	2.661835000
1	4.982632000	0.101758000	1.237285000
6	3.607052000	-0.198552000	-0.892047000
8	4.240453000	0.545993000	-1.595393000
8	3.448561000	-1.508199000	-1.094423000
1	3.957997000	-1.747896000	-1.890023000
8	3.069143000	1.629397000	0.527452000
1	3.839755000	1.912581000	-0.009428000
d(OH/CN)			
47	0.790915000	-0.577515000	0.065111000
6	3.135028000	-0.466833000	0.224314000
6	-3.084310000	-3.022018000	-0.072473000
6	-1.944953000	1.077085000	-0.006015000
6	-2.873955000	2.106308000	0.167319000
6	-2.431343000	3.426170000	0.173654000
6	-1.075819000	3.686236000	0.010320000
6	-0.211320000	2.604182000	-0.135231000
7	-0.633676000	1.335347000	-0.140850000
1	-3.929509000	1.890161000	0.317979000
1	-3.143062000	4.240497000	0.310713000
1	-0.684272000	4.702331000	0.005177000
1	0.864219000	2.757280000	-0.251674000
6	-2.369454000	-0.359680000	-0.043768000
6	-3.708168000	-0.713476000	-0.238260000
6	-4.068957000	-2.056186000	-0.250988000
6	-1.772949000	-2.592806000	0.095219000
7	-1.418992000	-1.301268000	0.106958000
1	-4.465176000	0.050640000	-0.400257000
1	-5.109635000	-2.342242000	-0.404506000
1	-3.317501000	-4.085586000	-0.071163000
1	-0.966303000	-3.315727000	0.227631000
1	3.229301000	-1.205885000	-0.742105000
6	4.468268000	-1.069010000	-1.173185000
1	4.330337000	-0.471071000	-2.079351000
1	4.509027000	-2.153725000	-1.346507000
1	5.275743000	-0.728008000	-0.523476000
8	3.772955000	-0.837082000	1.360302000
1	3.286620000	-1.556756000	1.786026000
6	3.285079000	0.936803000	-0.098066000
7	3.292763000	2.068392000	-0.350128000
e(CH_3/Cl)			
47	0.735285000	-0.425082000	0.030734000
6	3.022322000	-0.313101000	0.195532000
6	-2.934643000	-3.186397000	-0.068215000
6	-2.160060000	0.992609000	-0.010031000
6	-3.162714000	1.945983000	-0.209135000
6	-2.829981000	3.296848000	-0.180051000
6	-1.507090000	3.663387000	0.041948000
6	-0.567033000	2.650903000	0.211588000
7	-0.881803000	1.352497000	0.186240000
1	-4.189666000	1.645844000	-0.407057000
1	-3.599272000	4.053342000	-0.336122000

1	-1.201028000	4.707923000	0.074600000
1	0.486684000	2.886999000	0.374237000
6	-2.461701000	-0.474252000	-0.016145000
6	-3.770400000	-0.947548000	0.116843000
6	-4.008818000	-2.317381000	0.089001000
6	-1.661175000	-2.637980000	-0.174805000
7	-1.424634000	-1.320955000	-0.147493000
1	-4.598263000	-0.256903000	0.262610000
1	-5.024448000	-2.699064000	0.194865000
1	-3.070871000	-4.266264000	-0.099233000
1	-0.787613000	-3.282160000	-0.288627000
1	3.110486000	-1.113361000	-0.768134000
6	4.298426000	-1.421294000	-1.056195000
1	4.161588000	-1.251254000	-2.132387000
1	4.295470000	-2.477346000	-0.763647000
1	5.138332000	-0.855926000	-0.654051000
17	3.532591000	1.300418000	-0.310794000
6	3.653328000	-0.741102000	1.491756000
1	4.733209000	-0.534286000	1.552438000
1	3.154739000	-0.164398000	2.285796000
1	3.460500000	-1.806217000	1.679923000
f(CH_3/COOH)			
47	0.586158000	-0.498881000	0.205800000
6	2.788258000	-0.201596000	0.413245000
6	-1.509646000	3.651297000	0.071146000
6	-2.586290000	-0.455422000	-0.090268000
6	-3.892661000	-0.888864000	-0.334278000
6	-4.165996000	-2.251616000	-0.380926000
6	-3.127817000	-3.155724000	-0.185360000
6	-1.852663000	-2.647178000	0.035951000
7	-1.582920000	-1.336826000	0.080438000
1	-4.692643000	-0.172134000	-0.505749000
1	-5.180845000	-2.600386000	-0.572872000
1	-3.291905000	-4.231841000	-0.209971000
1	-1.005645000	-3.318787000	0.185751000
6	-2.248781000	1.003043000	-0.019092000
6	-3.238163000	1.985383000	0.078383000
6	-2.860347000	3.324390000	0.122368000
6	-0.585199000	2.612649000	-0.006908000
7	-0.947150000	1.326840000	-0.048797000
1	-4.291521000	1.719958000	0.138170000
1	-3.619220000	4.103214000	0.200579000
1	-1.171101000	4.685913000	0.099534000
1	0.489834000	2.806801000	-0.039422000
1	3.278459000	-1.592471000	-0.304192000

6	4.246015000	-2.185566000	-0.340083000
1	4.213343000	-2.693275000	-1.311623000
1	4.208531000	-2.894286000	0.494988000
1	5.111408000	-1.520887000	-0.277979000
6	3.468409000	-0.149639000	1.729733000
1	3.176689000	-0.965909000	2.398765000
1	4.561116000	-0.025362000	1.688953000
1	3.054735000	0.791094000	2.150691000
6	3.254427000	0.833490000	-0.547242000
8	2.816208000	1.945952000	-0.364585000
8	4.055014000	0.469321000	-1.537426000
1	4.212785000	1.255595000	-2.093643000
g(CH_3/CN)			
47	0.783393000	-0.578359000	0.076387000
6	3.046479000	-0.437456000	0.279017000
6	-1.110111000	3.695057000	0.000496000
6	-2.386013000	-0.355733000	-0.025217000
6	-3.730824000	-0.720526000	0.087905000
6	-4.080321000	-2.066360000	0.071890000
6	-3.078481000	-3.022639000	-0.054376000
6	-1.762800000	-2.582075000	-0.142351000
7	-1.419612000	-1.287731000	-0.125621000
1	-4.501758000	0.037338000	0.209657000
1	-5.125660000	-2.362134000	0.162793000
1	-3.302036000	-4.088079000	-0.075442000
1	-0.943317000	-3.297147000	-0.231593000
6	-1.970205000	1.083456000	-0.031653000
6	-2.889983000	2.108761000	-0.268324000
6	-2.452009000	3.429867000	-0.248678000
6	-0.255140000	2.615339000	0.206310000
7	-0.672688000	1.345729000	0.190695000
1	-3.932488000	1.887834000	-0.488173000
1	-3.155548000	4.241834000	-0.433685000
1	-0.723133000	4.712604000	0.026378000
1	0.810948000	2.772350000	0.387877000
1	3.301188000	-0.885485000	-1.080753000
6	4.260516000	-0.814171000	-1.748091000
1	3.994017000	-0.052056000	-2.489112000
1	4.307989000	-1.829503000	-2.161646000
1	5.156855000	-0.550145000	-1.183681000
6	3.371677000	0.960306000	0.232810000
7	3.510498000	2.114666000	0.236530000
6	3.897410000	-1.266532000	1.182995000
1	3.499124000	-1.025136000	2.188478000
1	3.739163000	-2.339350000	1.025336000

1	4.966656000	-1.012447000	1.185757000
$h(NH_2/Cl)$			
47	0.732329000	-0.477308000	0.033745000
6	3.052142000	-0.412197000	0.211286000
6	-1.370639000	3.677151000	0.048784000
6	-2.457905000	-0.428042000	-0.018048000
6	-3.781620000	-0.858435000	0.111549000
6	-4.064971000	-2.219700000	0.079946000
6	-3.019812000	-3.123447000	-0.077492000
6	-1.728589000	-2.617370000	-0.180318000
7	-1.449345000	-1.308728000	-0.149269000
1	-4.586593000	-0.141270000	0.257484000
1	-5.092856000	-2.567928000	0.183050000
1	-3.191442000	-4.198156000	-0.111657000
1	-0.876380000	-3.289574000	-0.294071000
6	-2.108904000	1.028487000	-0.008643000
6	-3.079363000	2.013685000	-0.211558000
6	-2.703496000	3.353184000	-0.179654000
6	-0.463834000	2.635273000	0.221731000
7	-0.820704000	1.347704000	0.193911000
1	-4.114396000	1.746960000	-0.415163000
1	-3.447439000	4.133973000	-0.338909000
1	-1.031471000	4.711345000	0.083423000
1	0.596623000	2.836022000	0.388266000
1	3.007135000	-1.141482000	-0.752646000
6	4.276890000	-1.317762000	-1.058339000
1	4.145859000	-1.039846000	-2.112002000
1	4.269888000	-2.395408000	-0.856330000
1	5.134551000	-0.813129000	-0.611576000
17	3.462821000	1.270184000	-0.265114000
7	3.583682000	-0.887655000	1.387833000
1	4.475396000	-0.485349000	1.679137000
1	2.928869000	-0.960042000	2.159123000
$i(NH_2/COOH)$			
47	-0.570464000	-0.727650000	-0.222363000
6	-2.875204000	-0.416036000	-0.443735000
6	0.969119000	3.685171000	-0.104080000
6	2.550206000	-0.253818000	0.065440000
6	3.896118000	-0.506975000	0.345987000
6	4.353311000	-1.819634000	0.389657000
6	3.455626000	-2.855352000	0.154770000
6	2.128974000	-2.524947000	-0.097967000
7	1.682563000	-1.263160000	-0.139442000
1	4.581698000	0.312393000	0.551572000
1	5.400232000	-2.028190000	0.610831000
1	3.765608000	-3.898928000	0.174581000
1	1.387224000	-3.304999000	-0.277012000
6	2.025900000	1.148343000	-0.008698000
6	2.884350000	2.240454000	-0.163177000
6	2.346230000	3.523461000	-0.208650000
6	0.181190000	2.544125000	0.025956000
7	0.696115000	1.311158000	0.071938000
1	3.958058000	2.099662000	-0.270192000
1	3.000966000	4.386464000	-0.331418000
1	0.505859000	4.670315000	-0.132183000
1	-0.907525000	2.614468000	0.097834000
1	-3.006670000	-1.582924000	-0.139005000
6	-4.314366000	-1.733767000	0.080588000
1	-4.208710000	-2.216580000	1.058286000
1	-4.440118000	-2.415615000	-0.767953000
1	-5.068698000	-0.945919000	0.079756000
6	-3.055402000	0.533063000	0.686069000
8	-3.160009000	1.724760000	0.520281000
8	-3.026386000	-0.054807000	1.894088000
1	-3.121954000	0.649848000	2.558889000
7	-3.280952000	0.002967000	-1.682117000
1	-3.864617000	0.836333000	-1.704183000
1	-2.638783000	-0.077679000	-2.459317000
$j(NH_2/CN)$			
47	0.799310000	-0.566286000	0.057674000
6	3.167644000	-0.459682000	0.204914000
6	-1.095189000	3.683547000	0.016408000
6	-2.366269000	-0.369307000	-0.042001000
6	-3.703282000	-0.729142000	-0.237888000
6	-4.057543000	-2.073545000	-0.252509000
6	-3.068279000	-3.034726000	-0.074426000
6	-1.759031000	-2.599209000	0.094356000
7	-1.411447000	-1.306218000	0.107818000
1	-4.463753000	0.031588000	-0.399715000
1	-5.096692000	-2.364495000	-0.407201000
1	-3.296306000	-4.099421000	-0.074523000
1	-0.948722000	-3.318144000	0.226303000
6	-1.949137000	1.069676000	-0.001884000
6	-2.883690000	2.093171000	0.175760000
6	-2.448708000	3.415522000	0.183085000
6	-0.224473000	2.606839000	-0.132828000
7	-0.639806000	1.335766000	-0.139245000
1	-3.937513000	1.870436000	0.328998000
1	-3.164768000	4.225505000	0.323327000
1	-0.709597000	4.701918000	0.011471000

1	0.849994000	2.765418000	-0.252094000
1	3.196399000	-1.177849000	-0.759479000
6	4.474205000	-1.059307000	-1.202435000
1	4.249434000	-0.642220000	-2.191110000
1	4.606051000	-2.149158000	-1.193136000
1	5.296630000	-0.540895000	-0.706877000
6	3.250298000	0.943616000	-0.120137000
7	3.257238000	2.077489000	-0.365594000
7	3.641171000	-0.861752000	1.425952000
1	4.572075000	-0.578053000	1.723613000
1	3.329842000	-1.750935000	1.794719000
Au carbenoid reactions			
a(H/H)			
79	-0.955638000	-0.600180000	0.000022000
6	-2.679063000	-1.332503000	0.000033000
1	-3.247568000	-1.580397000	-0.909331000
1	-3.247552000	-1.580452000	0.909395000
6	3.114075000	-2.598385000	-0.000040000
6	1.559863000	1.344456000	-0.000002000
6	2.315470000	2.516748000	-0.000024000
6	1.660590000	3.744978000	-0.000015000
6	0.270027000	3.774245000	0.000014000
6	-0.413898000	2.563139000	0.000034000
7	0.215591000	1.383128000	0.000027000
1	3.402765000	2.481252000	-0.000048000
1	2.236026000	4.670762000	-0.000032000
1	-0.282246000	4.712501000	0.000021000
1	-1.504299000	2.529453000	0.000057000
6	2.165560000	-0.016056000	-0.000012000
6	3.542600000	-0.236459000	-0.000016000
6	4.020591000	-1.543695000	-0.000030000
6	1.755674000	-2.299518000	-0.000034000
7	1.295977000	-1.042885000	-0.000019000
1	4.241511000	0.597172000	-0.000007000
1	5.094080000	-1.733034000	-0.000033000
1	3.443454000	-3.636111000	-0.000051000
1	1.003461000	-3.089014000	-0.000040000
1	-3.914232000	0.743584000	0.000334000
6	-5.009464000	0.611642000	-0.000121000
1	-5.470127000	1.607338000	-0.001054000
1	-5.326920000	0.069579000	0.899183000
1	-5.326110000	0.068327000	-0.898956000
b(OH/Cl)			
79	-0.684488000	-0.388410000	-0.130140000
6	-2.684524000	0.260634000	-0.173843000
6	2.659400000	-3.246294000	0.108532000
6	2.289506000	0.991901000	0.039949000
6	3.375475000	1.838097000	-0.200986000
6	3.163912000	3.214134000	-0.203199000
6	1.882241000	3.703154000	0.026504000
6	0.855681000	2.784575000	0.236542000
7	1.055421000	1.466489000	0.246123000
1	4.367320000	1.440396000	-0.407795000
1	3.994396000	3.894872000	-0.391015000
1	1.673680000	4.772076000	0.033887000
1	-0.171818000	3.114661000	0.402820000
6	2.443443000	-0.494170000	0.072602000
6	3.695202000	-1.090173000	0.245177000
6	3.806805000	-2.475205000	0.262193000
6	1.445195000	-2.591359000	-0.042790000
7	1.334666000	-1.252160000	-0.058098000
1	4.578140000	-0.471375000	0.389755000
1	4.780450000	-2.945361000	0.401132000
1	2.692682000	-4.334398000	0.113217000
1	0.517317000	-3.151090000	-0.157714000
1	-2.679093000	-0.797780000	-0.778828000
6	-3.989216000	-0.773927000	-1.079301000
1	-4.135064000	-1.784807000	-0.676155000
1	-3.796906000	-0.741101000	-2.159275000
1	-4.803825000	-0.110873000	-0.777654000
17	-3.386359000	0.252454000	1.450973000
8	-2.764313000	1.431847000	-0.839953000
1	-3.649458000	1.824133000	-0.722679000
c(OH/COOH)			
79	0.563483000	-0.356549000	0.213734000
6	2.540792000	0.373364000	0.307652000
6	-2.089697000	3.684969000	-0.129306000
6	-2.552458000	-0.522943000	-0.117348000
6	-3.784284000	-1.146947000	-0.330710000
6	-3.868508000	-2.533961000	-0.324760000
6	-2.713857000	-3.278798000	-0.107715000
6	-1.519851000	-2.597191000	0.081509000
7	-1.435849000	-1.255820000	0.075222000
1	-4.670715000	-0.546901000	-0.525831000
1	-4.826053000	-3.026270000	-0.495385000
1	-2.726221000	-4.367250000	-0.092040000
1	-0.586855000	-3.135627000	0.246689000
6	-2.431588000	0.966371000	-0.107690000
6	-3.538227000	1.788387000	0.122936000
6	-3.359645000	3.169122000	0.106943000

6	-1.041256000	2.788396000	-0.328134000
7	-1.209010000	1.465867000	-0.322220000
1	-4.519169000	1.367777000	0.337517000
1	-4.205724000	3.832576000	0.287003000
1	-1.906536000	4.758403000	-0.149676000
1	-0.021510000	3.140503000	-0.496719000
1	2.521858000	-0.452576000	1.200385000
6	3.833537000	-0.424514000	1.505284000
1	4.081083000	-1.449832000	1.211590000
1	3.543601000	-0.314167000	2.559095000
1	4.597485000	0.307663000	1.233316000
6	3.343693000	0.084274000	-0.938938000
8	3.916890000	0.978757000	-1.501829000
8	3.345231000	-1.195166000	-1.299838000
1	3.898016000	-1.278827000	-2.098752000
8	2.562562000	1.674315000	0.674852000
1	3.310191000	2.095194000	0.202999000
d(OH/CN)			
79	0.733018000	-0.549146000	0.053644000
6	2.794094000	-0.060801000	0.232335000
6	-2.848169000	-3.073024000	-0.137395000
6	-2.097209000	1.117529000	-0.008640000
6	-3.097281000	2.037592000	0.316672000
6	-2.781922000	3.394101000	0.321159000
6	-1.486797000	3.788133000	0.005106000
6	-0.546764000	2.799613000	-0.284300000
7	-0.844695000	1.498984000	-0.289070000
1	-4.100001000	1.711291000	0.586765000
1	-3.543100000	4.131933000	0.575381000
1	-1.198134000	4.838197000	-0.008705000
1	0.487337000	3.059087000	-0.522905000
6	-2.378360000	-0.349677000	-0.060801000
6	-3.684114000	-0.828275000	-0.200846000
6	-3.923677000	-2.196128000	-0.238239000
6	-1.575403000	-2.535571000	-0.017402000
7	-1.341118000	-1.211109000	0.016187000
1	-4.509952000	-0.128014000	-0.305596000
1	-4.940711000	-2.571721000	-0.352116000
1	-2.981786000	-4.153170000	-0.159048000
1	-0.700164000	-3.180395000	0.056870000
1	2.838049000	-0.969068000	-0.573138000
6	4.127075000	-0.815805000	-0.901748000
1	4.009417000	-0.440099000	-1.923220000
1	4.202750000	-1.910369000	-0.824809000
1	4.918793000	-0.326352000	-0.330435000
8	3.375600000	-0.260804000	1.450417000
1	2.768867000	-0.748372000	2.024624000
6	2.911273000	1.296740000	-0.275647000
7	2.951380000	2.384910000	-0.668029000
e(CH_3/Cl)			
79	0.675958000	-0.533790000	0.021256000
6	2.701081000	-0.050713000	0.137969000
6	-2.893176000	-3.094421000	-0.063575000
6	-2.195633000	1.100462000	0.019798000
6	-3.166296000	2.019510000	-0.389165000
6	-2.869770000	3.378106000	-0.323129000
6	-1.620484000	3.774519000	0.142106000
6	-0.709100000	2.785202000	0.507907000
7	-0.987434000	1.482430000	0.450600000
1	-4.127764000	1.687427000	-0.777660000
1	-3.606881000	4.116258000	-0.639756000
1	-1.347449000	4.826540000	0.213092000
1	0.289421000	3.047832000	0.863919000
6	-2.460055000	-0.369135000	-0.006200000
6	-3.765246000	-0.865998000	0.042810000
6	-3.986330000	-2.237366000	0.012293000
6	-1.623153000	-2.536141000	-0.093811000
7	-1.406032000	-1.209691000	-0.063498000
1	-4.603714000	-0.178037000	0.131505000
1	-5.001985000	-2.631050000	0.055561000
1	-3.011552000	-4.176237000	-0.091034000
1	-0.735361000	-3.166054000	-0.146089000
1	2.759667000	-0.910533000	-0.750733000
6	4.014347000	-1.047918000	-0.964779000
1	3.845312000	-0.985828000	-2.049014000
1	4.118221000	-2.070144000	-0.583724000
1	4.809194000	-0.375497000	-0.642818000
17	2.996004000	1.573213000	-0.499934000
6	3.341154000	-0.294597000	1.483138000
1	4.391413000	0.031279000	1.513666000
1	2.774769000	0.286135000	2.224099000
1	3.264160000	-1.355486000	1.756775000
f(CH_3/COOH)			
79	0.565561000	-0.588180000	0.167693000
6	2.540999000	-0.046600000	0.377889000
6	-1.575574000	3.785000000	0.077552000
6	-2.552639000	-0.329237000	-0.119701000
6	-3.858189000	-0.778426000	-0.335684000
6	-4.121214000	-2.141169000	-0.402087000
6	-3.068938000	-3.038568000	-0.252573000

6	-1.794116000	-2.526442000	-0.056328000
7	-1.535863000	-1.208835000	0.004732000
1	-4.662468000	-0.059138000	-0.476333000
1	-5.137156000	-2.496468000	-0.575389000
1	-3.221161000	-4.115711000	-0.293497000
1	-0.936429000	-3.188873000	0.060114000
6	-2.246910000	1.131294000	-0.031933000
6	-3.234321000	2.064322000	0.297317000
6	-2.887912000	3.412130000	0.348361000
6	-0.652500000	2.783551000	-0.221648000
7	-0.982371000	1.492269000	-0.277568000
1	-4.249536000	1.751820000	0.536423000
1	-3.637705000	4.160287000	0.606460000
1	-1.263794000	4.828223000	0.106274000
1	0.395775000	3.018430000	-0.423768000
1	2.901428000	-1.251873000	0.026185000
6	4.091356000	-1.487857000	0.102924000
1	4.097120000	-2.124708000	-0.791069000
1	4.145653000	-2.032672000	1.049997000
1	4.806481000	-0.670846000	0.021828000
6	2.969257000	0.406071000	1.748738000
1	2.733526000	-0.337830000	2.517865000
1	4.028936000	0.695941000	1.799805000
1	2.368790000	1.305889000	1.952596000
6	2.885756000	0.902549000	-0.737133000
8	2.719363000	2.086516000	-0.603024000
8	3.304188000	0.310314000	-1.859130000
1	3.420126000	1.005393000	-2.532312000
	g(CH_3/CN)		
79	-0.733054000	-0.554745000	-0.000147000
6	-2.752102000	-0.087603000	-0.143606000
6	1.521823000	3.790282000	-0.065397000
6	2.392799000	-0.349480000	-0.005038000
6	3.701635000	-0.832563000	-0.089421000
6	3.939069000	-2.201313000	-0.079770000
6	2.858091000	-3.072412000	0.011988000
6	1.583264000	-2.529453000	0.076489000
7	1.350238000	-1.204651000	0.065277000
1	4.530131000	-0.134256000	-0.189558000
1	4.957982000	-2.582264000	-0.151287000
1	2.989290000	-4.153006000	0.025055000
1	0.704359000	-3.170551000	0.141642000
6	2.116343000	1.118694000	-0.003569000
6	3.089916000	2.036493000	0.400633000
6	2.783428000	3.394375000	0.364445000

6	0.607198000	2.802486000	-0.429599000
7	0.896845000	1.500612000	-0.402240000
1	4.061426000	1.704610000	0.763458000
1	3.522855000	4.131580000	0.677946000
1	1.239391000	4.841234000	-0.110374000
1	-0.402641000	3.066284000	-0.752121000
1	-2.874762000	-0.556645000	1.038344000
6	-4.016248000	-0.410965000	1.502833000
1	-3.704408000	0.147404000	2.394025000
1	-4.206563000	-1.478360000	1.670352000
1	-4.810409000	0.086194000	0.947205000
6	-2.898739000	1.354009000	-0.116750000
7	-2.967480000	2.510619000	-0.142600000
6	-3.565754000	-0.808305000	-1.195601000
1	-3.077488000	-0.598667000	-2.159405000
1	-3.533817000	-1.893060000	-1.038321000
1	-4.605654000	-0.457166000	-1.254243000
	h(NH_2/Cl)		
79	0.672757000	-0.545857000	0.036022000
6	2.724842000	-0.090588000	0.168667000
6	-1.553279000	3.783996000	0.148138000
6	-2.453843000	-0.346616000	-0.013128000
6	-3.765109000	-0.827459000	0.027713000
6	-4.002633000	-2.196022000	-0.010657000
6	-2.919972000	-3.066402000	-0.085750000
6	-1.642797000	-2.524226000	-0.107585000
7	-1.410489000	-1.200436000	-0.070250000
1	-4.595594000	-0.129825000	0.115946000
1	-5.023246000	-2.577361000	0.026126000
1	-3.051691000	-4.146508000	-0.118916000
1	-0.762390000	-3.164576000	-0.158080000
6	-2.169177000	1.119049000	0.020370000
6	-3.126018000	2.053627000	-0.385385000
6	-2.808982000	3.407482000	-0.316225000
6	-0.656528000	2.780574000	0.511367000
7	-0.955349000	1.482238000	0.452399000
1	-4.092468000	1.737071000	-0.774386000
1	-3.535169000	4.157320000	-0.630596000
1	-1.263949000	4.831577000	0.220069000
1	0.346801000	3.027012000	0.865145000
1	2.670840000	-0.977587000	-0.677241000
6	3.961077000	-1.041852000	-0.922223000
1	3.803882000	-0.916243000	-2.001441000
1	4.031474000	-2.081212000	-0.579062000
1	4.796785000	-0.432598000	-0.572942000

17	2.988922000	1.544142000	-0.533852000
7	3.277375000	-0.370026000	1.408274000
1	4.172711000	0.087668000	1.586279000
1	2.636273000	-0.234279000	2.184073000
i(NH_2/COOH)			
79	-0.545163000	-0.653019000	-0.191947000
6	-2.593543000	-0.102687000	-0.404870000
6	1.341574000	3.829473000	-0.079895000
6	2.527847000	-0.231764000	0.118059000
6	3.853357000	-0.606624000	0.352441000
6	4.193836000	-1.952349000	0.416887000
6	3.198041000	-2.908638000	0.247265000
6	1.898488000	-2.471053000	0.034631000
7	1.566530000	-1.169622000	-0.024760000
1	4.612547000	0.156944000	0.509224000
1	5.225953000	-2.248872000	0.604452000
1	3.412009000	-3.975330000	0.285665000
1	1.082263000	-3.181060000	-0.097066000
6	2.143938000	1.211086000	0.029715000
6	3.084416000	2.193276000	-0.294291000
6	2.671949000	3.522341000	-0.345191000
6	0.468443000	2.782683000	0.212955000
7	0.861375000	1.508965000	0.269300000
1	4.114941000	1.932289000	-0.529611000
1	3.385019000	4.306988000	-0.598841000
1	0.978170000	4.855870000	-0.108088000
1	-0.590955000	2.967347000	0.410456000
1	-2.673856000	-1.302768000	-0.228815000
6	-4.008430000	-1.374912000	-0.141191000
1	-3.996662000	-1.898516000	0.821058000
1	-4.049132000	-2.021038000	-1.025820000
1	-4.778207000	-0.602211000	-0.186722000
6	-2.855241000	0.783043000	0.782992000
8	-2.971059000	1.975149000	0.667189000
8	-2.919955000	0.118932000	1.942353000
1	-3.058438000	0.774811000	2.649059000
7	-2.912517000	0.407198000	-1.660819000
1	-3.500038000	1.239060000	-1.603101000
1	-2.123381000	0.563290000	-2.277465000
j(NH_2/CN)			
79	0.731401000	-0.550808000	0.059172000
6	2.820532000	-0.041987000	0.237746000
6	-1.472712000	3.788452000	0.014758000
6	-2.378466000	-0.346127000	-0.059302000
6	-3.686308000	-0.818888000	-0.200817000
6	-3.931093000	-2.185546000	-0.245166000
6	-2.858703000	-3.066845000	-0.150077000
6	-1.583755000	-2.534748000	-0.028220000
7	-1.344747000	-1.211691000	0.012513000
1	-4.509457000	-0.114838000	-0.301356000
1	-4.949607000	-2.556741000	-0.360177000
1	-2.996338000	-4.146354000	-0.177656000
1	-0.710685000	-3.182888000	0.041577000
6	-2.091785000	1.120044000	-0.001868000
6	-3.088221000	2.042701000	0.327420000
6	-2.768242000	3.398079000	0.333749000
6	-0.536333000	2.797447000	-0.278130000
7	-0.838710000	1.497985000	-0.283481000
1	-4.091480000	1.719115000	0.598800000
1	-3.526387000	4.137974000	0.591065000
1	-1.181055000	4.837688000	0.001209000
1	0.497785000	3.052902000	-0.520932000
1	2.779765000	-0.974230000	-0.531087000
6	4.098641000	-0.887036000	-0.895806000
1	3.884733000	-0.752346000	-1.963575000
1	4.245575000	-1.933994000	-0.599088000
1	4.923161000	-0.251784000	-0.564066000
6	2.893535000	1.278315000	-0.363476000
7	2.939977000	2.340736000	-0.821544000
7	3.269905000	-0.158657000	1.543158000
1	4.242014000	0.079097000	1.727768000
1	2.923792000	-0.941536000	2.083508000
